# Hybridization Drives Trait Integration in Telomere‐To‐Telomere *Apocynum* Genomes

**DOI:** 10.1111/pbi.70288

**Published:** 2025-08-06

**Authors:** Pan Xu, Fan Wu, Qi Yan, Bao Ao, Shengsheng Wang, Lijun Chen, Li Wang, Jiyu Zhang

**Affiliations:** ^1^ State Key Laboratory of Herbage Improvement and Grassland Agro‐Ecosystems, College of Pastoral Agriculture Science and Technology Lanzhou University Lanzhou China; ^2^ Key Laboratory of Grassland Resources of the Ministry of Education, College of Grassland Science Inner Mongolia Agricultural University Hohhot China

**Keywords:** *Apocynum*, ASE, flavonoid biosynthesis, hybrid speciation, stress adaptation, structural variation, T2T

## Abstract

Hybridization drives plant adaptation, yet its genomic mechanisms in non‐model perennials remain elusive. *Apocynum* species thrive in extreme saline‐alkaline environments. This study establishes *A. pictum* (APZ) as a homoploid hybrid of *A. venetum* (AVX) and *A. hendersonii* (AHG), exemplifying hybrid‐driven resilience. Leveraging telomere‐to‐telomere (T2T) genome assemblies of AVX, APZ, and AHG, we confirmed APZ's hybrid origin ~0.95 million years ago (Mya), following species divergence ~2.08 Mya, with AHG plastid inheritance. Nuclear and plastid analyses resolve taxonomic disputes among three *Apocynum* species. APZ exhibits large heterozygous inversions on chromosomes 3 and 8 with suppressed recombination, preserving AHG stress‐tolerance haplotypes. The study also showed that allele‐specific expression (ASE) dynamically regulates salt tolerance: AHG‐biased stress MAPK signalling pathway prevails at 200 mM NaCl, shifting to AVX‐bias at 400 mM NaCl, while flavonoid biosynthesis genes such as *AvFLS* and *AvCHS5* consistently favour AVX alleles. Transgenic assays validate AVX‐derived *AvFLS* for superior salt tolerance and ROS scavenging, with *AvCHS5* diversification driven by tandem duplication dosage effects. Homoploid hybrid speciation (HHS) analysis indicates *AvCHS5* and circadian *LHY* genes under positive selection enhance hybrid stability, supporting breeding potential. This study reveals how hybridization drives trait integration via dynamic ASE, identifying *AvFLS*, *AvCHS5*, and stress‐responsive loci as breeding targets for stress‐resilient, flavonoid‐rich cultivars, offering a genomic foundation for crop improvement in extreme environments.

## 1 Introduction

Hybridization drives plant adaptation by merging diverse genomes and enhancing resilience and innovation in extreme environments. This process fosters genetic diversity through gene introgression and species divergence, thereby shaping evolutionary trajectories (Goulet et al. 2017; Gu and Han 2024; Hochholdinger and Baldauf 2018; Liu, Zhang, Sang, et al. 2022; Soltis and Soltis 2009). Homoploid hybrid speciation (HHS) is a form of hybrid speciation without polyploidy that stabilises new species and their adaptive traits via alternate alleles of reproductive isolation genes (Wang et al. 2021). The genus *Apocynum* is a perennial subshrub that thrives in China's saline‐alkaline Gobi desert, exemplifying this potential by utilising flavonoids such as rutin, quercetin, and hyperoside to mitigate abiotic stress through reactive oxygen species (ROS) scavenging mechanisms, cellular homeostasis maintenance, and stress signalling modulation (Li et al. 2019; Thevs et al. 2012; Xu et al. 2021). Unlike halophytes that rely on sustained stress responses, *Apocynum* may adapt to the fluctuating salinity and drought in the Gobi desert (Pankova et al. 2015) through dynamic regulation, which is pronounced in its hybrid progeny, where allele‐specific expression (ASE) regulates stress tolerance and flavonoid production via differential parental allele expression (Jacob et al. 2010; Rieseberg and Carney 1998). For instance, *A. floribundum* , a hybrid of *A. androsaemifolium* and *A. cannabinum* , integrates parental traits through HHS, exhibiting morphological intermediates and fixed heterozygosity at allozyme loci (Johnson et al. 1998), with dynamic regulation further optimising these traits.

*Apocynum pictum* ‘Hongzhon’ (APZ), which is suspected to be a hybrid of *A. hendersonii* ‘Guazho’ (AHG) and *A. venetum* ‘Hongxia’ (AVX), combines the environmental tolerance of AHG with the high flavonoid yield of AVX, making it necessary to study hybrid viability and adaptation within the *Apocynum* genus. In hybrids, structural variations, such as chromosomal inversions, can maintain advantageous gene combinations by suppressing recombination, which is well‐documented in plant evolutionary studies and is likely pivotal in the adaptations observed in *Apocynum* (Yang et al. 2019). However, taxonomic disputes complicate this exploration. For example, in China, *A. venetum* (red hemp), *A. pictum* (white hemp), and *A. hendersonii* (large‐leaf white hemp) are variably classified as two or three species based on stem colour and leaf size (Jiang et al. 2021; Li et al. 2019). Morphologically, *A. pictum* and *A. hendersonii* are similar in leaf size and stem colour, while *A. venetum* is characterised by reddish stems and elevated flavonoid levels, with *A. pictum* displaying intermediate levels of flavonoid content and stress tolerance (Gao et al. 2019). This taxonomic ambiguity complicates the delimitation of species divergence and the evolutionary role of hybridization, thereby limiting the ability to unravel the genomic basis of trait integration in APZ. Nevertheless, the dynamic regulation, through the genomic stability of HHS and the expression flexibility of ASE, may integrate and optimise these traits in APZ under fluctuating conditions.

Despite being renowned for stress tolerance and flavonoids, systematic studies of genome evolution, flavonoid diversity, and adaptive mechanisms of *Apocynum* remain limited. Existing molecular data derived from partial markers only partially elucidate interspecific differences in *Apocynum* and fail to clarify the species divergence or the contribution of hybridization to trait evolution (Abubakar et al. 2022; Shao et al. 2022; Xu et al. 2021). Similarly, the evolutionary diversity of flavonoids and their stress‐mitigating functions lack comprehensive genomic insight. In hybrid progeny, ASE could optimise several traits, including stress resistance or flavonoid yield under varying conditions, which remain uncharted in *Apocynum*, particularly how APZ integrates the resilience of AHG and flavonoid traits of AVX. These gaps in the classification and genomic understanding hinder a thorough grasp of hybridization mechanisms and impede precision breeding, which has historically relied on selecting natural variants for resilience or flavonoid content without effectively combining both (Dorjee et al. 2024; Gao et al. 2019; Xu et al. 2021).

These challenges may be addressed by exploiting the telomere‐to‐telomere (T2T) genome sequences of APZ, AVX, and AHG. Therefore, this study assembled the T2T genomes of the three *Apocynum* species, in combination with population genetics and functional validation, to elucidate how hybridization integrates and optimises ecological adaptability and flavonoid traits in APZ and to establish a foundation for breeding advancements for the shrub. The objectives of the study included confirming the origin of APZ as a hybrid of AHG and AVX and clarifying taxonomic status through species divergence timelines and gene flow analysis to reveal the roles of structural variations such as inversions and ASE in aggregating salt‐stress tolerance and flavonoid traits and to validate the potential of the circadian rhythm *LHY* gene as a reproductive isolation factor while identifying breeding targets such as AVX‐derived *AvFLS* and *AvCHS* alleles. By integrating these genomes, this research aimed to bridge the gaps in *Apocynum* genome evolution and adaptability research and offer a genomic framework to accelerate the development of stress‐resilient and flavonoid‐rich cultivars capable of thriving in fluctuating saline‐alkaline environments.

## 2 Results

### 2.1 Phenotypic and T2T Genomic Studies of Three *Apocynum* Species

**FIGURE 1:**
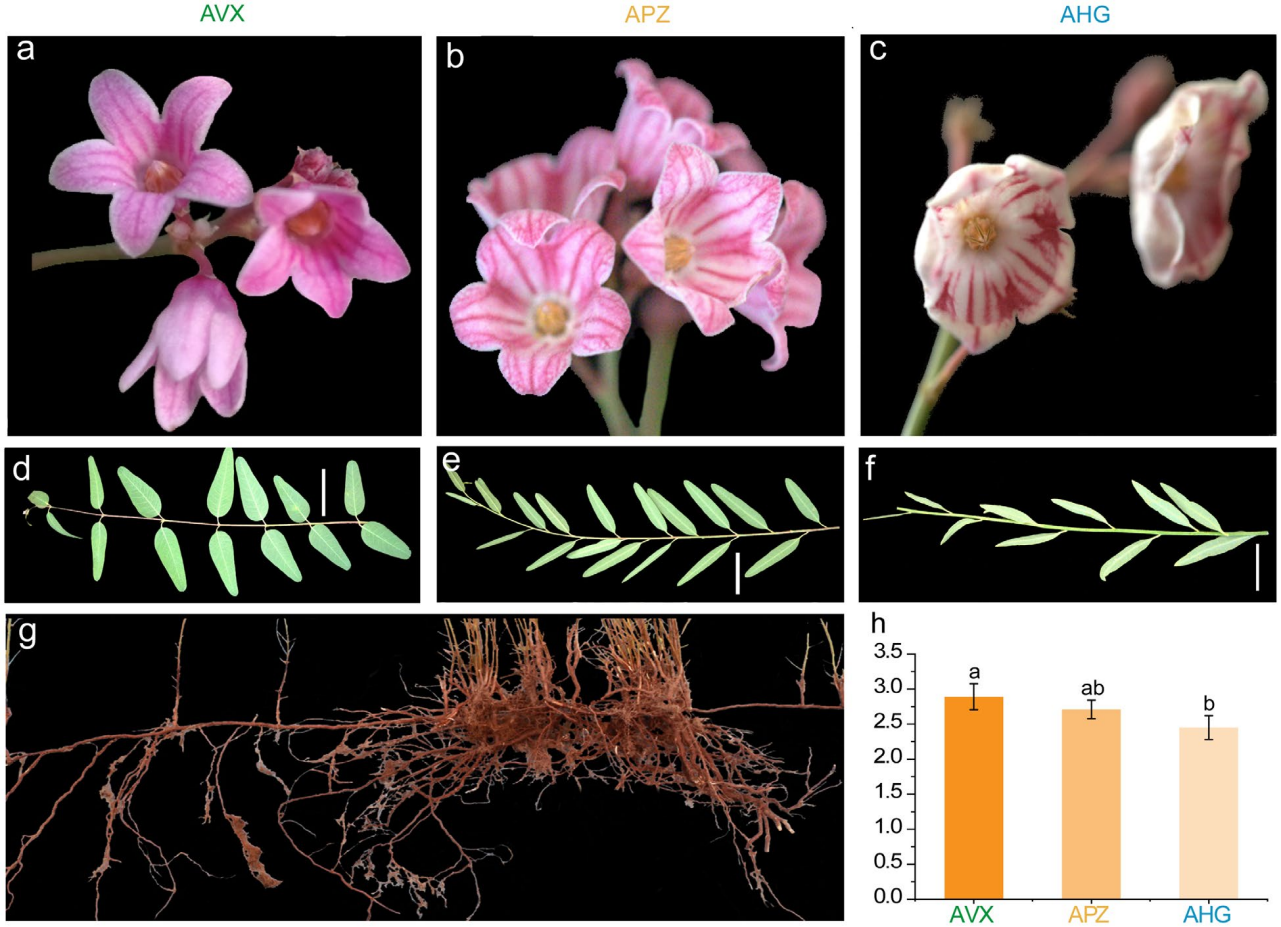
Morphological characterisation and flavonoid content of three *Apocynum* species. (a–c) Flower morphology of *Apocynum venetum* (AVX), *A. pictum* (APZ) and *A. hendersonii* (AHG), respectively. (d–f) Leaf morphology of AVX, APZ, and AHG, respectively, highlighting differences in their leaf shape and size. (g) Root morphology of *Apocynum*. (h) Total flavonoid content (g/100 g) in AVX, APZ, and AHG, with significant differences indicated by letters. Means followed by the same letters on the error bars are not statistically different (*p* > 0.05).

We evaluated the morphology and flavonoid content of three *Apocynum* species, including APZ, AHG, and AVX. The petal colour in AVX was red, white in AHG, and an intermediate pink with white stripes in APZ (Figure 1a–c). Leaf morphology showed species‐specific differences, with the leaves in AVX wider, narrower in APZ, and elongated and slender in AHG (Figure 1d–f). The root morphology of APZ showed a well‐developed root system (Figure 1g). Total flavonoid content in AVX (2.89 ± 0.17 g/100 g) was significantly higher than in AHG (2.45 ± 0.13 g/100 g, *p* < 0.05), while APZ (2.73 ± 0.15 g/100 g) showed an intermediate value, reflecting differences in flavonoid accumulation among the species (Figure 1h). These phenotypic differences suggested potential hybridization and trait variation, supporting further genomic investigation.

To establish a genomic framework, we assembled T2T genomes of APZ, AVX, and AHG by integrating PacBio HiFi sequences (44.1, 88.2, 61.9, Gb), Hi‐C sequences (61.2, 55.6, 182.4, Gb), and ONT ultra‐long reads (49.8 Gb for AHG; Tables S1 and S2). Hi‐C interaction matrices were used to validate the assembly of the 11 chromosomes for each *Apocynum* species, reflecting robust contig interactions (Figure S2). The resulting genome size was approximately 227.90 Mb with an N50 contig size of 20.96 Mb in APZ, 228.79 Mb with an N50 contig size of 20.59 Mb in AVX, and 222.39 Mb with an N50 contig size of 21.00 Mb in AHG, which is consistent with k‐mer estimates (Figure S1 and Tables S3–S6). The quality of the sequence assembly was high, with a BUSCO completeness exceeding 98.2%, while the consensus quality values were 49.1 in APZ, 42.8 in AVX, and 45.6 for AHG (Table S7).

**FIGURE 2:**
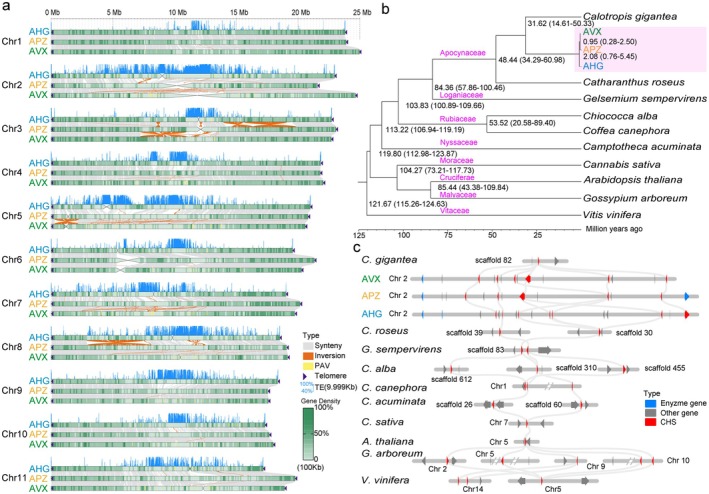
Telomere‐to‐telomere (T2T) genome of *Apocynum* species, Phylogenetic and evolution of three *Apocynum* species. (a) Chromosomal sequence synteny comparison and structural variations between three *Apocynum* T2T genome assemblies. (b) Phylogenetic relationships of three *Apocynum* species and related species. The estimated divergence time (million years ago, MYA) is indicated at each node, while family names are indicated in purple. (c) The tandem duplications of chalcone synthase in three *Apocynum* species and related species.

Telomeric repeat sequences (CCCTAAA/TTTAGGG) are present at the termini of all 11 chromosomes in each species (Figure 2a), while the centromeres that were identified de novo varied across species. The centromeric regions of AHG were located on eight chromosomes, while those for AVX and APZ were found on four chromosomes each. The lengths of the centromeres spanned between 76.45 and 4756.77 kb, with monomer repeats ranging from 110 to 886 bp (Table S8). Higher‐order repeat (HOR) analysis indicated that most centromeres consisted of single monomers, while some had multiple units (Figure S3). The AHG had the most comprehensive centromere annotation dominated by transposable elements (TEs) (88.46%), with satellite DNA (44.36%) and Gypsy‐type retrotransposons (39.66%) prevailing over minor *Copia‐*type elements (2.44%; Table S9). These results suggest that *Gypsy* retrotransposons disproportionately drive centromere evolution in *Apocynum*. Conserved motifs underscored the evolutionary continuity in the three *Apocynum* species. For instance, motifs on Chr7‐9 (prefix#circ18‐471) in AHG shared 91.79% identity with those in AVX in Chr8 (prefix#circ12‐455); AHG (prefix#circ9‐528) and AVX (prefix#circ3‐495) shared 90.80% of their conserved motifs on Chr6, while the AVX (prefix#circ9‐830) had a 100% similarity with APZ (prefix#circ13‐830) on Chr5 (Table S8).

The genomes of the three *Apocynum* species also consisted of repetitive sequences, with the long terminal repeats (LTRs) comprising 28.89%, 29.81%, and 29.18% of the APZ, AVX, and AHG genomes, respectively (Table S10). This modest repetitive fraction is lower than those in many angiosperms and likely contributes to their compact genomes. We annotated 24 528, 24 622, and 25 068 protein‐coding genes for APZ, AVX, and AHG respectively, with average gene lengths of 3160–3299 bp, coding sequences of 1186–1232 bp, and approximately 5 exons per gene (Table S11). The functional annotation assigned roles to 92.11%–94.59% of genes, with 99.0% of core conserved genes recovered (Tables S12 and S13). These high‐quality genomes provided a robust foundation for subsequent phylogenetic and hybrid origin analyses.

### 2.2 Phylogenetic and Evolutionary Insights Into Flavonoid Traits of Three *Apocynum* Species

Using the T2T assemblies, we explored phylogenetic relationships and evolutionary traits among APZ, AVX, AHG, and 10 other angiosperms, including *Vitis vinifera* and *Calotropis gigantea* . Gene family clustering identified 16 412 (APZ), 16 498 (AVX), and 16 370 (AHG) gene families, outnumbering those in the other species (Table S14), reflecting a conserved genomic architecture within *Apocynum*. Species‐specific gene families were scarce, with only 61 (APZ), 60 (AVX), and 88 (AHG) unique families, unlike the more extensive expansions in other taxa (Table S14). A maximum‐likelihood phylogeny, constructed from 896 single‐copy nuclear orthologs, placed *Apocynum* as sister to *C. gigantea* , diverging ~31.86 million years ago (Mya; Figure 2b). Within the genus, AVX and APZ clustered together, diverging ~0.95 Mya, while AHG separated earlier at ~2.08 Mya.

Evolutionary analysis uncovered lineage‐specific innovations in flavonoid biosynthesis critical to *Apocynum*'s ecological resilience. Gene family expansion analysis detected 291 (APZ), 268 (AVX), and 575 (AHG) expanded families, which are fewer than in related species and enriched in phenylpropanoid/flavonoid biosynthesis (e.g., *CHS* (chalcone synthase), *CHI* (chalcone isomerase), *LDOX* (leucoanthocyanidin dioxygenase)), monoterpenoid metabolism, and oxidative stress response pathways (Table S15; Figures S4 and S5). Notably, four flavonoid‐related and seven phenylpropanoid‐related families showed significant expansion, including *Apocynum*‐specific tandem duplications of *CHS* homologues (Figure 2c). These genomic features likely enhance flavonoid diversity and abiotic stress tolerance, particularly in *A. venetum*, consistent with prior observations (Fu et al. 2022; Shao et al. 2022).

### 2.3 APZ Was Formed by the Hybridization of AVX and AHG

**FIGURE 3:**
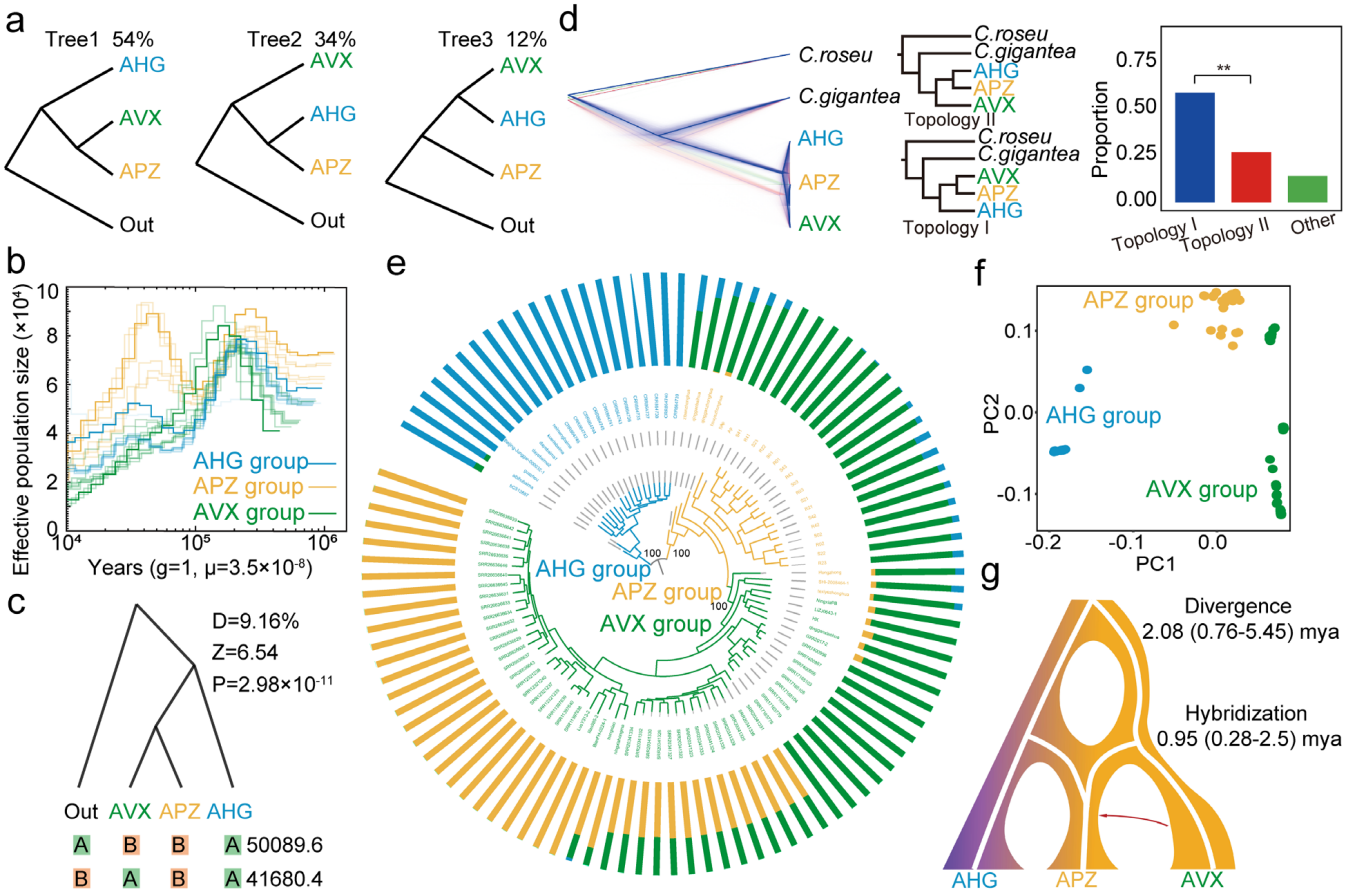
Genomic Evidence for APZ was formed by the hybridization of AVX and AHG. (a) Phylogenetic topologies are based on all resequenced individuals in which APZ has a close relationship with AVX (green) or AHG (blue). (b) Estimates of the effective population size over time are shown for AVX, APZ and AHG accessions in green, yellow and blue, respectively. (c) Four‐taxon ABBA/BABA test of introgression based on D statistics. The upper plot shows the phylogenetic relationships among the four species. (d) Phylogenetic topologies of 8443 single‐copy nuclear genes. Left: Gene trees of orthologous genes. Blue and red lines indicate two tree topologies in which APZ has a close relationship to AVX (I) or AHG (II). Right: Number of orthologous genes that exhibit phylogenetic topologies I or II. Statistical significance was determined by a one‐tailed binomial test. (e) Phylogeny and population structure of all samples based on analyses of nuclear SNPs for the 104 accessions. (f) The PCA score plot of the first two components for the 104 accessions. (g) Simplified schematic of the demographic scenario modelled by fastsimcoal2. The red arrow indicates asymmetric gene flow. * and ** indicate significance at the 0.05 and 0.01 levels, respectively.

To confirm the hybrid ancestry of APZ, a whole genome of 25 *Apocynum* accessions was sequenced and used to identify three genetic clusters via population structure and phylogenetic analyses including AVX‐, APZ‐ and AHG‐dominated groups in clade 1, 2, and 3, respectively (Figure S6 and Table S16). Admixture analysis with *K* = 3 corroborated this tripartite structure (Figure S7), while mapping rates to the APZ reference genome exceeded 98%, ensuring data reliability (Tables S17 and S18). Sliding‐window SNP phylogenies showed topological variability, with 54% clustering AHG as an outgroup to AVX and APZ, and 34% placing AVX as an outgroup to AHG and APZ (Figure 3a). PSMC analysis revealed distinct demographic histories, with all three populations peaking in effective population size (Ne) at ~110 000 years ago, with APZ and AHG showing a secondary peak, though AHG sharply declined (Figure 3b). Significant gene flow between AVX and APZ was evidenced by *D*‐statistics (*D* = 9.16%, *Z* = 6.54; Figure 3c) and their low genetic differentiation (*F*st = 0.08) compared to that between AHG and AVX (*F*st = 0.31) and between AHG and APZ (*F*st = 0.22) (Figure S8). APZ displayed higher heterozygosity than AVX and AHG (Figure S9), with a nucleotide diversity (*π*) of 1.31 × 10^−3^ in APZ surpassing that of AHG (1.13 × 10^−3^) and AVX (1.12 × 10^−3^). Linkage disequilibrium (LD) was also elevated in APZ relative to the other two species (Figure S10), suggesting that high heterozygosity sustained its genetic diversity. These results collectively indicate that hybridization enhances the genetic diversity of APZ and ties it more closely to AVX.

We assembled 24 chloroplast genomes (150 672–150 898 bp; GC content: 38.35%–38.38%) from resequencing data, each containing 86 protein‐coding genes, 37 tRNAs, and 8 rRNAs (Figure S11 and Table S19). Phylogenetic analyses of chloroplast sequences, coding regions, and SNPs consistently split accessions into two clades: Clade 1 (AVX) and Clade 2 (AHG and APZ; Figure S12). These clades were distinguished by 217 SNPs and 56 InDels, accounting for 62% and 69% of chloroplast variation, respectively (Table S20). Clade‐specific variations in non‐conserved regions of *ycf1* (5‐bp insertion) and *rpoC2* (8–9 bp deletions) were exclusive to Clade 1, serving as reliable molecular barcodes to differentiate AVX from AHG/APZ accessions (Figures S13 and S14; Table S21). Mitochondrial genomes, assembled as three circular contigs per species, showed strong synteny between APZ and AHG (Figures S15 and S16). Phylogenetic analysis of mitochondrial SNPs and InDels aligned with chloroplast results, and plastid‐nuclear integration confirmed AHG as the maternal plastid donor to APZ (Figure S17), consistent with an earlier AHG divergence.

Using *Catharanthus roseus* and *C. gigantea* as outgroups, we identified 8443 single‐copy orthologs across AHG, AVX, and APZ. Phylogenetic analysis revealed two main topologies, including 4968 orthologs that grouped APZ with AVX in Topology I (Figure 3b) and 2274 orthologs that paired APZ with AHG in Topology II (Figure 3d). PhyloNet analysis supported APZ's hybrid origin, with the 54% APZ‐AVX group matching SNP‐based phylogenies (Figure 3a). Analysis of transcriptomes and resequencing data from 104 accessions, including 20 AHG, 57 AVX, and 27 APZ (Table S22) resolved three subgroups via phylogeny and PCA, with APZ displaying mixed ancestry from AHG and AVX (Figure 3e,f). These findings confirm APZ as a hybrid of paternal AVX and maternal AHG, formed ~0.95 Mya, with a greater nuclear contribution from AVX (Figure 3g).

### 2.4 Structural Variations Preserve Adaptive Traits in *Apocynum* Species

**FIGURE 4:**
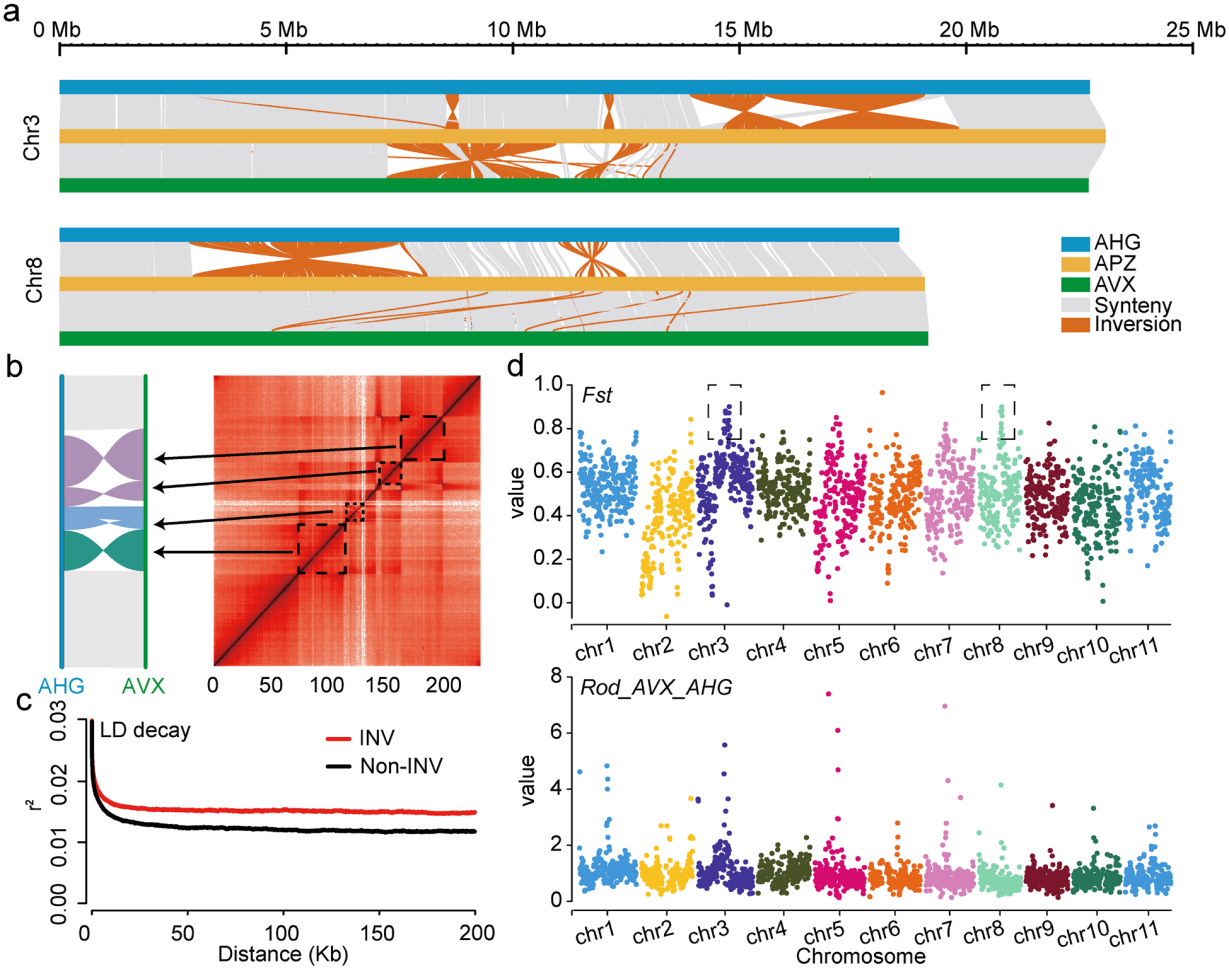
Structural variations and linkage disequilibrium (LD) in *Apocynum*. (a) The collinearity between AVX, APZ, and AHG on Chr3 and Chr8. (b) The AHG Hi‐C data mapped to the APX genome. The inversions between AHG and APZ on Chr3. (c) The LD between inversion and non‐inversion regions on Chr3 and Chr8. (d) The selective sweep analysis of INDELs in AVX and AHG populations.

To further explore the APZ's hybrid mechanisms, we aligned 26 *Apocynum* accessions, including individuals from AVX, AHG, and APZ populations to the APZ reference genome and identified 351 372 non‐redundant variants, comprising 268 551 SNPs (76.42%), 79 699 InDels (22.68%), and 3122 structural variants (SVs) (0.89%), and categorised the accessions as AVX‐homozygous, AHG‐homozygous, or heterozygous (Table S23). Heterozygous SVs and SNPs clustered within inversion regions in APZ individuals, reflecting hybridisation signatures (Figure S18). Collinearity analysis identified five large inversions in the APZ reference sample, including three on Chr3 (7.22–10.95 Mb, 11.35–13.04 Mb, 14.52–19.86 Mb) and two on Chr8 (2.91–8.02 Mb, 11.06–12.80 Mb; Figure 4a). Population‐level breakpoint analysis revealed that AVX and AHG populations are homozygous in these regions but differ in inversion states; APZ, having inherited one set of genomes from each parent, exhibits heterozygous inversions across its population (Figure S20; Tables S24–S26). Selective sweep analysis based on InDel variations between AVX and AHG populations detected significant selection signals within these inversion regions (Figure 4d), suggesting adaptive divergence that APZ retained through hybridisation. Haplotype assembly and collinearity profiles between AVX and APZ and between APZ and AHG, supported by Hi‐C data for the reference APZ sample, validated this heterozygous configuration in the individual (Figure 4b; Figures S19 and S23), with breakpoints confirmed by raw read mapping and IGV visualisation (Figures S21 and S22).

The LD within these inversion regions exceeded that in non‐inversion regions of Chr3 and Chr8 (Figure 4c), suggesting suppressed recombination due to slower LD decay in these regions, which also preserves parental haplotypes. Genotyping across APZ accessions showed variable heterozygosity for the Chr3 inversion that ranged from 7.22 to 10.95 Mb. Five samples of Hongzhong without breakpoints exhibited > 94% heterozygosity, while three with breakpoints of qingganbaihua displayed > 98% AVX‐like homozygosity, with consistent patterns in Chr3 (14.52–19.86 Mb) and Chr8 (2.91–8.02 Mb) inversions (Tables S24–S26). KEGG enrichment analysis of inversion regions identified associations with brassinosteroid biosynthesis, MAPK signalling, and plant hormone signal transduction (Figure S24), alongside three flavonoid biosynthesis genes of *CHI*, supporting their role in stress adaptation and flavonoid retention. Smaller SVs (< 1 Mb) were enriched in flavonoid biosynthesis and alkaloid pathways (Figure S25), further diversifying the adaptive traits in *Apocynum* accessions. These findings suggest that structural variations, particularly inversions, enhance APZ's stress resilience by maintaining AHG‐derived stress response genes, laying a foundation for breeding stress‐resistant, flavonoid‐rich cultivars, while flavonoid trait enhancement may rely on other genomic mechanisms.

### 2.5 ASE and HHS Optimise Stress and Flavonoid Traits

**FIGURE 5:**
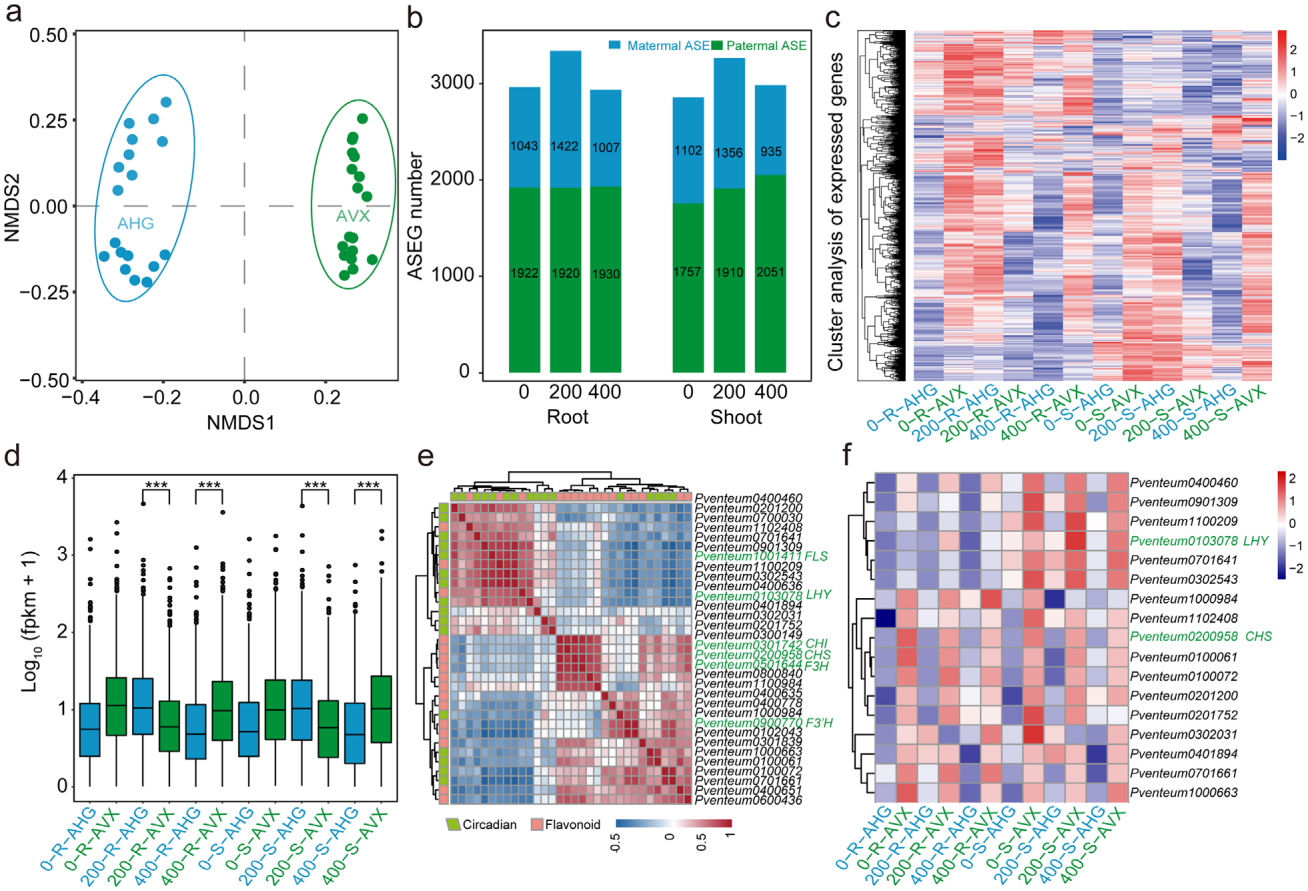
Summary and features of ASEGs in APZ. (a) Multiple‐dimensional scaling (MDS) analysis of expression profiles of alleles on shoots and roots under different salt stress. (b) Numbers of ASEGs in shoot and flag leaf under different salt stress. (c) Heat‐map of the expressed direction‐shifting ASEGs under different salt stress. (d) The allele expression levels of the direction‐shifting ASEGs under salt conditions in APZ, independent *t*‐test. ***p < 0.001. (e) Correlation heatmap of flavonoid and circadian gene expression in APZ under salt stress. (f) Heat‐map of the expressed ASE genes related to circadian rhythm under salt stress in APZ.

To elucidate how the APZ's hybrid origin enhances adaptive traits, we analysed ASE at loci that were homozygous in the parental species but heterozygous in APZ, where differential parental allele expression may underpin hybrid vigour. We employed two complementary approaches. This included SNP calling against the APZ reference genome, using transcriptome and resequenced data from AVX and AHG to distinguish parental alleles at heterozygous loci in APZ and RNA‐seq analysis of APZ shoots and roots under 0, 200, and 400 mM NaCl, followed by mapping of the reads to parental CDS sequences to quantify ASE. This approach identified 5268 ASE genes (ASEGs), with paternal (AVX‐biased) ASEGs significantly outnumbering maternal (AHG‐biased) ones (Figure 5b and Figure S26). The ASEGs included 972 with consistent bias across tissues and conditions, and 4296 with context‐dependent patterns, with 2774 ASEGs showing directional‐shifting bias and 1522 exhibiting non‐directional variability. Non‐metric multidimensional scaling (NMDS) validated haplotype‐specific read mapping, with AVX and AHG transcriptomes clustering distinctly (Figure 5a).

Functional enrichment analysis revealed ASE's critical role in dynamically optimising APZ's hybrid adaptation across varying salt‐stress conditions. Consistent ASEGs were enriched in flavonoid biosynthesis, glycerophospholipid metabolism, and Circadian rhythm—plant (Figure S27), while direction‐shifting ASEGs were linked to plant hormone signal transduction and MAPK signalling (Figure S28). Across all 5268 ASEGs, flavonoid biosynthesis, plant hormone signalling, Circadian rhythm—plant, and the MAPK pathway emerged as top enriched categories (Figure S29). Salt stress modulated ASE bias: under 200 mM NaCl, AHG‐biased genes were enriched in MAPK signalling and plant hormone signal transduction, as validated by RNA‐seq of AHG showing upregulated genes in these pathways (Figure S31), shifting to AVX dominance at 400 mM NaCl (Figure 5c,d). Differential expression analysis supported this pattern, with DEGs at 200 mM NaCl enriched in hormone and MAPK signalling, and at 400 mM NaCl in flavonoid biosynthesis (Figures S30 and S32). Key flavonoid biosynthesis genes, such as *AvFLS*, *AvCHS5*, and 17 plant circadian rhythm‐related genes, including *LHY*, showed a consistent AVX bias across all the salt‐stress conditions (Figure 5f). Interestingly, *CHS5* overlapped in both groups, while cluster analysis separated the 32 genes into two distinct modules, with *LHY* strongly correlating with *AvFLS* in the circadian module (Figure 5e), suggesting that *LHY* may coordinate flavonoid synthesis via circadian regulation.

After identifying the ASE‐driven genes critical to flavonoid synthesis and stress adaptation, we subsequently examined their evolutionary significance in the HHS of APZ. The HHS relies on the inheritance of alternate parental premating reproductive isolation (RI) genes, which are particularly effective between moderately diverged parents like AVX and AHG, enhancing hybrid stability and trait retention. In this framework, we identified 351 positively selected genes (PSGs), including four such as *CHS5* in flavonoid biosynthesis and three such as *LHY* in circadian rhythm (Table S27), underscoring their roles in the evolutionary stability and ecological adaptability of APZ. Resequencing revealed five nonsynonymous SNPs on the *LHY* gene (Table S28) that distinguish AVX and AHG, with APZ heterozygous at these loci, under strong positive selection (HKA *p* < 0.01), suggesting a potential role of the gene in regulating flowering time and reproductive isolation. This predominance of AVX‐biased ASEGs, spanning flavonoid and circadian pathways, suggests that APZ leverages AHG‐derived alleles for moderate stress resistance and AVX‐derived alleles for enhanced resilience under severe stress, amplifying its ecological adaptability.

### 2.6 Functional Validation of Flavonoid Biosynthesis Genes

**FIGURE 6:**
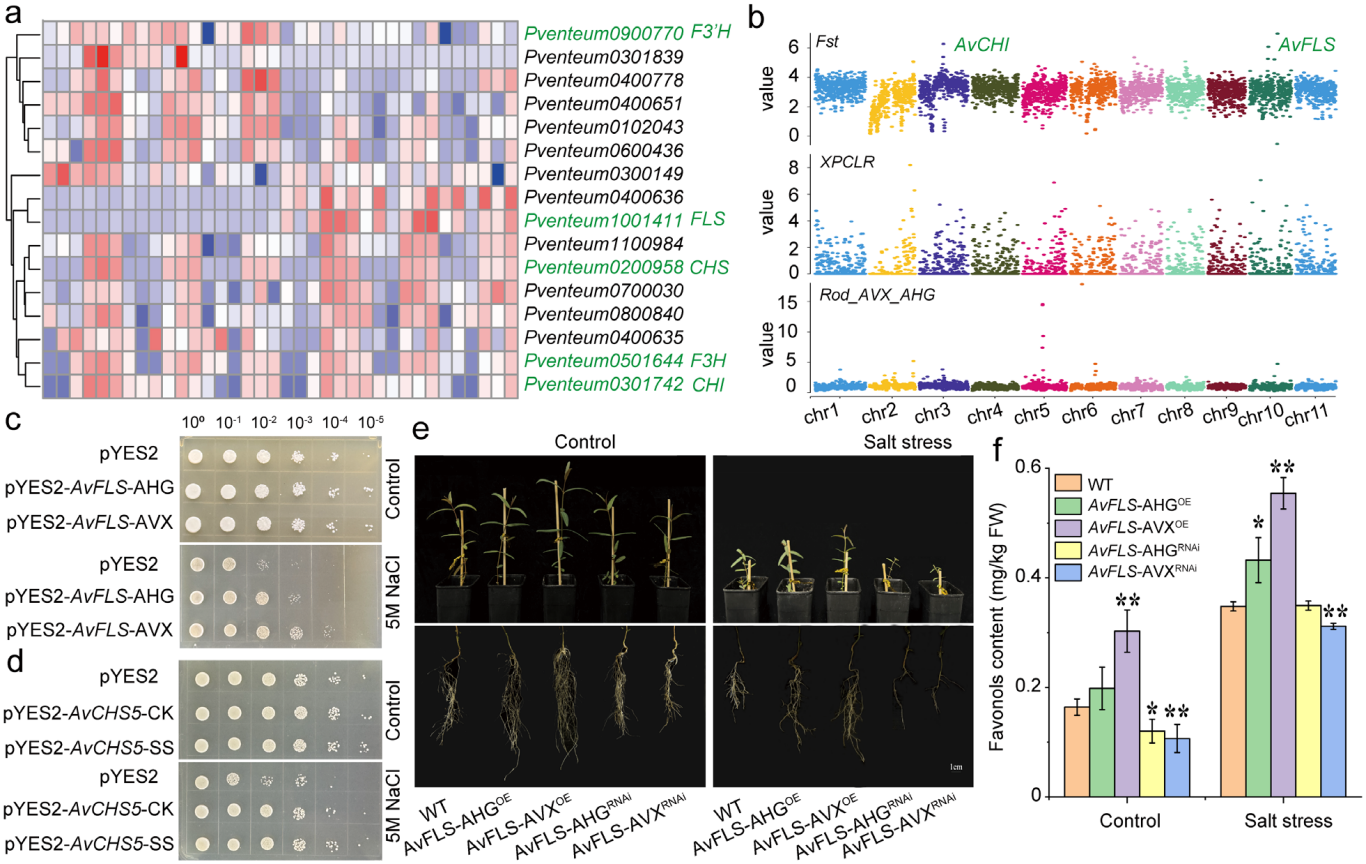
Functional divergence of alleles related to flavonoid accumulation and salt tolerance in APZ. (a) Heat‐map of the expressed allele‐specific expression genes related to flavonoid biosynthesis under salt stress in APZ. (b) Positively selected genes in flavonoid biosynthesis based on the comparison between AVX and AHG using *Fst*, *XP‐CLR*, and ROD metrics. (c) The transgenic analysis of the two genotypes of the *AvCHS*5 gene in yeast under salt stress. (d) The transgenic analysis of the two genotypes (AVX, AHG) of the *AvFLS* gene in yeast under salt stress. (e) Phenotypes analysis of different transgenic root hairs of two genotypes (AVX, AHG) of *AvFLS* under salt stress. The scale bar corresponds to 2 cm. The flavonol content (f) of *AvFLS* in different transgenic root hairs of *AvFLS* under salt stress. * and ** indicate significance at the 0.05 and 0.01 levels, respectively.

To validate the role of ASEGs in aggregating stress resistance and flavonoid‐mediated resilience in APZ, we assessed their functional contributions to salt tolerance and flavonoid accumulation by focusing on *AvFLS* and *AvCHS* alleles inherited from its hybrid parents, AVX and AHG. The APZ displayed a mid‐parent heterosis in flavonoid content that was skewed towards its paternal parent (AVX), suggesting the inheritance of elevated flavonoid levels from AVX (Abubakar et al. 2022; Gao et al. 2019). Subsequently, we reconstructed the flavonoid biosynthetic pathway for *Apocynum* species based on homologous *Arabidopsis* genes and prior studies (Liu, Feng, et al. 2021; Tohge et al. 2017), KEGG database and gene expression profiles under salt stress (Figure S33). The ASE analysis identified 16 flavonoid biosynthesis genes in APZ, including *AvCHS*, *AvCHI*, flavanone 3‐hydroxylase (*AvF3H*), flavanone 3′‐hydroxylase, and flavonol synthase (*AvFLS*), with consistent ASE patterns across tissues (Figure 6a).

Transgenic assays for *AvFLS* and yeast assays for *AvFLS* and *AvCHS5* were conducted with three replicates per experiment. The *AvCHS* gene family, which is expanded via tandem duplication, forms an *Apocynum*‐specific gene cluster absent in the 13 plant species used for comparison, where *CHS* homologues are dispersed (Figure 2d), likely resulting from post‐duplication divergence, with synteny retained in duplicated regions. Two genes in this cluster exhibited higher expression levels in APZ shoots and roots under salt stress compared to the other three *CHS* genes (Figure S34). The ASE analysis of *AvCHS5* at 400 mM NaCl revealed a paternal (AVX‐biased) heterozygous bias, accompanied by a fixed nonsynonymous mutation (Table S29 and Figure S35). To assess this variation, we expressed *AvCHS5* alleles in *Saccharomyces cerevisiae* using pYES2‐*AvCHS5*‐AVX and pYES2‐*AvCHS5*‐AHG vectors. Both variants significantly enhanced yeast salt tolerance without fitness trade‐offs (Figure 6d), suggesting that *AvCHS5* contributes to the salt adaptation of APZ through dosage effects rather than functional divergence, consistent with previous findings in rice (Liu et al. 2015).

To identify genes that contribute to flavonoid accumulation, we compared population genomic data between AVX and AHG and detected PSGs using *Fst*, *XP‐CLR*, and *ROD* metrics (Figure 6b). A 30% overlap was observed between *Fst* and *XP‐CLR* candidate regions, supporting the reliability of detected selection signals (Table S30). Interestingly, *AvFLS* emerged as a PSG that was previously linked to flavonoid accumulation (Turnbull et al. 2004). Five nonsynonymous mutations in the exon region of *AvFLS* could distinguish AVX and AHG individuals from all AVX and AHG individuals homozygous for these variants, while APZ individuals were heterozygous, bearing one AVX‐like allele and one AHG‐like allele (Table S31 and Figure S37). In vitro yeast experiments showed that the salt tolerance of the AVX allele of *AvFLS* was stronger than that of the AHG allele (Figure 6c). Therefore, to further explore the role of *AvFLS* in flavonoid accumulation and salt stress response, we constructed pYES2‐*AvFLS*‐AVX and pYES2‐*AvFLS*‐AHG vectors for the gene's overexpression, alongside RNAi constructs in AVX‐background hairy roots. The transcript levels of *AvFLS* (Figure S36) and flavonol content (Figure 6f) significantly increased in pYES2‐*AvFLS*‐AVX and pYES2‐*AvFLS*‐AHG overexpressing hairy roots but decreased in silenced roots, indicating that *AvFLS* regulates flavonol accumulation by modulating biosynthetic gene expression. Under salt stress, pYES2‐*AvFLS*‐AHG overexpressing hairy roots showed reduced plant height, root length, and root weight compared to pYES2‐*AvFLS*‐AVX counterparts, alongside higher H_2_O_2_ and MDA levels (Figure 6e and Figure S36), suggesting that the AVX allele enhances salt tolerance more effectively than the AHG allele. This validation integrates prior evolutionary insights into the *CHS* gene cluster and ASE findings, providing actionable breeding gene targets like *AvFLS* and *AvCHS* to develop cultivars with improved ecological resilience and therapeutic efficacy, and also bridging genomic insights to horticultural applications.

## 3 Discussion

### 3.1 Hybridization Drives Trait Integration in APZ: Genomic Insights

Our T2T genomic analyses revealed that hybridization drives superior trait aggregation in APZ, which is a hybrid of AHG (maternal) and AVX (paternal) formed at approximately 0.95 Mya after their divergence at ~2.08 Mya. Population structure analyses identified three distinct genetic clusters (Figure S6), while sliding‐window SNP phylogenies showed topological discordance (54% positioning AHG as the outgroup to AVX‐APZ, 34% positioning AVX as the outgroup; Figure 3a). Chloroplast and mitochondrial alignments grouped APZ with AHG (Figures S12 and S17), complementing nuclear genome analysis and confirming its hybrid origin. Significant gene flow between AVX and APZ (*D* = 9.16%, *Z* = 6.54; Figure 3c) and APZ's elevated heterozygosity (*π* = 1.31 × 10^−3^ vs. AHG's 1.13 × 10^−3^, AVX's 1.12 × 10^−3^; Figure S8) confirm its hybrid origin, suggesting that hybridization enhances genetic diversity and mitigates inbreeding depression, a benefit typical in young hybrids (Grant and Grant 1992). The maternal inheritance of AHG's plastids follows angiosperm patterns (Christie and Beekman 2017), while AVX's nuclear contributions, reflected in a low *Fst* (0.08 vs. AHG‐AVX 0.31; Figure S8), indicate a reticulate evolutionary history. This hybridization integrates the adaptability of AHG to saline‐alkaline conditions and the flavonoid richness in AVX, underpinning the ecological resilience of APZ in the Gobi desert. The *CHS* gene family expanded via tandem duplication across AVX, APZ, and AHG, and also underpins flavonoid diversity, contributing to stress resilience in APZ. The phylo‐ and population genomic analyses also support *A. venetum*, *A. pictum* , and *A. hendersonii* as three distinct *Apocynum* species in China (Jiang et al. 2021; Li et al. 2019) incongruent with their morphological differences. This genetic diversity of *Apocynum* species from hybridization set the stage for genomic innovations using structural variations and ASE to further refine the adaptive traits of APZ.

### 3.2 Genomic Innovations Supporting APZ Trait Integration

Building on this hybrid foundation, the adaptation of APZ emerged from a sophisticated interplay of structural and regulatory mechanisms. Large heterozygous inversions in APZ originated from homozygous inversion variants in AVX and homozygous non‐inversion variants in AHG (Figure 1D), thereby suppressing recombination, as evidenced by elevated LD (Figure 4c). These inversions, mapped with high‐resolution T2T sequence, preserve co‐adapted parental haplotypes, potentially fostering reproductive isolation and stabilising beneficial gene combinations, a mechanism well‐established in plant evolution (Dvorak et al. 2018; Xiao et al. 2025; Yang et al. 2019). Selective sweep analyses identified strong selection signatures within these inversions (Figure 4d and Figure 6b), enriching genes tied to stress response and some flavonoid biosynthesis components such as *CHI*. Genotyping across APZ accessions shows variable heterozygosity (Tables S24–S26), indicating that the inversions maintain AHG‐derived stress response loci and AVX‐inherited flavonoid potential. Smaller SVs < 1 Mb also subtly enhance flavonoid biosynthesis pathways (Figure S25).

Complementing these structural features, ASE dynamically adjusts gene expression to enhance adaptability across varying salt‐stress conditions. Across 18 APZ single plants, ASE exhibits a stress‐dependent shift: under 200 mM NaCl, AHG‐biased genes predominate, enriching stress signalling pathways such as MAPK and hormone transduction, while at 400 mM NaCl, AVX‐biased genes enhance stress signalling (e.g., MAPK) and maintain flavonoid biosynthesis (e.g., *AvFLS*, *AvCHS5*; Figure 4c,d). This shift enables selective expression of beneficial alleles, boosting hybrid vigour, as observed in hybrid rice (Shao et al. 2019). Similarly, APZ's ASE patterns reflect pronounced allelic dominance, resembling dominant allele effects in cassava, *S. glauca* and papaya hybrids under stress (Cheng et al. 2023; Liu, Chen, et al. 2021; Xu et al. 2023), suggesting conserved regulatory mechanisms with APZ‐specific flavonoid emphasis under high salt stress. Transcriptomic analysis of AHG further revealed that, under 200 mM NaCl, genes upregulated in MAPK and plant hormone signalling pathways (Figure S31) support stress resilience, complementary to the AHG‐biased contribution observed in APZ. Differential expression analysis of APZ's overall transcriptome supports this pattern, with DEGs under 200 mM NaCl enriched in hormone and MAPK signalling and under 400 mM NaCl in flavonoid biosynthesis (Figures S30 and S32). Functional validation highlights *AvFLS*'s pivotal role: its AVX allele, marked by five nonsynonymous mutations (Figure S37 and Table S31), boosts flavonol accumulation and salt tolerance in transgenic assays (Figure 6c,e), likely via ROS scavenging, aligning with its AVX‐biased expression (Xu et al. 2021). In contrast, *AvCHS5*, expanded by tandem duplication (Figure 2c), sustains high expression under stress, with yeast assays linking salt tolerance to copy number effects (Figure 6d) (Liu et al. 2015; Yu et al. 2024).

Additionally, 17 circadian genes (e.g., *LHY*) show AVX bias (Figure 5f). In APZ, *LHY* carries five nonsynonymous SNPs distinguishing AVX and AHG populations, remaining heterozygous and under strong positive selection as identified by our HHS analysis (HKA*P* < 0.01; Tables S27 and S28), consistent with its role in homoploid hybrid speciation studies like in the genus *Ostryopsis* (Wang et al. 2021). Correlation analysis of 32 genes based on flavonoid‐ and circadian‐related allele expression revealed that *FLS* strongly correlates with circadian module genes, while *CHS5*, as a shared allele, significantly associates with flavonoid modules (Figure 5e), suggesting its role in coordinating metabolic rhythms and flavonoid synthesis, consistent with *Arabidopsis* findings (Harmer et al. 2000). HHS identified 351 PSGs (e.g., *AvCHS5*, *LHY*; Table S27), indicating genome‐wide selection supports HHS and potentially drives reproductive isolation via the Dobzhansky‐Muller model (Bomblies and Weigel 2007; Wang et al. 2021). These *LHY* SNPs may regulate flowering time to reinforce isolation while enhancing flavonoid‐mediated resilience, with their stress adaptation role pending confirmation via transgenic assays. As *LHY* and *AvCHS5* lie outside inversions, HHS complements inversion‐locked alleles with broader genomic selection, enhancing APZ's adaptability.

Inversions safeguard haplotypes, ASE optimises expression through dynamic regulation, while HHS reinforces stability, enabling a modular regulation that balances stress tolerance with metabolic flexibility (Huang et al. 2016; Liu et al. 2024; Liu, Zhang, He, et al. 2022; Wang et al. 2022; Zhang et al. 2020). The synergy of the ASE dynamics and inversions in APZ challenges the traditional view that hybrid vigour primarily stems from genetic diversity, highlighting the evolutionary advantage of condition‐specific expression under fluctuating environments. This hybrid strategy surpasses the constitutive stress responses of many halophytes, including *Thellungiella salsuginea* , thereby driving the ecological success of APZ in the Gobi Desert with fluctuating saline‐alkaline conditions and also offering a novel model for understanding plant adaptation to extreme environments (Chen et al. 2015; Thevs et al. 2012).

### 3.3 Genomic Insights Guides Breeding and Beyond

APZ's exceptional adaptability and flavonoid production stem from natural hybridization between species and prolonged evolutionary adaptation that has spanned millions of years. Through T2T genome assembly, which provides unparalleled resolution and functional validation, this current research has elucidated the molecular mechanisms, such as inversions and ASE, underpinning these traits and offering critical scientific insights that transcend mere observation of the outcomes of nature. This current study uniquely demonstrates how the APZ heterozygous genome integrates the environmental resilience of AHG with the AVX flavonoid production, enabling targeted breeding strategies that bypass the slow pace of natural selection. AVX‐derived *AvFLS* and *AvCHS* alleles enhance salt tolerance and flavonol content, while AHG‐derived stress loci are preserved via inversions. These candidate genes (e.g., *AvFLS*, *AvCHS5*) may serve as targets in marker‐assisted selection and CRISPR editing of ASE regulatory regions (Sander and Joung 2014). The genome's low repetitive content (28.89%; Table S10) may also aid in locus mapping, while ASE dynamic regulation offers a versatile trait optimization strategy.

Despite these advances, our study underexplored the function of *AvCHI* and *AvF3H* genes, while the ecological roles of inversions and ASE require field validation. Future saline‐alkaline field trials could also confirm the performance of APZ, while analysis of additional genes and dissection of the ASE regulation via epigenetic modifications or cis/trans factors could refine trait tuning (Bao et al. 2019; Kakoulidou and Johannes 2024; Xue et al. 2025). Functional validation of *LHY* using transgenic or knockout approaches could also clarify its influence on flavonoid biosynthesis and hybrid stability (Srikanth and Schmid 2011), and enhance breeding targets for metabolic and reproductive traits. Single‐cell transcriptomics may reveal tissue‐specific ASE contributions and deepen insights into hybrid vigour. These advances will also enhance *Apocynum* breeding for resilience and yield in harsh environments, thereby offering a model for other stress‐tolerant crops like rice or sorghum. APZ retains its integrated traits through hybridization, enabling us to harness its dynamic regulation in breeding programmes and to rapidly develop cultivars that surpass their parental species in adaptability and yield, and also address global agricultural challenges with genomic precision. This research study not only provides a dynamic regulatory model for genomics and evolutionary biology but also informs ecology by revealing population‐level adaptation to fluctuating salinity and plant physiology through enhanced ROS scavenging via flavonoid metabolism, advancing cross‐disciplinary studies in extreme environments.

## 4 Materials and Methods

To investigate how hybridization aggregates adaptive traits in *Apocynum* and identify breeding targets, we employed telomere‐to‐telomere (T2T) genome assemblies to trace APZ's hybrid origin, integrated structural variation and allele‐specific expression (ASE) analyses to elucidate trait retention and optimization mechanisms, and conducted functional validations to confirm key gene contributions. This section outlines the experimental and analytical approaches used across these steps.

### 4.1 Plant Materials, Library Construction and Sequencing

*Apocynum venetum* ‘Hongxia’ (AVX), *Apocynum pictum* ‘Hongzhon’ (APZ) and *Apocynum hendersonii* ‘Guazho’ (AHG) were cultivated in experimental fields in Yuzhong, Gansu, China. High‐molecular‐weight genomic DNA was extracted from fresh leaves using the cetyltrimethylammonium bromide (CTAB) method. For long‐read sequencing, libraries with insert sizes of 15 and 100 kb were constructed for PacBio Sequel II HiFi and Oxford Nanopore Technologies (ONT) PromethION platforms, respectively. AHG was sequenced with one HiFi cell and one ONT flow cell, while AVX and APZ each utilised one HiFi cell. Illumina paired‐end libraries, prepared from 1.5 μg DNA, were sequenced on the HiSeq 2500 platform, yielding 150 bp reads. Hi‐C libraries were generated from young leaves digested with MboI (a 4‐cutter restriction enzyme) and sequenced on the HiSeq 2500 platform (150 bp paired‐end reads). PacBio HiFi sequencing yielded 44.1 Gb (AVX, ~193×), 88.2 Gb (APZ, ~385×), and 61.9 Gb (AHG, ~278×). ONT ultra‐long reads for AHG provided 49.8 Gb (~224×). Hi‐C sequencing generated 61.2 Gb (AVX, ~268×), 55.6 Gb (APZ, ~243×), and 182.4 Gb (AHG, ~820×). Publicly available RNA‐seq data for *A. venetum* and *A. hendersonii* (accessions: CRR864735‐CRR864746, SRR11397638‐SRR11397640, SRR12321237‐SRR12321240, SRR17163778‐SRR17163780, SRR17165103‐SRR17165105, SRR20341322‐SRR20341336, SRR26636629‐SRR26636646, SRR7400955‐SRR7400957) were retrieved from the National Genomics Data Center (Beijing Institute of Genomics, Chinese Academy of Sciences). To investigate allele‐specific expression (ASE) and differential gene expression under salt stress, we conducted mRNA sequencing of APZ shoots and roots under 0 mM, 200 mM, and 400 mM NaCl conditions. Libraries were constructed from 1 μg RNA per sample (18 samples total) using the TruSeq RNA Library Preparation Kit (Illumina, USA) and sequenced on the Illumina HiSeq 2500 platform, producing 150 bp paired‐end reads.

### 4.2 Genome Size and Heterozygosity Estimation

The three plants genome size was estimated using k‐mer frequency distribution generated from Illumina short reads. The program jellyfish (version 2.2.9) (Marçais and Kingsford 2011) was used to calculate 17‐mer frequency distribution, and then GenomeScope (http://qb.cshl.edu/genomescope/) was used to estimate genome size and heterozygosity based on the k‐mer frequency with default parameters.

### 4.3 T2T Genome Assembly and Quality Assessments

ONT ultra‐long reads were generated for AHG, while AVX and APZ relied on PacBio HiFi and Hi‐C data for assembly. For AHG, a combined assembly approach integrated PacBio HiFi and ONT ultra‐long reads using Hifiasm (Cheng et al. 2021). Contigs were polished iteratively with BGI short reads and PacBio long reads via NextPolish2 (Hu, Wang, Liang, et al. 2024). Hi‐C data were aligned to polished contigs using BWA v0.7.10‐r789 (Li and Durbin 2009) or Bowtie2 v2.3.2 (Langmead and Salzberg 2012) (“–end‐to‐end–very‐sensitive ‐L 30”), retaining valid read pairs (mapping quality) filtered by HiC‐Pro v2.8.1 (Servant et al. 2015). Chromosome‐level scaffolding was performed with 3D‐DNA (Dudchenko et al. 2017). ONT reads were error‐corrected with NextDenovo (Hu, Wang, Sun, et al. 2024) and gap‐filled using quarTeT (Lin et al. 2023). AVX and APZ genomes were assembled similarly using Hifiasm, followed by Hi‐C scaffolding and quarTeT gap‐filling.

The quality and completeness of the genome assembly were assessed from three aspects. First, we evaluated the mapping rates of the clean raw reads from transcriptomes and genomic DNA by TopHat2 (Kim et al. 2013) and BWA‐MEM with default parameters, respectively. Second, the completeness of the assembled genome was assessed by BUSCO (Simão et al. 2015), with the library of *embryophyta*_odb10. Third, the consensus quality value (QV) and error rate were calculated using Merqury (Rhie et al. 2020).

Centromere positions were initially inferred using StainedGlass (Vollger et al. 2022) to generate sliding‐box similarity heatmaps. KMC (Kokot et al. 2017) identified genome‐wide k‐mers, followed by microsatellite detection using SRF (Zhang et al. 2023). Centromere‐specific motifs were validated by aligning SRF sequences and analysed with stringdecomposer_1.1.2 (Dvorkina et al. 2020). Final centromere coordinates were determined by integrating these results, and higher‐order structures were resolved using HiCAT (Gao et al. 2023). To identify the telomeres, plant telomere sequences (TTTAGGG/CCCTAAA) were used to search the 100‐kb regions at both ends of the chromosomes using quarTeT.

### 4.4 Genome Annotation

Transposable elements (TEs) were predicted using both homology and de novo‐based approaches. Repeat sequences in the assemblies were identified using the Extensive de novo TE Annotator (EDTA, v1.8.5) (Ou et al. 2019). LTR retrotransposons were predicted using LTR_Finder (v1.07) (Xu and Wang 2007) and LTR_retriever (v2.6) (Ou and Jiang 2018). TIR transposons were identified using an integrated strategy with Generic Repeat Finder (v1.0) (Shi and Liang 2019) and TIR‐Learner (v1.19) (Su et al. 2019). Helitron transposons were detected using HelitronScanner (v1.1) (Xiong et al. 2014). LINEs were identified using RepeatModeler v2.0.1 (https://github.com/Dfam‐consortium/RepeatModeler). All programs were run with default parameters. The curated TE library (rice 6.9.5.liban) from EDTA was used to annotate repeat sequences with the parameters “–species others–step all–sensitive 1–evaluate 1–anno 1”.

Gene structures were annotated through an integrative approach combining three strategies: transcriptomic evidence from RNA‐seq data of AVX, APZ, and AHG tissues (root, leaf, stem), homology‐based predictions using protein sequences from seven related species ( *Calotropis gigantea* , *Camptotheca acuminata* , *Cannabis sativa* , *Catharanthus roseus* , *Chiococca alba* , *Coffea arabica* and *Gelsemium sempervirens* ), and ab initio predictions from six tools including Augustus (v.2.4) (Stanke et al. 2008), Genscan (v.3.1) (Burge and Karlin 1997), GeneID (v.1.4) (Alioto et al. 2018), GlimmerHMM (v.1.2) (Majoros et al. 2004), GeneMarkS‐T (Tang et al. 2015) and SNAP (v.2006‐07‐28) (Korf 2004), yielding non‐redundant gene sets of APZ, AVX, and AHG protein‐coding genes with EVidenceModeler (Haas et al. 2008).

### 4.5 Chloroplast and Mitogenome Genome Assembly and Annotation

Chloroplast genomes were assembled from Illumina short reads using GetOrganelle v1.7.1 (Jin et al. 2020), annotated via GeSeq (Tillich et al. 2017), and manually curated before visualisation with OGDraw (Lohse et al. 2007). Mitochondrial genomes were assembled from HiFi reads using Minimap2 v2.1 (Li 2018) and Canu (Nurk et al. 2020), followed by error correction and trimming. Annotation was performed analogously to chloroplast genomes.

### 4.6 Gene Family and Phylogenetic Analysis

OrthoFinder (v2.3.3) (Emms and Kelly 2019) software was used to cluster paralogous and orthologous groups from genomes of AHG, AVX, APZ, *C. gigantea* , *C. acuminata* , *C. sativa* , *C. roseus* , *C. alba* , *C. arabica* , and *G. sempervirens* . Single‐copy orthologs were aligned with MUSCLE v3.8.31 (Edgar 2004), filtered with Gblocks to retain conserved regions, and concatenated into a supermatrix. A maximum‐likelihood phylogeny was reconstructed using RAxML v8.2.12 (Stamatakis 2014), with parameter 100 bootstrap, rooted with FigTree v1.4.3 (http://tree.bio.ed.ac.uk/software/figtree/). Divergence times were estimated using Codeml and MCMCtree (Yang 2007) with TimeTree‐calibrated nodes. Gene family expansions/contractions were analysed with CAFE v4.2.1 (De Bie et al. 2006), and functional enrichment (GO/KEGG) was assessed using clusterProfiler (Yu et al. 2012).

### 4.7 Resequencing and Variant Calling

DNA was extracted from fresh leaves using a routine protocol. Resequencing paired‐end reads were generated using Illumina HiSeq 4000. For AVX, APZ, and AHG accessions, Illumina paired‐end sequencing achieved ~30× coverage per sample (Table S18). The raw data were first filtered by fastp (v0.12.6) (Chen et al. 2018); clean paired‐end reads of each accession were then aligned to the updated reference genome of APZ, including the nuclear and plastid genomes, using BWA with default parameters. The alignment files were sorted and indexed with SAMtools (Li et al. 2009). Variant calling was performed using GATK‐4.0 (McKenna et al. 2010) with default parameters, and variants were filtered by VCFtools (Danecek et al. 2011). An integrated pipeline was used in calling and filtering whole‐genome variants (SNPs, InDels). The variants with MAF greater than 0.05 and a missing rate less than 20% were further filtered. All variants were annotated by SnpEff (v3.652) (Cingolani et al. 2012).

### 4.8 Phylogenetic, Population and Ancestry Analyses

Maximum‐likelihood (ML) phylogenetic trees were reconstructed from multi‐consensus SNP sequences using FastTree (Price et al. 2009) with the ultrafast bootstrap method. Trees were visualised and annotated in iTOL (Letunic and Bork 2019). Population structure was assessed using ADMIXTURE (Alexander et al. 2009) with *K* values ranging from 1 to 10 and principal component analysis (PCA) via the smartpca function in (Patterson et al. 2006). At *K* = 3, SNP allele frequencies were tabulated to reflect subgroup stratification. PSMC++ (Terhorst et al. 2017) inferred historical effective population size (*Ne*) trajectories for AVX, APZ, and AHG, with parameters set to a mutation rate of 6.5 × 10^−9^ per synonymous site per generation, time boundaries of 200–25 000 generations. Genome‐wide ancestry of APZ was resolved using 2‐kb sliding windows (1‐kb step) of the SNP. For each window, ADMIXTURE for *K* = 3 and neighbour‐joining phylogenies (PHYLIP v3.695; 100 bootstraps) (Baum 1989) were applied to 26 resequenced individuals, with *Calotropis gigantea* as the outgroup. Twisst categorised topologies to quantify parental contributions (AVX vs. AHG), and topology weighting by iterative subsampling determined lineage‐specific ancestry weights (van Belleghem et al. 2017). To further investigate the presence of gene flow between parental lineages and the timing of species hybridization, we employed the coalescence‐based method implemented in fastsimcoal2 (Li et al. 2013). Single nucleotide polymorphisms (SNPs) were extracted from population data. To minimise potential biases in determining the ancestral allelic state, we generated a folded multidimensional site frequency spectrum. Alternative models of historical events, including radiation, introgression, and hybridization, were fitted to the joint site frequency spectrum. All parameter estimates were obtained through 100 000 simulations per likelihood estimation (‐n100 000, ‐N100 000) and 40 cycles of the likelihood maximisation algorithm. For each model, we conducted 50 independent runs and obtained their corresponding maximum likelihood values. The model with the smallest Akaike information criterion value was determined as the best.

### 4.9 Genome‐Wide Selective Sweep Analysis

To identify candidate genomic regions potentially affected by selections, nucleotide diversity (*π*) and population fixation statistics (*F*st) were calculated using VCFtools in a 20‐kb sliding window with a step size of 5‐kb. With a false discovery rate (FDR) < 0.05, *π* and *F*st were used for estimating the degree of pairwise genomic differentiation on candidate genes between pairs of subpopulations. To comprehensively identify potential selective regions, a reciprocal XP‐CLR (Racimo 2016) test was also performed between two compared populations. The reduction of diversity (ROD) values were calculated based on the ratio of *π* for a subpopulation with respect to a control subpopulation. All the output results of ROD and *Fst* were standardised and transformed into *z*‐scores using a 20‐kb sliding window with a 5‐kb step size. Candidate genomic regions were selected based on the top 1% values. We selected all the samples from each species to measure and compare patterns of linkage disequilibrium (LD). Haploview (Barrett 2009) was used to calculate the correlation coefficient *γ* ^2^ of pairwise SNPs with the parameters: ‐dprime ‐maxdistance 50 ‐minMAF 0.05 ‐hwcutoff 0.001 ‐minGeno 0.6.

### 4.10 Phylogeny Analysis of AVX, APZ, and AHG Accessions Based on Chloroplast and Mitogenome Genome Sequence

*Calotropis procera* (NC_041440.1) chloroplast genome and the genome NGS data of *C. gigantea* (PRJNA400797) were downloaded from the NCBI database, which was used as an outgroup. All chloroplast genomes, chloroplast single copy genes, or all chloroplast coding gene SNPs were aligned using clustalw with the ‐auto option, respectively. IQ‐TREE (version 1.6.3) (Minh et al. 2020) was used to reconstruct the maximum likelihood phylogenetic tree with 1000 replicates of ultrafast bootstrap. For all chloroplast genome sequences, alignment was performed using Cactus (Armstrong et al. 2020) with the ‐auto option, and conserved blocks of the alignment were used to reconstruct the phylogenetic tree with IQ‐TREE.

### 4.11 Identification and Validation of Structural Variants (SVs)

Large SVs (> 1 Mb) were identified by pairwise alignment of T2T chromosome‐level genomes (AVX, APZ, AHG) using MUMmer v4.0.0 (Marçais et al. 2018). Alignments shorter than 1000 bp were filtered with delta‐filter, and coordinates were extracted via show‐coords. Putative inversions (> 1 Mb) and breakpoint positions were called using SyRI (v1.5.6) (Goel et al. 2019). Homozygous inversions were distinguished from heterozygous inversions based on haplotype assembly consistency: homozygous inversions showed uniform haplotype orientation, while heterozygous inversions exhibited discordant orientations matching inversion breakpoints. Breakpoints were further validated by aligning population resequencing data to the APZ reference genome and visualising with Integrative Genomics Viewer (IGV).

For SVs < 1 Mb, Illumina‐based detection utilised Manta v1.6.0 (Chen et al. 2016) with default parameters. HiFi‐based SV calling involved read alignment with NGMLR v0.2.7 (Sedlazeck et al. 2018) followed by SV identification using Sniffles (v2.0) (Smolka et al. 2024). All SVs were annotated with bedtools (v2.30.0) (Quinlan and Hall 2010), including genomic feature overlaps and functional impact predictions.

### 4.12 Identification of Genes Showing Allele‐Specific Expression (ASEGs) in APZ and Homoploid Hybrid Speciation (HHS) Detection

Allele‐specific expression (ASE) analysis in APZ was performed using two complementary approaches. First, variants were called from genome resequencing and transcriptome data (20 AHG, 57 AVX, 27 APZ; Table S22) aligned to the APZ reference genome using GATK v3.8.0. SNPs were filtered with VCFTools (v0.1.14) (read depth > 8×) and annotated via SnpEff to prioritise coding regions. ASE loci were required to meet four criteria: (a) fixed homozygous discordance between AVX and AHG populations; (b) > 80% genotype consistency within parental populations; (c) APZ was genotyped based on SNP loci specific to AVX and AHG, retaining loci where homozygous samples (based on transcriptome and resequencing data) exceeded 80%, and at least three APZ samples were heterozygous; (d) SNP loci used to genotype the same gene spanned at least one exon, and different SNPs of the same gene were not in opposing directions in APZ. Second, a bidirectional comparison of CDS sequences annotated for AHG and AVX was performed to select optimal alleles. Using the CDS sequences of the optimal alleles as a reference, transcriptome data were quantified using Salmon (Patro et al. 2017). Alleles were considered ASE if they exhibited a log2 fold change > 1 or < −1 and a *p*adj < 0.05, as analysed by EdgeR (Robinson et al. 2010). Genes meeting these criteria were classified as ASEGs. Consistent ASE and direction‐shifting ASE were defined and identified following the methods of rice (Shao et al. 2019). Functional enrichment of ASEGs utilised clusterProfiler with GO terms (InterProScan‐annotated) and KEGG pathways (KofamScan‐derived), applying a *q*‐value cutoff of 0.05.

To investigate APZ's hybrid speciation from AHG and AVX and identify positively selected genes, we adapted methods from homoploid hybrid speciation studies (e.g., *Ostryopsis*) (Wang et al. 2021). Using filtered SNP data from 104 individuals (20 AHG, 57 AVX, 27 APZ), we conducted Hudson‐Kreitman‐Aguadé (HKA) tests to detect PSGs. The analysis compared APZ + AVX versus AHG, highlighting AVX's contribution to flavonoid‐mediated resilience and circadian rhythm regulation. For each gene, we counted polymorphic sites (SNPs) within the APZ + AVX group and fixed differences (SNPs with *F*st > 0.95) between APZ + AVX and AHG, using a custom script. Genes with HKA *p*‐values < 0.01 were retained. Fixed non‐synonymous mutations were quantified, selecting genes in the top 2.5% of fixed sites. PSGs were finalised based on three criteria: significant HKA *p*‐values, high fixed mutation counts, and confirmation of hybrid inheritance via allele‐specific validation, where APZ alleles aligned predominantly with AVX while showing introgression from AHG.

### 4.13 Functional Validation of Allelic Differentiation in Yeast and Transgenic Plants

Full‐length coding sequences of *AvFLS* alleles were amplified from AVX (paternal) and AHG (maternal) cDNA using allele‐specific primers and cloned into the yeast expression vector pYES2 (Invitrogen), generating pYES2‐*AvFLS*_AVX and pYES2‐*AvFLS*_AHG. *AvCHS5* alleles were amplified from APZ cDNA under control (AVX‐like, control allele under 0 mM NaCl) and salt‐stress (AHG‐like, salt‐stress responsive allele under 400 mM NaCl) conditions, followed by cloning into pYES2 to create pYES2‐*AvCHS5*_AVX and pYES2‐*AvCHS5*_AHG. Positive clones were confirmed by colony PCR with vector‐specific primers (pYES2‐F/R) and Sanger sequencing. Transformed *Saccharomyces cerevisiae* (strain BY4741) were cultured in synthetic dropout medium (SD/‐Ura) and subjected to salt stress by supplementing 5 M NaCl (Zhang et al. 2021). Transgenic assays for *AvFLS* and yeast assays for *AvFLS* and *AvCHS5* were conducted with three replicates per experiment. For functional validation in planta, *AvFLS* alleles were cloned into the pH7WGF2‐RR overexpression vector and RNAi constructs (RNAi‐*AvFLS*_AHG/AVX) using Gateway recombination (Invitrogen). These constructs were introduced into *Agrobacterium rhizogenes* K599 via electroporation (Duan et al. 2023). AVX seedlings were infected using the hairy root induction protocol, and transgenic roots were selected on hygromycin‐containing medium. Transgenic AVX seedlings with confirmed root transformation were transplanted into vermiculite and acclimatised for 1 month. Salt stress was applied by irrigating with 200 mM NaCl for 7 days, while controls received water. Post‐treatment measurements included plant height, root length, and root fresh weight. Reactive oxygen species (O_2_^−^) and malondialdehyde (MDA) levels were quantified using the OxiSelect ROS and TBARS Assay Kits (Cell Biolabs). Antioxidant enzyme activities (GSH‐Px, GR, CAT), proline (Pro) content and total flavonols content were measured with the BioVision Enzyme Activity Kits.

## Author Contributions

J.Z. and P.X. conceived and designed the study. B.A., S.W., J.Z., L.W., and F.W. prepared the materials and conducted the experiments. P.X. analysed the data. P.X. prepared the results. B.A. and S.W. identified the gene function. P.X. wrote the manuscript. J.Z., F.W., and Q.Y. edited and improved the manuscript. All authors approved the final manuscript.

## Conflicts of Interest

The authors declare no conflicts of interest.

## Supporting information


**Figure S1:** pbi70288‐sup‐0001‐Figures.docx.

**Table S1:** pbi70288‐sup‐0002‐Tables.xlsx.

## Data Availability

Newly generated sequencing datasets consisting of raw resequencing reads from 26 individuals, 18 APZ mRNA sequencing datasets, and three fully assembled telomere‐to‐telomere (T2T) genomes have been deposited in the National Genomics Data Center (NGDC) at the Beijing Institute of Genomics, Chinese Academy of Sciences. These comprehensive genomic resources are publicly available under BioProject accession number PRJCA036498.

## References

[pbi70288-bib-0001] Abubakar, A. S. , X. Feng , G. Gao , et al. 2022. “Genome Wide Characterization of R2R3 MYB Transcription Factor From *Apocynum venetum* Revealed Potential Stress Tolerance and Flavonoid Biosynthesis Genes.” Genomics 114: 110275. 35108591 10.1016/j.ygeno.2022.110275

[pbi70288-bib-0002] Alexander, D. H. , J. Novembre , and K. Lange . 2009. “Fast Model‐Based Estimation of Ancestry in Unrelated Individuals.” Genome Research 19: 1655–1664. 19648217 10.1101/gr.094052.109 PMC2752134

[pbi70288-bib-0003] Alioto, T. , E. Blanco , G. Parra , and R. Guigó . 2018. “Using Geneid to Identify Genes.” Current Protocols in Bioinformatics 64: e56. 30332532 10.1002/cpbi.56

[pbi70288-bib-0004] Armstrong, J. , G. Hickey , M. Diekhans , et al. 2020. “Progressive Cactus Is a Multiple‐Genome Aligner for the Thousand‐Genome Era.” Nature 587: 246–251. 33177663 10.1038/s41586-020-2871-y PMC7673649

[pbi70288-bib-0005] Bao, Y. , G. Hu , C. E. Grover , J. Conover , D. Yuan , and J. F. Wendel . 2019. “Unraveling Cis and Trans Regulatory Evolution During Cotton Domestication.” Nature Communications 10: 5399. 10.1038/s41467-019-13386-w PMC6881400 31776348

[pbi70288-bib-0006] Barrett, J. C. 2009. “Haploview: Visualization and Analysis of SNP Genotype Data.” Cold Spring Harbor Protocols 2009: pdb.ip71. 20147036 10.1101/pdb.ip71

[pbi70288-bib-0007] Baum, B. R. 1989. “PHYLIP: Phylogeny Inference Package. Version 3.2. Joel Felsenstein.” Quarterly Review of Biology 64: 539–541.

[pbi70288-bib-0008] Bomblies, K. , and D. Weigel . 2007. “Hybrid Necrosis: Autoimmunity as a Potential Gene‐Flow Barrier in Plant Species.” Nature Reviews Genetics 8: 382–393. 10.1038/nrg2082 17404584

[pbi70288-bib-0009] Burge, C. , and S. Karlin . 1997. “Prediction of Complete Gene Structures in Human Genomic DNA.” Journal of Molecular Biology 268: 78–94. 9149143 10.1006/jmbi.1997.0951

[pbi70288-bib-0010] Chen, M. , X.‐y. Zhao , and X.‐a. Zuo . 2015. “Comparative Reproductive Biology of *Apocynum venetum* L. in Wild and Managed Populations in the Arid Region of NW China.” Plant Systematics and Evolution 301: 1735–1745.

[pbi70288-bib-0011] Chen, S. , Y. Zhou , Y. Chen , and J. Gu . 2018. “Fastp: An Ultra‐Fast All‐In‐One FASTQ Preprocessor.” Bioinformatics 34: i884–i890. 30423086 10.1093/bioinformatics/bty560 PMC6129281

[pbi70288-bib-0012] Chen, X. , O. Schulz‐Trieglaff , R. Shaw , et al. 2016. “Manta: Rapid Detection of Structural Variants and Indels for Germline and Cancer Sequencing Applications.” Bioinformatics 32: 1220–1222. 26647377 10.1093/bioinformatics/btv710

[pbi70288-bib-0013] Cheng, H. , G. T. Concepcion , X. Feng , H. Zhang , and H. Li . 2021. “Haplotype‐Resolved de Novo Assembly Using Phased Assembly Graphs With Hifiasm.” Nature Methods 18: 170–175. 33526886 10.1038/s41592-020-01056-5 PMC7961889

[pbi70288-bib-0014] Cheng, Y. , J. Sun , M. Jiang , et al. 2023. “Chromosome‐Scale Genome Sequence of Suaeda Glauca Sheds Light on Salt Stress Tolerance in Halophytes.” Horticulture Research 10: uhad161. 37727702 10.1093/hr/uhad161 PMC10506132

[pbi70288-bib-0015] Christie, J. R. , and M. Beekman . 2017. “Uniparental Inheritance Promotes Adaptive Evolution in Cytoplasmic Genomes.” Molecular Biology and Evolution 34: 677–691. 28025277 10.1093/molbev/msw266 PMC5896580

[pbi70288-bib-0016] Cingolani, P. , A. Platts , L. L. Wang , et al. 2012. “A Program for Annotating and Predicting the Effects of Single Nucleotide Polymorphisms, SnpEff.” Fly 6: 80–92. 22728672 10.4161/fly.19695 PMC3679285

[pbi70288-bib-0017] Danecek, P. , A. Auton , G. Abecasis , et al. 2011. “The Variant Call Format and VCFtools.” Bioinformatics 27: 2156–2158. 21653522 10.1093/bioinformatics/btr330 PMC3137218

[pbi70288-bib-0018] De Bie, T. , N. Cristianini , J. P. Demuth , and M. W. Hahn . 2006. “CAFE: A Computational Tool for the Study of Gene Family Evolution.” Bioinformatics 22: 1269–1271. 16543274 10.1093/bioinformatics/btl097

[pbi70288-bib-0019] Dorjee, T. , J. Tan , Q. Zuo , et al. 2024. “Chromosome‐Scale Genome Analysis of *Apocynum venetum* Sheds Light on *Apocynum* Phylogenetics, Bast Fiber Development, and Flavonoid Synthesis.” Industrial Crops and Products 212: 118325.

[pbi70288-bib-0020] Duan, Z. , S. Wang , Z. Zhang , et al. 2023. “The MabHLH11 Transcription Factor Interacting With MaMYB4 Acts Additively in Increasing Plant Scopolin Biosynthesis.” Crop Journal 11: 1675–1685.

[pbi70288-bib-0021] Dudchenko, O. , S. S. Batra , A. D. Omer , et al. 2017. “De Novo Assembly of the *Aedes aegypti* Genome Using Hi‐C Yields Chromosome‐Length Scaffolds.” Science 356: 92–95. 28336562 10.1126/science.aal3327 PMC5635820

[pbi70288-bib-0022] Dvorak, J. , L. Wang , T. Zhu , et al. 2018. “Structural Variation and Rates of Genome Evolution in the Grass Family Seen Through Comparison of Sequences of Genomes Greatly Differing in Size.” Plant Journal 95: 487–503. 10.1111/tpj.13964 29770515

[pbi70288-bib-0023] Dvorkina, T. , A. V. Bzikadze , and P. A. Pevzner . 2020. “The String Decomposition Problem and Its Applications to Centromere Analysis and Assembly.” Bioinformatics 36: i93–i101. 32657390 10.1093/bioinformatics/btaa454 PMC7428072

[pbi70288-bib-0024] Edgar, R. C. 2004. “MUSCLE: Multiple Sequence Alignment With High Accuracy and High Throughput.” Nucleic Acids Research 32: 1792–1797. 15034147 10.1093/nar/gkh340 PMC390337

[pbi70288-bib-0025] Emms, D. M. , and S. Kelly . 2019. “OrthoFinder: Phylogenetic Orthology Inference for Comparative Genomics.” Genome Biology 20: 238. 31727128 10.1186/s13059-019-1832-y PMC6857279

[pbi70288-bib-0026] Fu, H.‐M. , C.‐L. Yin , Z.‐Y. Shen , and M.‐H. Yang . 2022. “Flavonoids From the Leaves of *Apocynum venetum* and Their Anti‐Inflammatory Activity.” Journal of Chemical Research 46: 17475198211073871.

[pbi70288-bib-0027] Gao, G. , P. Chen , J. Chen , et al. 2019. Genomic Survey, Transcriptome, and Metabolome Analysis of Apocynum venetum and Apocynum hendersonii to Reveal Major Flavonoid Biosynthesis Pathways. Vol. 9, 296. Metabolites. 10.3390/metabo9120296 PMC6950674 31817331

[pbi70288-bib-0028] Gao, S. , X. Yang , H. Guo , X. Zhao , B. Wang , and K. Ye . 2023. “HiCAT: A Tool for Automatic Annotation of Centromere Structure.” Genome Biology 24: 58. 36978122 10.1186/s13059-023-02900-5 PMC10053651

[pbi70288-bib-0029] Goel, M. , H. Sun , W. B. Jiao , and K. Schneeberger . 2019. “SyRI: Finding Genomic Rearrangements and Local Sequence Differences From Whole‐Genome Assemblies.” Genome Biology 20: 277. 31842948 10.1186/s13059-019-1911-0 PMC6913012

[pbi70288-bib-0030] Goulet, B. E. , F. Roda , and R. Hopkins . 2017. “Hybridization in Plants: Old Ideas, New Techniques.” Plant Physiology 173: 65–78. 27895205 10.1104/pp.16.01340 PMC5210733

[pbi70288-bib-0031] Grant, P. R. , and B. R. Grant . 1992. “Hybridization of Bird Species.” Science 256: 193–197. 17744718 10.1126/science.256.5054.193

[pbi70288-bib-0032] Gu, Z. , and B. Han . 2024. “Unlocking the Mystery of Heterosis Opens the Era of Intelligent Rice Breeding.” Plant Physiology 196: 735–744. 39115386 10.1093/plphys/kiae385 PMC11444277

[pbi70288-bib-0033] Haas, B. J. , S. L. Salzberg , W. Zhu , et al. 2008. “Automated Eukaryotic Gene Structure Annotation Using EVidenceModeler and the Program to Assemble Spliced Alignments.” Genome Biology 9: R7. 18190707 10.1186/gb-2008-9-1-r7 PMC2395244

[pbi70288-bib-0034] Harmer, S. L. , J. B. Hogenesch , M. Straume , et al. 2000. “Orchestrated Transcription of Key Pathways in Arabidopsis by the Circadian Clock.” Science 290: 2110–2113. 11118138 10.1126/science.290.5499.2110

[pbi70288-bib-0035] Hochholdinger, F. , and J. A. Baldauf . 2018. “Heterosis in Plants.” Current Biology 28: R1089–R1092. 30253145 10.1016/j.cub.2018.06.041

[pbi70288-bib-0036] Hu, J. , Z. Wang , F. Liang , S.‐L. Liu , K. Ye , and D.‐P. Wang . 2024. “NextPolish2: A Repeat‐Aware Polishing Tool for Genomes Assembled Using HiFi Long Reads.” Genomics, Proteomics & Bioinformatics 22: qzad009. 10.1093/gpbjnl/qzad009 PMC12016036 38862426

[pbi70288-bib-0037] Hu, J. , Z. Wang , Z. Sun , et al. 2024. “NextDenovo: An Efficient Error Correction and Accurate Assembly Tool for Noisy Long Reads.” Genome Biology 25: 107. 38671502 10.1186/s13059-024-03252-4 PMC11046930

[pbi70288-bib-0038] Huang, X. , S. Yang , J. Gong , et al. 2016. “Genomic Architecture of Heterosis for Yield Traits in Rice.” Nature 537: 629–633. 27602511 10.1038/nature19760

[pbi70288-bib-0039] Jacob, A. , G. Evanno , B. A. Von Siebenthal , C. Grossen , and C. Wedekind . 2010. “Effects of Different Mating Scenarios on Embryo Viability in Brown Trout.” Molecular Ecology 19: 5296–5307. 21040055 10.1111/j.1365-294X.2010.04884.x

[pbi70288-bib-0040] Jiang, L. , X. Wu , Z. Zhao , et al. 2021. “Luobuma (Apocynum)—Cash Crops for Saline Lands.” Industrial Crops and Products 173: 114146.

[pbi70288-bib-0041] Jin, J.‐J. , W.‐B. Yu , J.‐B. Yang , et al. 2020. “GetOrganelle: A Fast and Versatile Toolkit for Accurate De Novo Assembly of Organelle Genomes.” Genome Biology 21: 241. 32912315 10.1186/s13059-020-02154-5 PMC7488116

[pbi70288-bib-0042] Johnson, S. A. , L. P. Bruederle , and D. F. Tomback . 1998. “A Mating System Conundrum: Hybridization in *Apocynum* (*Apocynaceae*).” American Journal of Botany 85: 1316–1323. 21685017

[pbi70288-bib-0043] Kakoulidou, I. , and F. Johannes . 2024. “DNA Methylation Remodeling in F1 Hybrids.” Plant Journal 118: 671–681. 10.1111/tpj.16137 36752648

[pbi70288-bib-0044] Kim, D. , G. Pertea , C. Trapnell , H. Pimentel , R. Kelley , and S. L. Salzberg . 2013. “TopHat2: Accurate Alignment of Transcriptomes in the Presence of Insertions, Deletions and Gene Fusions.” Genome Biology 14: R36. 23618408 10.1186/gb-2013-14-4-r36 PMC4053844

[pbi70288-bib-0045] Kokot, M. , M. Dlugosz , and S. Deorowicz . 2017. “KMC 3: Counting and Manipulating k‐Mer Statistics.” Bioinformatics 33: 2759–2761. 28472236 10.1093/bioinformatics/btx304

[pbi70288-bib-0046] Korf, I. 2004. “Gene Finding in Novel Genomes.” BMC Bioinformatics 5: 59. 15144565 10.1186/1471-2105-5-59 PMC421630

[pbi70288-bib-0047] Langmead, B. , and S. L. Salzberg . 2012. “Fast Gapped‐Read Alignment With Bowtie 2.” Nature Methods 9: 357–359. 22388286 10.1038/nmeth.1923 PMC3322381

[pbi70288-bib-0048] Letunic, I. , and P. Bork . 2019. “Interactive Tree of Life (iTOL) v4: Recent Updates and New Developments.” Nucleic Acids Research 47: W256–W259. 30931475 10.1093/nar/gkz239 PMC6602468

[pbi70288-bib-0049] Li, C. , G. Huang , F. Tan , X. Zhou , J. Mu , and X. Zhao . 2019. “In Vitro Analysis of Antioxidant, Anticancer, and Bioactive Components of *Apocynum venetum* Tea Extracts.” Journal of Food Quality 13: 2465341.

[pbi70288-bib-0050] Li, H. 2018. “Minimap2: Pairwise Alignment for Nucleotide Sequences.” Bioinformatics 34: 3094–3100. 29750242 10.1093/bioinformatics/bty191 PMC6137996

[pbi70288-bib-0051] Li, H. , and R. Durbin . 2009. “Fast and Accurate Short Read Alignment With Burrows‐Wheeler Transform.” Bioinformatics 25: 1754–1760. 19451168 10.1093/bioinformatics/btp324 PMC2705234

[pbi70288-bib-0052] Li, H. , B. Handsaker , A. Wysoker , et al. 2009. “The Sequence Alignment/Map Format and SAMtools.” Bioinformatics 25: 2078–2079. 19505943 10.1093/bioinformatics/btp352 PMC2723002

[pbi70288-bib-0053] Li, Y. , S. Zhang , W. Jiang , and D. Liu . 2013. “Cadmium Accumulation, Activities of Antioxidant Enzymes, and Malondialdehyde (MDA) Content in *Pistia stratiotes* L.” Environmental Science and Pollution Research 20: 1117–1123. 22791349 10.1007/s11356-012-1054-2

[pbi70288-bib-0054] Lin, Y. , C. Ye , X. Li , et al. 2023. “quarTeT: A Telomere‐To‐Telomere Toolkit for Gap‐Free Genome Assembly and Centromeric Repeat Identification.” Horticulture Research 10: uhad127. 37560017 10.1093/hr/uhad127 PMC10407605

[pbi70288-bib-0055] Liu, C. , B. Mao , Y. Zhang , et al. 2024. “The OsWRKY72–OsAAT30/OsGSTU26 Module Mediates Reactive Oxygen Species Scavenging to Drive Heterosis for Salt Tolerance in Hybrid Rice.” Journal of Integrative Plant Biology 66: 709–730. 38483018 10.1111/jipb.13640

[pbi70288-bib-0056] Liu, J. , L.‐Y. Chen , P. Zhou , et al. 2021. “Sex Biased Expression of Hormone Related Genes at Early Stage of Sex Differentiation in Papaya Flowers.” Horticulture Research 8: 147. 34193826 10.1038/s41438-021-00581-4 PMC8245580

[pbi70288-bib-0057] Liu, S. , L. Zhang , Y. Sang , et al. 2022. “Demographic History and Natural Selection Shape Patterns of Deleterious Mutation Load and Barriers to Introgression Across Populus Genome.” Molecular Biology and Evolution 39: msac008. 35022759 10.1093/molbev/msac008 PMC8826634

[pbi70288-bib-0058] Liu, W. , Y. Feng , S. Yu , et al. 2021. “The Flavonoid Biosynthesis Network in Plants.” International Journal of Molecular Sciences 22, no. 23: 12824. 34884627 10.3390/ijms222312824 PMC8657439

[pbi70288-bib-0059] Liu, W. , Y. Zhang , H. He , G. He , and X. W. Deng . 2022. “From Hybrid Genomes to Heterotic Trait Output: Challenges and Opportunities.” Current Opinion in Plant Biology 66: 102193. 35219140 10.1016/j.pbi.2022.102193

[pbi70288-bib-0060] Liu, Y. , H. Wu , H. Chen , et al. 2015. “A Gene Cluster Encoding Lectin Receptor Kinases Confers Broad‐Spectrum and Durable Insect Resistance in Rice.” Nature Biotechnology 33: 301–305. 10.1038/nbt.3069 25485617

[pbi70288-bib-0061] Lohse, M. , O. Drechsel , and R. Bock . 2007. “OrganellarGenomeDRAW (OGDRAW): A Tool for the Easy Generation of High‐Quality Custom Graphical Maps of Plastid and Mitochondrial Genomes.” Current Genetics 52: 267–274. 17957369 10.1007/s00294-007-0161-y

[pbi70288-bib-0062] Majoros, W. H. , M. Pertea , and S. L. Salzberg . 2004. “TigrScan and GlimmerHMM: Two Open Source Ab Initio Eukaryotic Gene‐Finders.” Bioinformatics 20: 2878–2879. 15145805 10.1093/bioinformatics/bth315

[pbi70288-bib-0063] Marçais, G. , A. L. Delcher , A. M. Phillippy , R. Coston , S. L. Salzberg , and A. Zimin . 2018. “MUMmer4: A Fast and Versatile Genome Alignment System.” PLoS Computational Biology 14: e1005944. 29373581 10.1371/journal.pcbi.1005944 PMC5802927

[pbi70288-bib-0064] Marçais, G. , and C. Kingsford . 2011. “A Fast, Lock‐Free Approach for Efficient Parallel Counting of Occurrences of k‐Mers.” Bioinformatics 27: 764–770. 21217122 10.1093/bioinformatics/btr011 PMC3051319

[pbi70288-bib-0065] McKenna, A. , M. Hanna , E. Banks , et al. 2010. “The Genome Analysis Toolkit: A MapReduce Framework for Analyzing Next‐Generation DNA Sequencing Data.” Genome Research 20: 1297–1303. 20644199 10.1101/gr.107524.110 PMC2928508

[pbi70288-bib-0066] Minh, B. Q. , H. A. Schmidt , O. Chernomor , et al. 2020. “IQ‐TREE 2: New Models and Efficient Methods for Phylogenetic Inference in the Genomic Era.” Molecular Biology and Evolution 37: 1530–1534. 32011700 10.1093/molbev/msaa015 PMC7182206

[pbi70288-bib-0067] Nurk, S. , B. P. Walenz , A. Rhie , et al. 2020. “HiCanu: Accurate Assembly of Segmental Duplications, Satellites, and Allelic Variants From High‐Fidelity Long Reads.” Genome Research 30: 1291–1305. 32801147 10.1101/gr.263566.120 PMC7545148

[pbi70288-bib-0068] Ou, S. , and N. Jiang . 2018. “LTR_retriever: A Highly Accurate and Sensitive Program for Identification of Long Terminal Repeat Retrotransposons.” Plant Physiology 176: 1410–1422. 29233850 10.1104/pp.17.01310 PMC5813529

[pbi70288-bib-0069] Ou, S. , W. Su , Y. Liao , et al. 2019. “Benchmarking Transposable Element Annotation Methods for Creation of a Streamlined, Comprehensive Pipeline.” Genome Biology 20: 275. 31843001 10.1186/s13059-019-1905-y PMC6913007

[pbi70288-bib-0070] Pankova, E. I. , I. P. Aidarov , D. L. Golovanov , and I. A. Yamnova . 2015. “Salinization as the Main Soil‐Forming Process in Soils of Natural Oases in the Gobi Desert.” Eurasian Soil Science 48: 1017–1028.

[pbi70288-bib-0071] Patro, R. , G. Duggal , M. I. Love , R. A. Irizarry , and C. Kingsford . 2017. “Salmon Provides Fast and Bias‐Aware Quantification of Transcript Expression.” Nature Methods 14: 417–419. 28263959 10.1038/nmeth.4197 PMC5600148

[pbi70288-bib-0072] Patterson, N. , A. L. Price , and D. Reich . 2006. “Population Structure and Eigenanalysis. *PLOS* .” Genetics 2: e190. 10.1371/journal.pgen.0020190 PMC1713260 17194218

[pbi70288-bib-0073] Price, M. N. , P. S. Dehal , and A. P. Arkin . 2009. “FastTree: Computing Large Minimum Evolution Trees With Profiles Instead of a Distance Matrix.” Molecular Biology and Evolution 26: 1641–1650. 19377059 10.1093/molbev/msp077 PMC2693737

[pbi70288-bib-0074] Quinlan, A. R. , and I. M. Hall . 2010. “BEDTools: A Flexible Suite of Utilities for Comparing Genomic Features.” Bioinformatics 26: 841–842. 20110278 10.1093/bioinformatics/btq033 PMC2832824

[pbi70288-bib-0075] Racimo, F. 2016. “Testing for Ancient Selection Using Cross‐Population Allele Frequency Differentiation.” Genetics 202: 733–750. 26596347 10.1534/genetics.115.178095 PMC4788246

[pbi70288-bib-0076] Rhie, A. , B. P. Walenz , S. Koren , and A. M. Phillippy . 2020. “Merqury: Reference‐Free Quality, Completeness, and Phasing Assessment for Genome Assemblies.” Genome Biology 21: 245. 32928274 10.1186/s13059-020-02134-9 PMC7488777

[pbi70288-bib-0077] Rieseberg, L. H. , and S. E. Carney . 1998. “Plant Hybridization.” New Phytologist 140: 599–624. 33862960 10.1046/j.1469-8137.1998.00315.x

[pbi70288-bib-0078] Robinson, M. D. , D. J. McCarthy , and G. K. Smyth . 2010. “edgeR: A Bioconductor Package for Differential Expression Analysis of Digital Gene Expression Data.” Bioinformatics 26: 139–140. 19910308 10.1093/bioinformatics/btp616 PMC2796818

[pbi70288-bib-0079] Sander, J. D. , and J. K. Joung . 2014. “CRISPR‐Cas Systems for Editing, Regulating and Targeting Genomes.” Nature Biotechnology 32: 347–355. 10.1038/nbt.2842 PMC4022601 24584096

[pbi70288-bib-0080] Sedlazeck, F. J. , P. Rescheneder , M. Smolka , et al. 2018. “Accurate Detection of Complex Structural Variations Using Single‐Molecule Sequencing.” Nature Methods 15: 461–468. 29713083 10.1038/s41592-018-0001-7 PMC5990442

[pbi70288-bib-0081] Servant, N. , N. Varoquaux , B. R. Lajoie , et al. 2015. “HiC‐Pro: An Optimized and Flexible Pipeline for Hi‐C Data Processing.” Genome Biology 16: 259. 26619908 10.1186/s13059-015-0831-x PMC4665391

[pbi70288-bib-0082] Shao, D. , G. Gao , A. S. Abubakar , et al. 2022. “Total Flavonoids Extracts of Apocynum L. From the Ili River Valley Region at Different Harvesting Periods and Bioactivity Analysis.” Molecules 27: 7343. 10.3390/molecules27217343. 36364168 PMC9655940

[pbi70288-bib-0083] Shao, L. , F. Xing , C. Xu , et al. 2019. “Patterns of Genome‐Wide Allele‐Specific Expression in Hybrid Rice and the Implications on the Genetic Basis of Heterosis.” Proceedings of the National Academy of Sciences 116: 5653–5658. 10.1073/pnas.1820513116 PMC6431163 30833384

[pbi70288-bib-0084] Shi, J. , and C. Liang . 2019. “Generic Repeat Finder: A High‐Sensitivity Tool for Genome‐Wide De Novo Repeat Detection.” Plant Physiology 180: 1803–1815. 31152127 10.1104/pp.19.00386 PMC6670090

[pbi70288-bib-0085] Simão, F. A. , R. M. Waterhouse , P. Ioannidis , E. V. Kriventseva , and E. M. Zdobnov . 2015. “BUSCO: Assessing Genome Assembly and Annotation Completeness With Single‐Copy Orthologs.” Bioinformatics 31: 3210–3212. 26059717 10.1093/bioinformatics/btv351

[pbi70288-bib-0086] Smolka, M. , L. F. Paulin , C. M. Grochowski , et al. 2024. “Detection of Mosaic and Population‐Level Structural Variants With Sniffles2.” Nature Biotechnology 42: 1571–1580. 10.1038/s41587-023-02024-y PMC11217151 38168980

[pbi70288-bib-0087] Soltis, P. S. , and D. E. Soltis . 2009. “The Role of Hybridization in Plant Speciation.” Annual Review of Plant Biology 60: 561–588. 10.1146/annurev.arplant.043008.092039 19575590

[pbi70288-bib-0088] Srikanth, A. , and M. Schmid . 2011. “Regulation of Flowering Time: All Roads Lead to Rome.” Cellular and Molecular Life Sciences 68: 2013–2037. 21611891 10.1007/s00018-011-0673-y PMC11115107

[pbi70288-bib-0089] Stamatakis, A. 2014. “RAxML Version 8: A Tool for Phylogenetic Analysis and Post‐Analysis of Large Phylogenies.” Bioinformatics 30: 1312–1313. 24451623 10.1093/bioinformatics/btu033 PMC3998144

[pbi70288-bib-0090] Stanke, M. , M. Diekhans , R. Baertsch , and D. Haussler . 2008. “Using Native and Syntenically Mapped cDNA Alignments to Improve De Novo Gene Finding.” Bioinformatics 24: 637–644. 18218656 10.1093/bioinformatics/btn013

[pbi70288-bib-0091] Su, W. , X. Gu , and T. Peterson . 2019. “TIR‐Learner, a New Ensemble Method for TIR Transposable Element Annotation, Provides Evidence for Abundant New Transposable Elements in the Maize Genome.” Molecular Plant 12: 447–460. 30802553 10.1016/j.molp.2019.02.008

[pbi70288-bib-0092] Tang, S. , A. Lomsadze , and M. Borodovsky . 2015. “Identification of Protein Coding Regions in RNA Transcripts.” Nucleic Acids Research 43: e78. 25870408 10.1093/nar/gkv227 PMC4499116

[pbi70288-bib-0093] Terhorst, J. , J. A. Kamm , and Y. S. Song . 2017. “Robust and Scalable Inference of Population History From Hundreds of Unphased Whole Genomes.” Nature Genetics 49: 303–309. 28024154 10.1038/ng.3748 PMC5470542

[pbi70288-bib-0094] Thevs, N. , S. Zerbe , Y. Kyosev , et al. 2012. “ *Apocynum venetum* L. and *apocynum pictum* Schrenk (Apocynaceae) as Multi‐Functional and Multi‐Service Plant Species in Central Asia: A Review on Biology, Ecology, and Utilization.” Journal of Applied Botany and Food Quality 85: 159–167.

[pbi70288-bib-0095] Tillich, M. , P. Lehwark , T. Pellizzer , et al. 2017. “GeSeq—Versatile and Accurate Annotation of Organelle Genomes.” Nucleic Acids Research 45: W6–W11. 28486635 10.1093/nar/gkx391 PMC5570176

[pbi70288-bib-0096] Tohge, T. , L. P. de Souza , and A. R. Fernie . 2017. “Current Understanding of the Pathways of Flavonoid Biosynthesis in Model and Crop Plants.” Journal of Experimental Botany 68: 4013–4028. 28922752 10.1093/jxb/erx177

[pbi70288-bib-0097] Turnbull, J. J. , J.‐i. Nakajima , R. W. D. Welford , M. Yamazaki , K. Saito , and C. J. Schofield . 2004. “Mechanistic Studies on Three 2‐Oxoglutarate‐Dependent Oxygenases of Flavonoid Biosynthesis: Anthocyanidin Synthase, Flavonol Synthase, and Flavanone 3beta‐Hydroxylase.” Journal of Biological Chemistry 279: 1206–1216. 14570878 10.1074/jbc.M309228200

[pbi70288-bib-0098] van Belleghem, S. M. , P. Rastas , A. Papanicolaou , et al. 2017. “Complex Modular Architecture Around a Simple Toolkit of Wing Pattern Genes.” Nature Ecology & Evolution 1: 52. 28523290 10.1038/s41559-016-0052 PMC5432014

[pbi70288-bib-0099] Vollger, M. R. , P. Kerpedjiev , A. M. Phillippy , and E. E. Eichler . 2022. “StainedGlass: Interactive Visualization of Massive Tandem Repeat Structures With Identity Heatmaps.” Bioinformatics 38: 2049–2051. 35020798 10.1093/bioinformatics/btac018 PMC8963321

[pbi70288-bib-0100] Wang, P. , M. Gu , X. Yu , et al. 2022. “Allele‐Specific Expression and Chromatin Accessibility Contribute to Heterosis in Tea Plants ( *Camellia sinensis* ).” Plant Journal 112: 1194–1211. 10.1111/tpj.16004 36219505

[pbi70288-bib-0101] Wang, Z. , Y. Jiang , H. Bi , et al. 2021. “Hybrid Speciation via Inheritance of Alternate Alleles of Parental Isolating Genes.” Molecular Plant 14: 208–222. 33220509 10.1016/j.molp.2020.11.008

[pbi70288-bib-0102] Xiao, H. , Y. Wang , W. Liu , et al. 2025. “Impacts of Reproductive Systems on Grapevine Genome and Breeding.” Nature Communications 16: 2031. 10.1038/s41467-025-56817-7 PMC11876636 40032836

[pbi70288-bib-0103] Xiong, W. , L. He , J. Lai , H. K. Dooner , and C. Du . 2014. “HelitronScanner Uncovers a Large Overlooked Cache of Helitron Transposons in Many Plant Genomes.” Proceedings of the National Academy of Sciences of the United States of America 111: 10263–10268. 24982153 10.1073/pnas.1410068111 PMC4104883

[pbi70288-bib-0104] Xu, X.‐D. , R.‐P. Zhao , L. Xiao , et al. 2023. “Telomere‐To‐Telomere Assembly of Cassava Genome Reveals the Evolution of Cassava and Divergence of Allelic Expression.” Horticulture Research 10: uhad200. 38023477 10.1093/hr/uhad200 PMC10673656

[pbi70288-bib-0105] Xu, Z. , and H. Wang . 2007. “LTR_FINDER: An Efficient Tool for the Prediction of Full‐Length LTR Retrotransposons.” Nucleic Acids Research 35: W265–W268. 17485477 10.1093/nar/gkm286 PMC1933203

[pbi70288-bib-0106] Xu, Z. , M. Wang , T. Ren , et al. 2021. “Comparative Transcriptome Analysis Reveals the Molecular Mechanism of Salt Tolerance in *Apocynum venetum* .” Plant Physiology and Biochemistry 167: 816–830. 34530326 10.1016/j.plaphy.2021.08.043

[pbi70288-bib-0107] Xue, Y. , X. Cao , X. Chen , et al. 2025. “Epigenetics in the Modern Era of Crop Improvements.” Science China Life Sciences 68: 1570–1609. 39808224 10.1007/s11427-024-2784-3

[pbi70288-bib-0108] Yang, Z. 2007. “PAML 4: Phylogenetic Analysis by Maximum Likelihood.” Molecular Biology and Evolution 24: 1586–1591. 17483113 10.1093/molbev/msm088

[pbi70288-bib-0109] Yang, Z. , X. Ge , Z. Yang , et al. 2019. “Extensive Intraspecific Gene Order and Gene Structural Variations in Upland Cotton Cultivars.” Nature Communications 10: 2989. 10.1038/s41467-019-10820-x PMC6611876 31278252

[pbi70288-bib-0110] Yu, G. , L. G. Wang , Y. Han , and Q. Y. He . 2012. “clusterProfiler: An R Package for Comparing Biological Themes Among Gene Clusters.” OMICS: A Journal of Integrative Biology 16: 284–287. 22455463 10.1089/omi.2011.0118 PMC3339379

[pbi70288-bib-0111] Yu, Z. , B. Cui , J. Xiao , et al. 2024. “Dosage Effect Genes Modulate Grain Development in Synthesized *Triticum durum* ‐ *Haynaldia villosa* Allohexaploid.” Journal of Genetics and Genomics 51: 1089–1100. 38670432 10.1016/j.jgg.2024.04.010

[pbi70288-bib-0112] Zhang, X. , R. Wu , Y. Wang , J. Yu , and H. Tang . 2020. “Unzipping Haplotypes in Diploid and Polyploid Genomes.” Computational and Structural Biotechnology Journal 33: 1994–2001. 10.1016/j.csbj.2019.11.011 PMC6938933 31908732

[pbi70288-bib-0113] Zhang, Y. , J. Chu , H. Cheng , and H. Li . 2023. “De Novo Reconstruction of Satellite Repeat Units From Sequence Data.” Genome Research 18: 66–72. 10.1101/gr.278005.123 PMC10760446 37918962

[pbi70288-bib-0114] Zhang, Z. , X. Jin , Z. Liu , J. Zhang , and W. Liu . 2021. “Genome‐Wide Identification of FAD Gene Family and Functional Analysis of MsFAD3.1 Involved in the Accumulation of α‐Linolenic Acid in Alfalfa.” Crop Science 61: 566–579.

