# Supplementary material for: Hybridization Drives Trait Integration in Telomere‐To‐Telomere Apocynum Genomes
Source: Plant Biotechnol J. 2025 Aug 6;23(11):5129–45. doi: 10.1111/pbi.70288 (PMC12576458; doi:10.1111/pbi.70288)
Supplement: Supplementary file 1 — Figure S1: pbi70288‐sup‐0001‐Figures.docx. [file PBI-23-5129-s002.docx]

# Hybridization drives trait integration in telomere-to-telomere *Apocynum* genomes

Pan Xu^1#^, Fan Wu^1,2#^, Qi Yan^1#^, Bao Ao^1^, Shengsheng Wang^1^, Lijun Chen^1^, Li Wang^1^, Jiyu Zhang^1^*

^1^ State Key Laboratory of Herbage Improvement and Grassland Agro-ecosystems, College of Pastoral Agriculture Science and Technology, Lanzhou University, Lanzhou, 730020 China

^2^ Key Laboratory of Grassland Resources of the Ministry of Education, College of Grassland Science, Inner Mongolia Agricultural University, Hohhot, 010011 China

**Supplementary Figures**

**A**


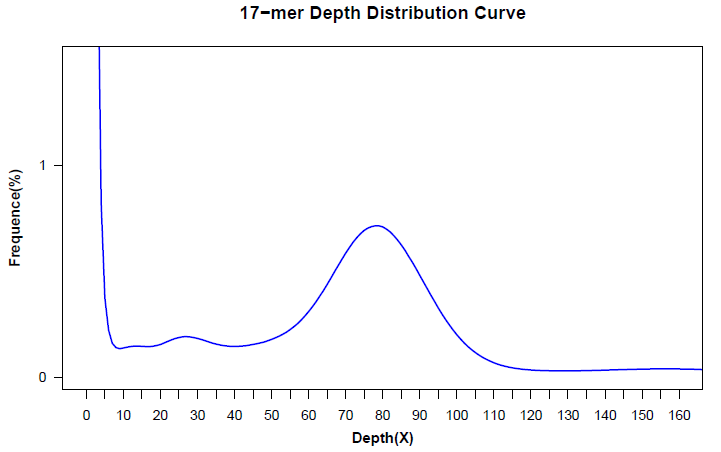


**B**


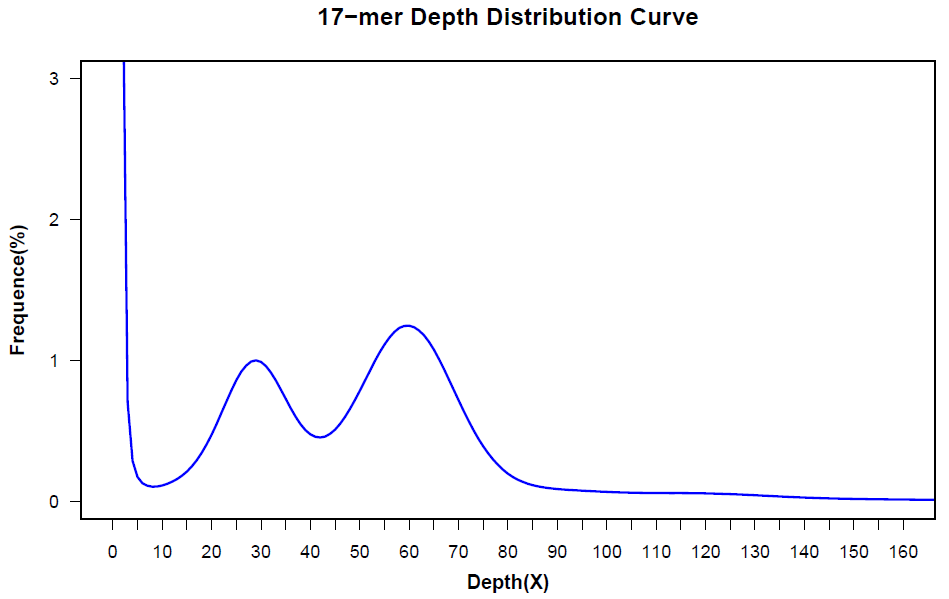


**C**


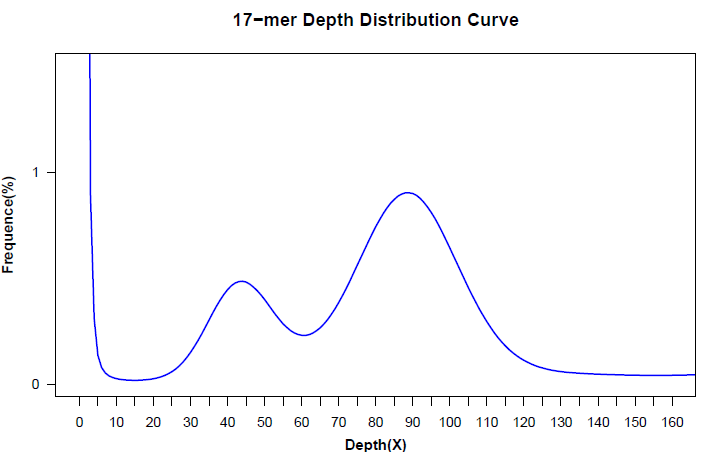


Fig. S1 K-mer frequency distribution of AVX (A), APZ (B), and AHG (C)

A B


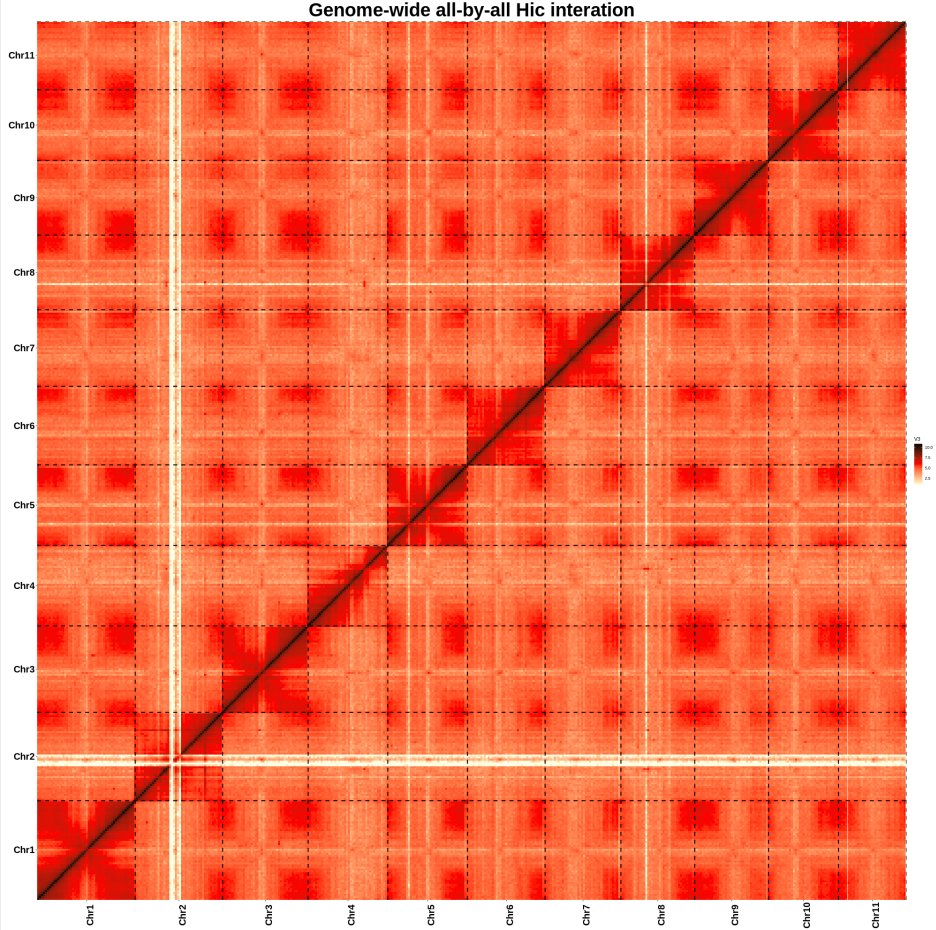

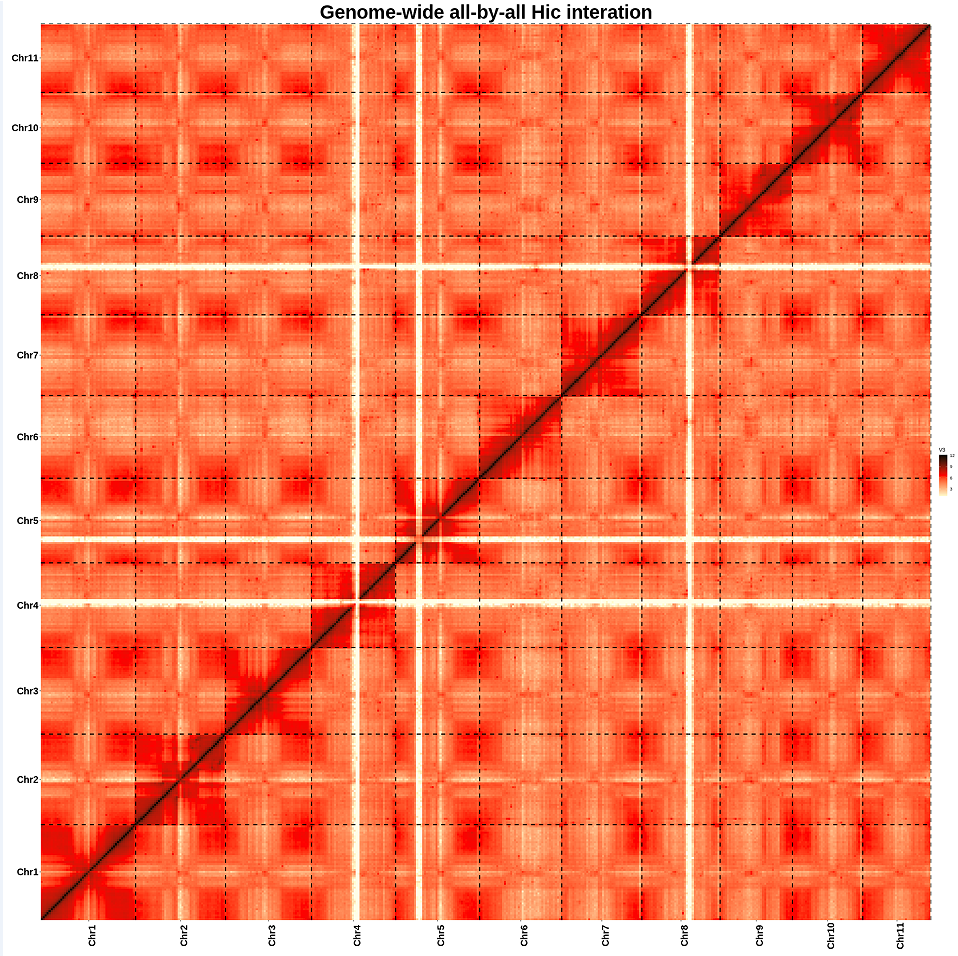


C


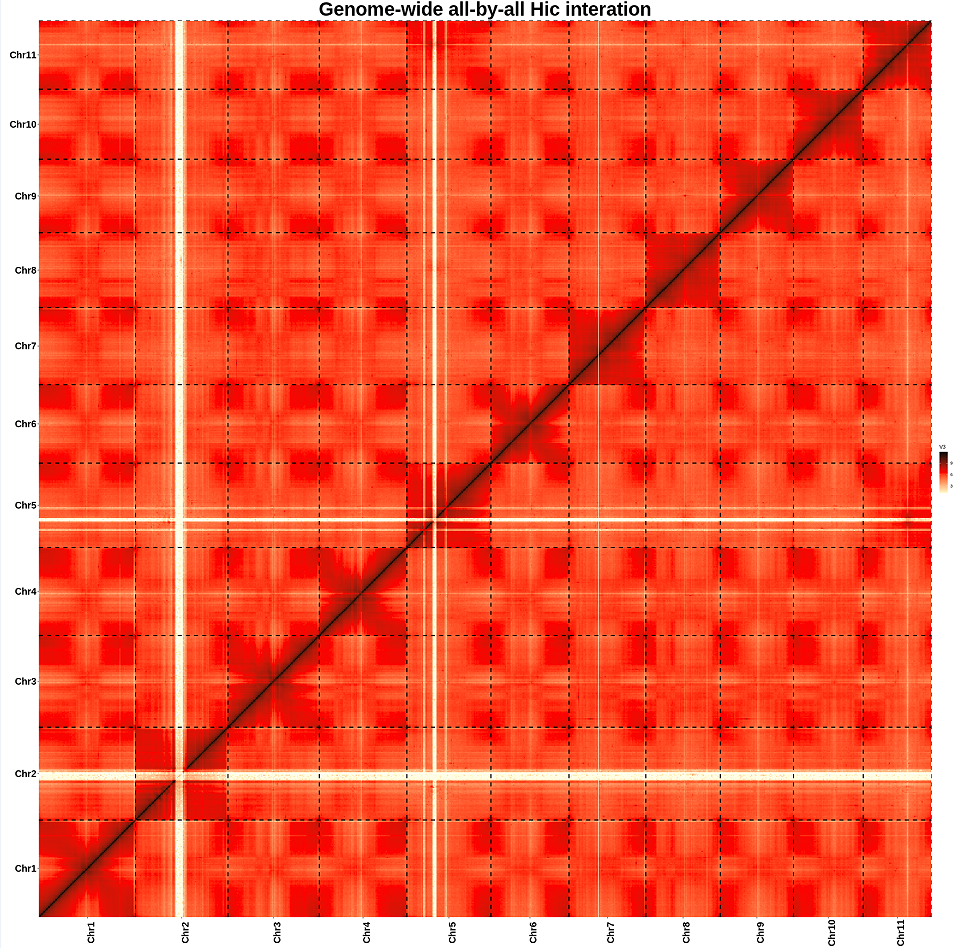


Fig. S2 Inter-chromosomal contact matrix of AVX (A), APZ (B) and AHG (C). The intensity of pixels represents the normalized count of Hi-C links between 100 kb windows on 11 chromosomes on a logarithmic scale.

A


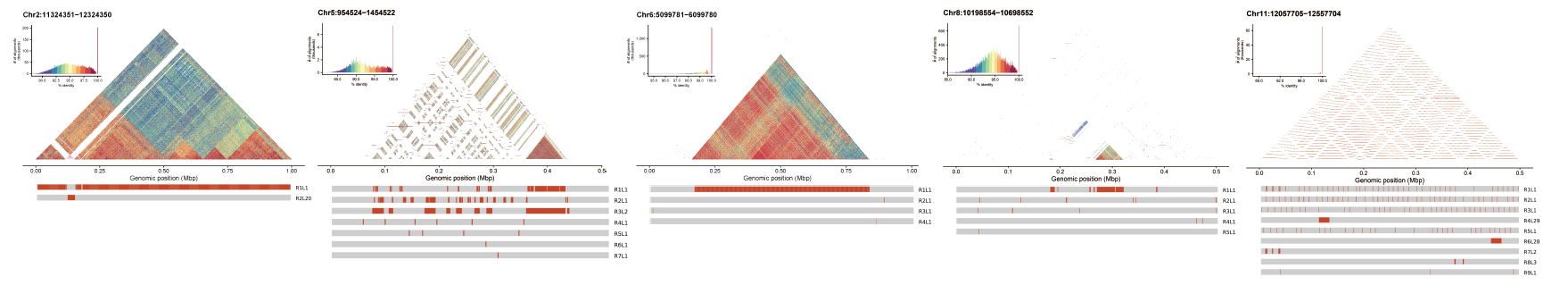


B


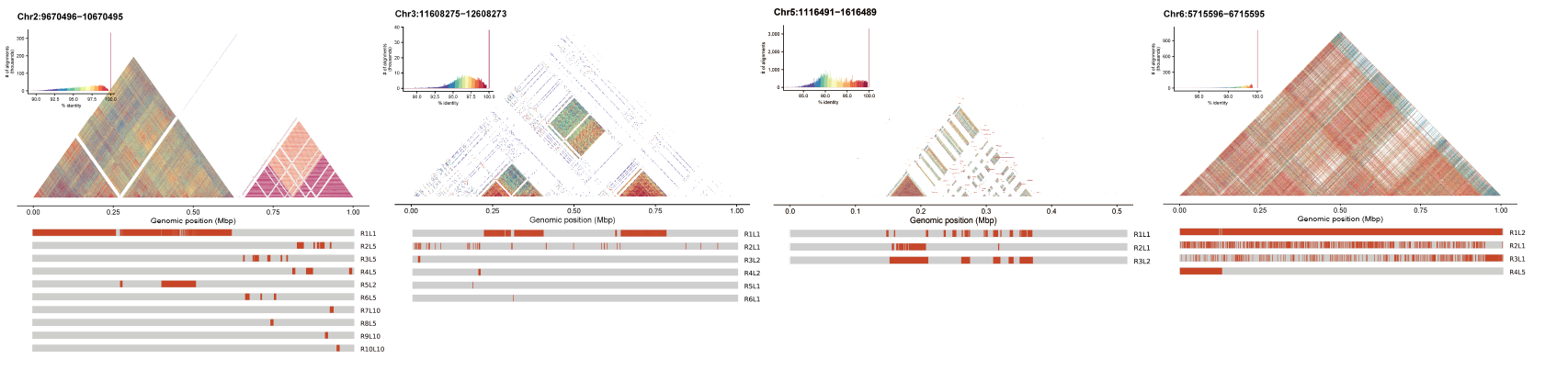


C


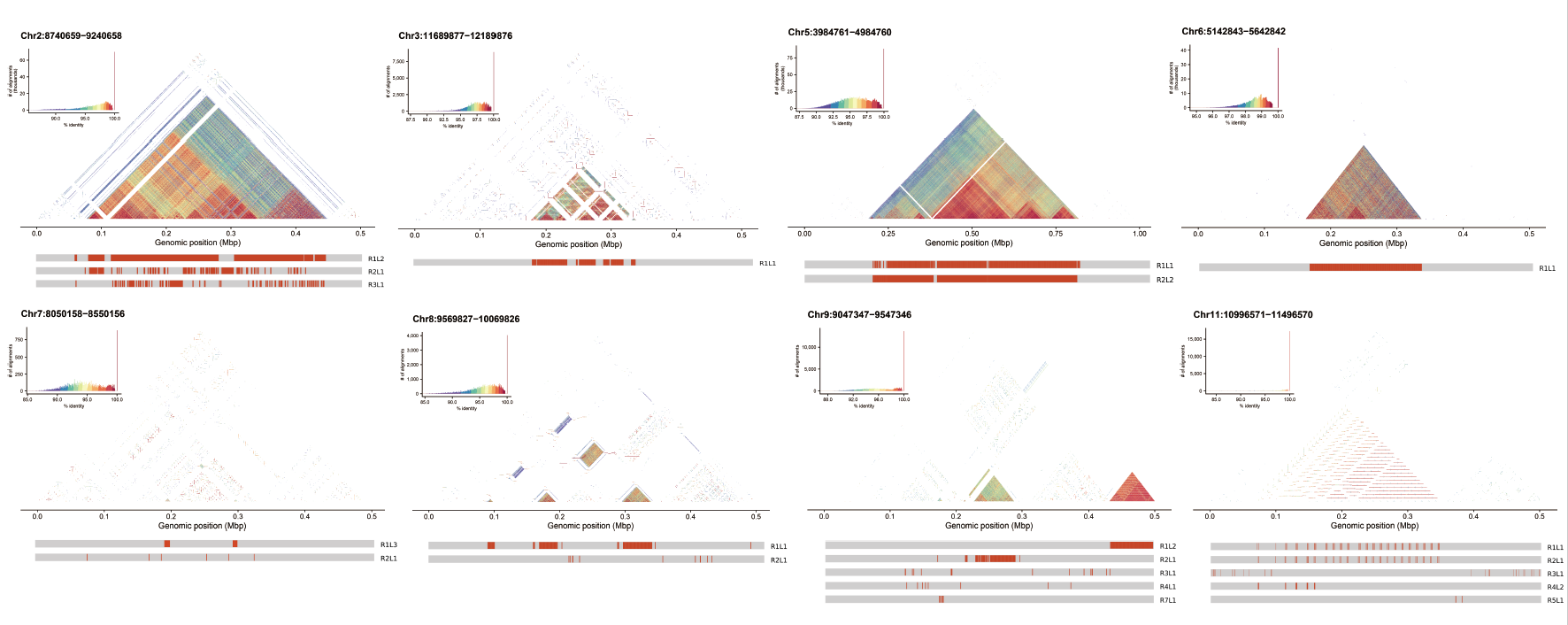


Fig. S3 Stained Glass sequence-identity heatmaps of the centromeres of AVX (A), APZ (B) and AHG (C). Repeat annotations are shown at the bottom of each centromere heatmap


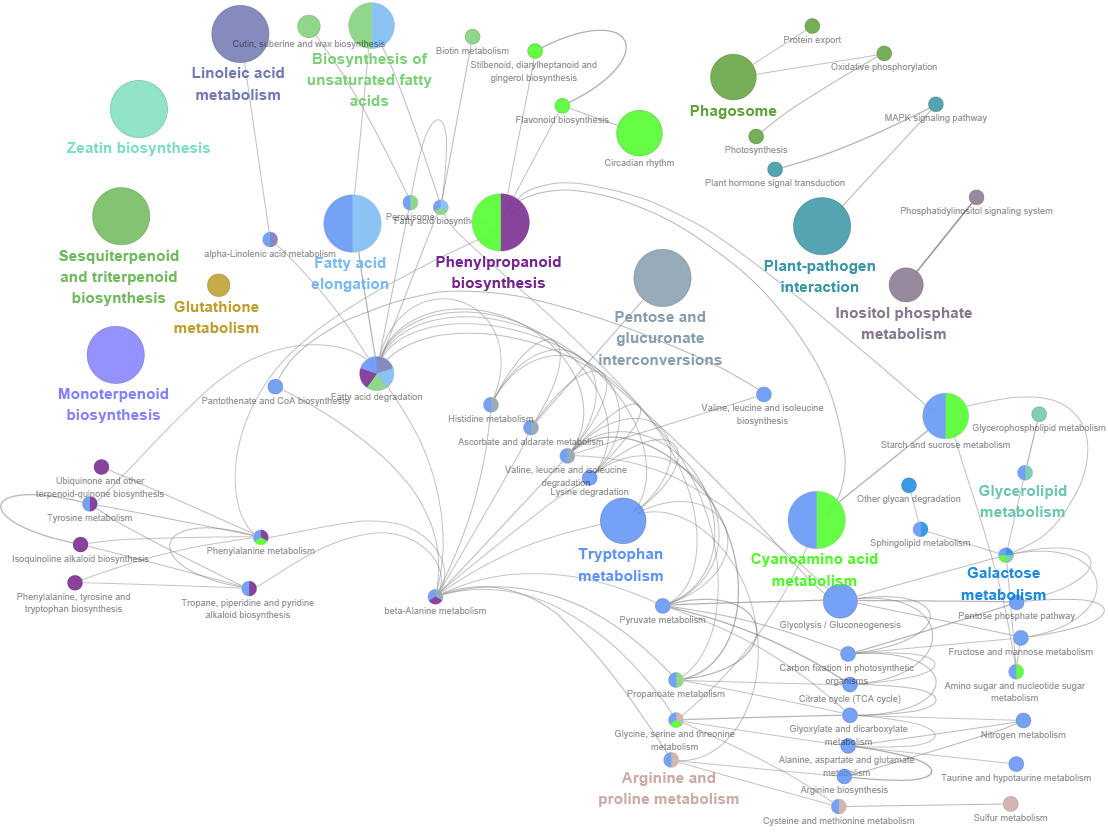


Fig. S4 KEGG enrichment pathways of expanded family genes in *Apocynum* species


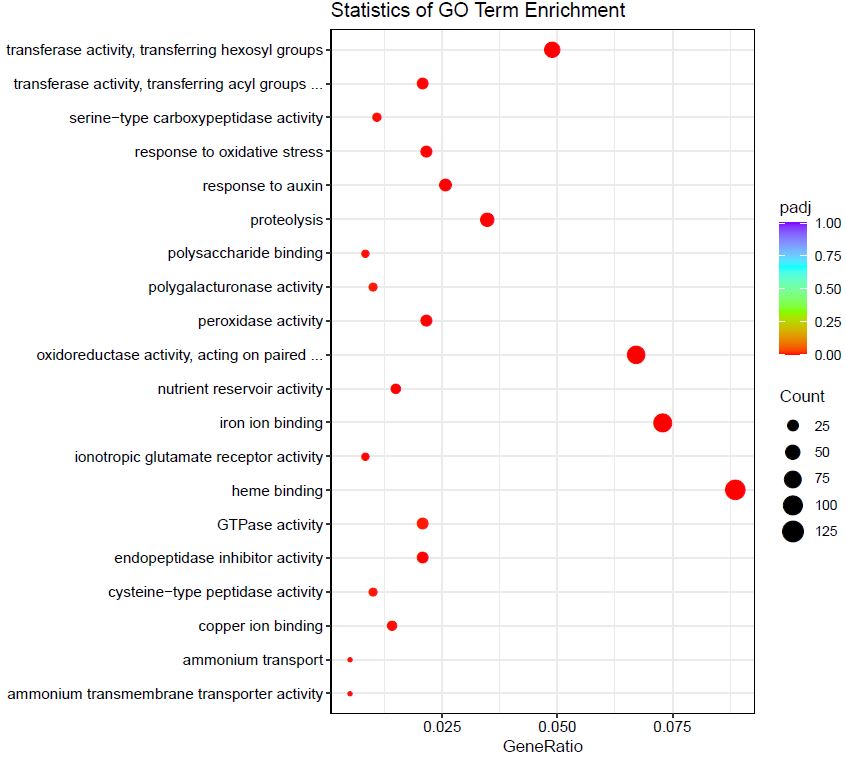


Fig. S5 GO enrichment of expanded family genes in *Apocynum* species


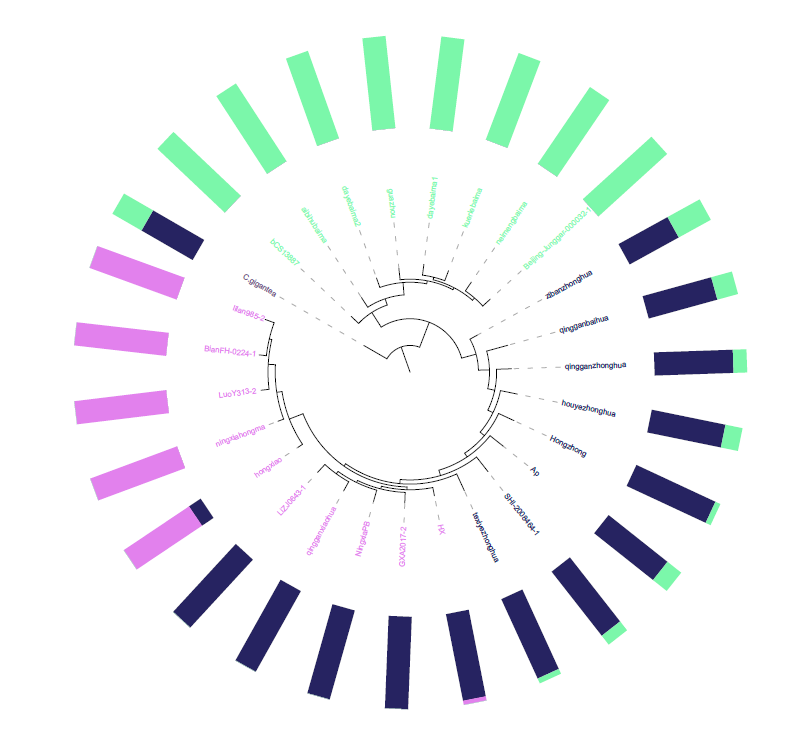


Fig. S6 Phylogenetic tree and principal component for the *Apocynum* accessions using SNPs. APZ as a reference genome.


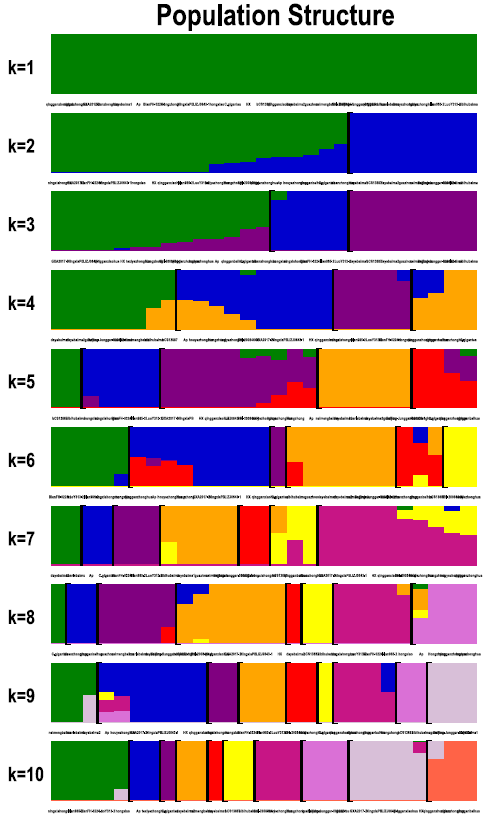


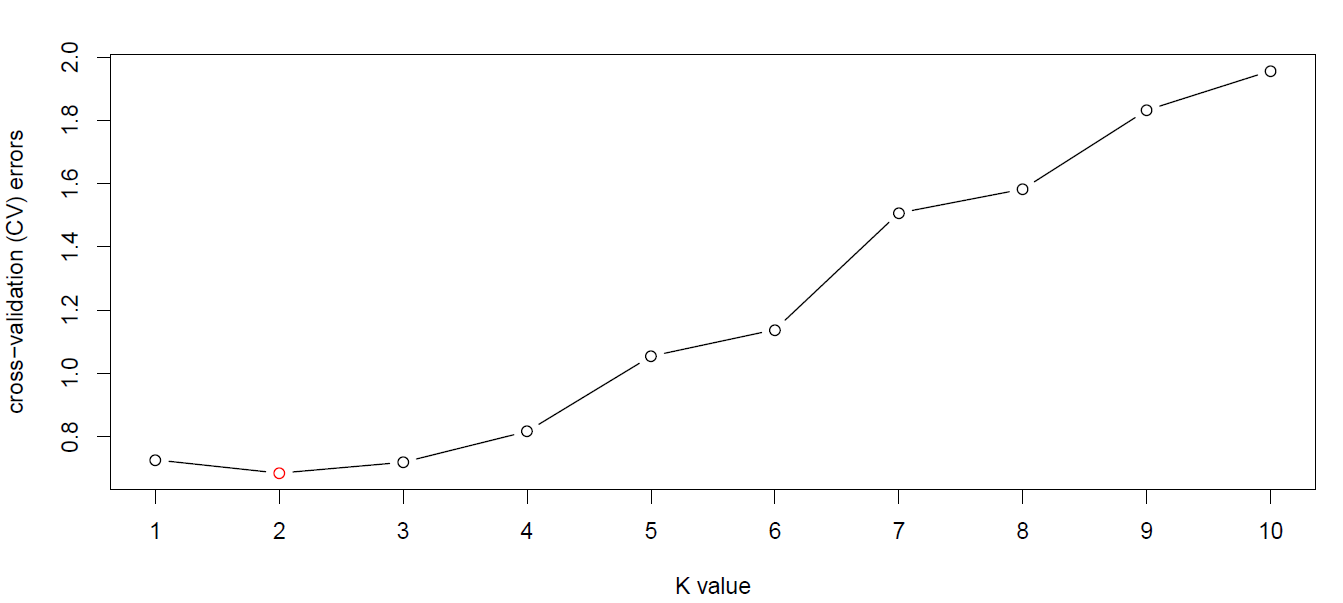


Fig. S7 Model-based analysis of population admixture with K=3


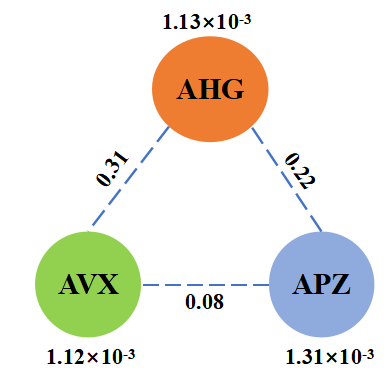


Fig. S8 Genetic diversity (π) and population differentiation (*Fst*) across the three *Apocynum* subpopulations.


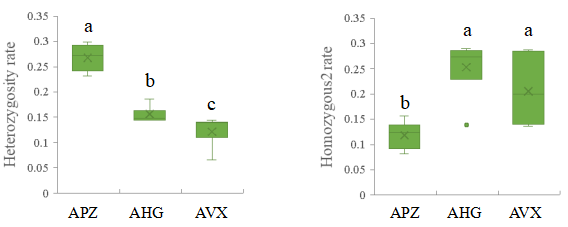


Fig. S9 The heterozygous ratio in APZ, AVX and AHG groups


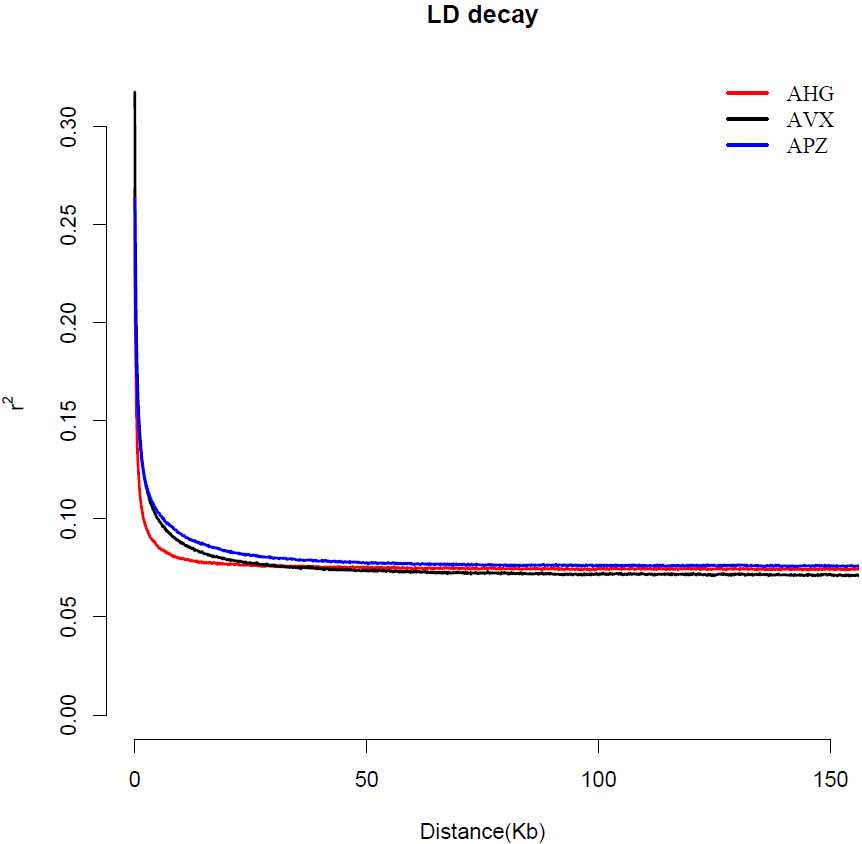


Fig. S10 Decay of linkage disequilibrium of the three *Apocynum* subpopulations


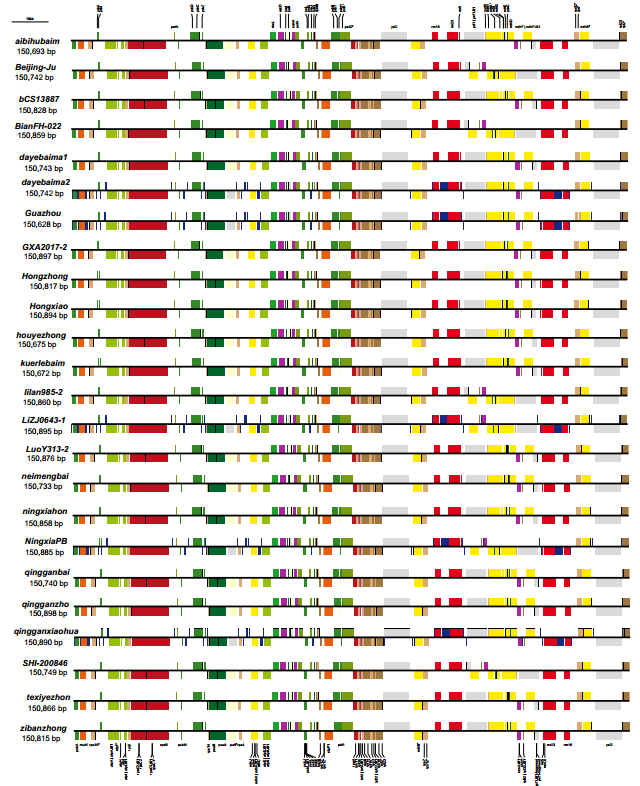


Fig. S11 Gene features of 24 assembled *Apocynum* chloroplast genomes. The genes drawn above the line are on the positive strand, and the genes drawn under the line are located on the native strand.

A B


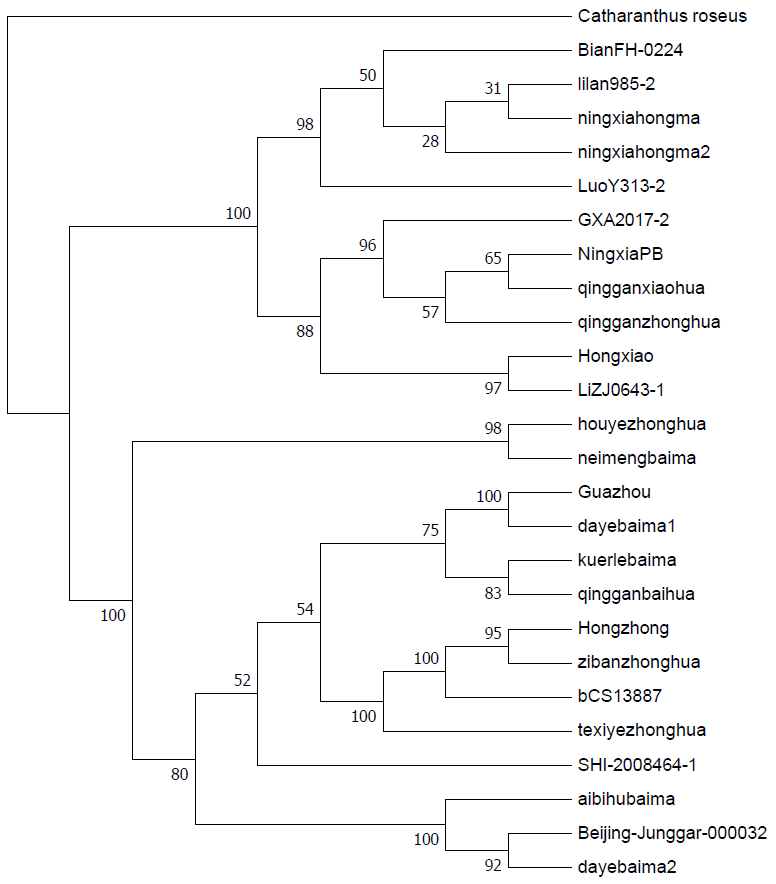

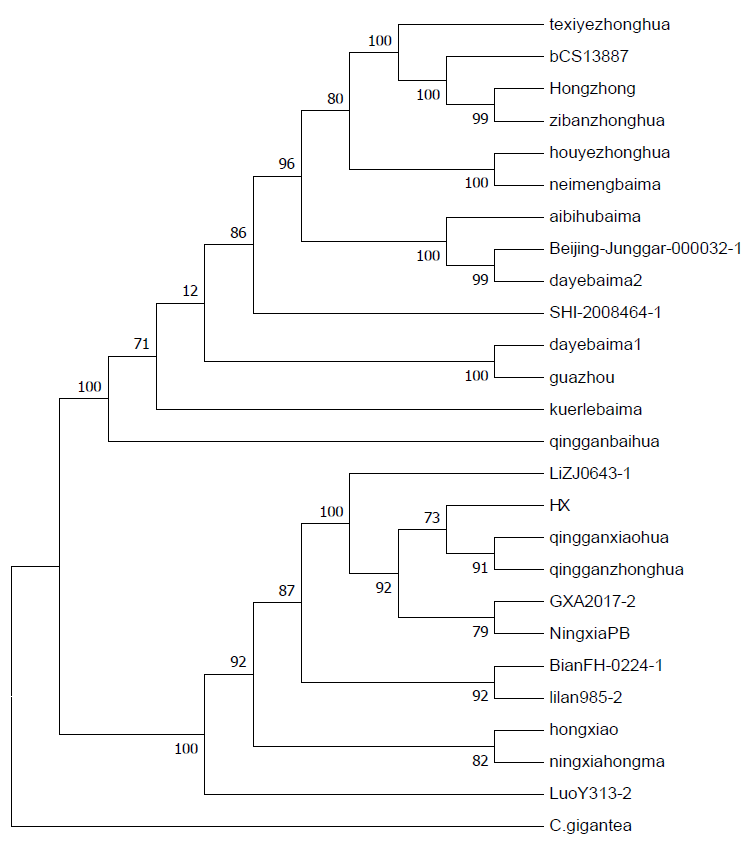


C


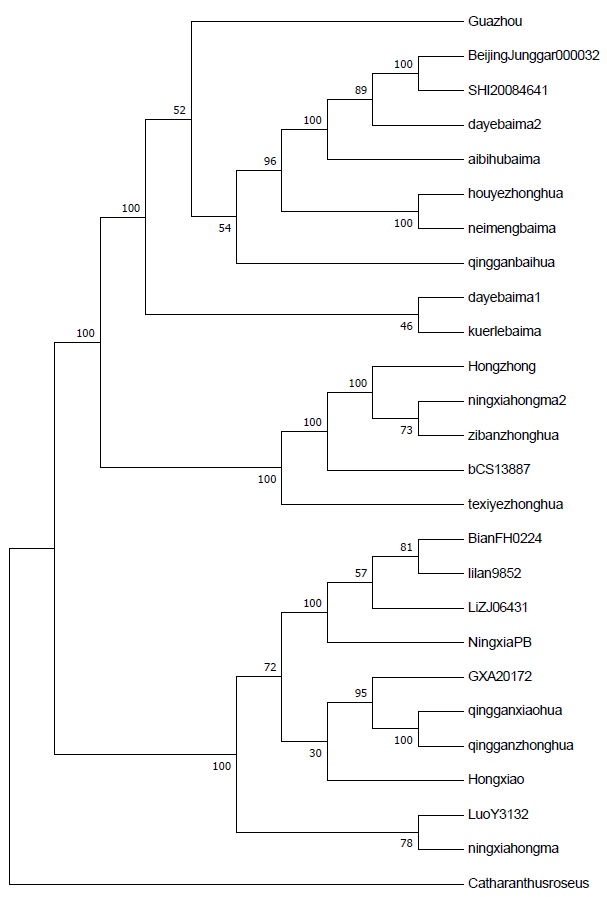


Fig. S12 Phylogenetic tree constructed based on the CDS of the chloroplast genome (A), Phylogenetic tree constructed based on the snp of the chloroplast genome (B), Phylogenetic tree constructed by chloroplast genome sequence of species use IQTREE (C).


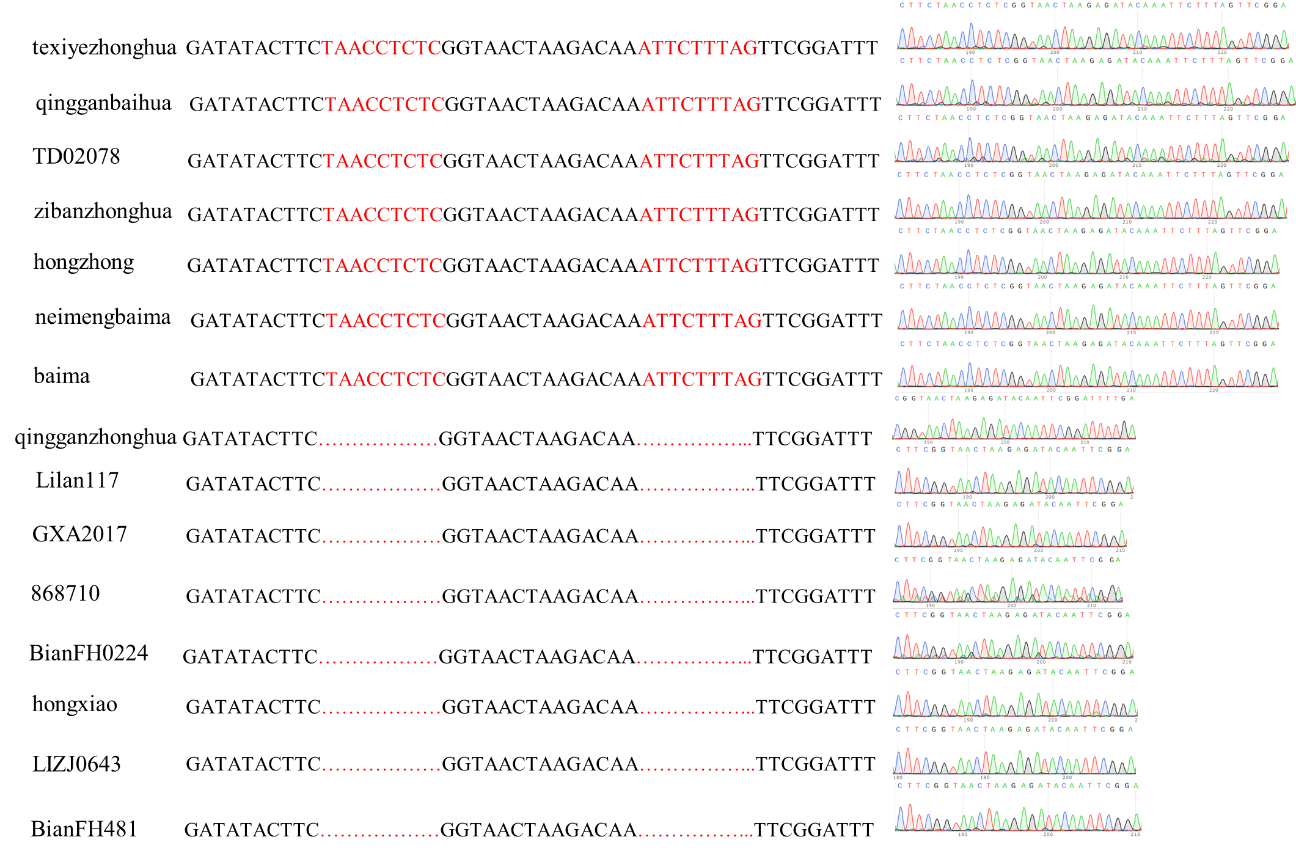


Fig. S13 The *rpoC2* with 9-bp and 8-bp deletions in AVX group


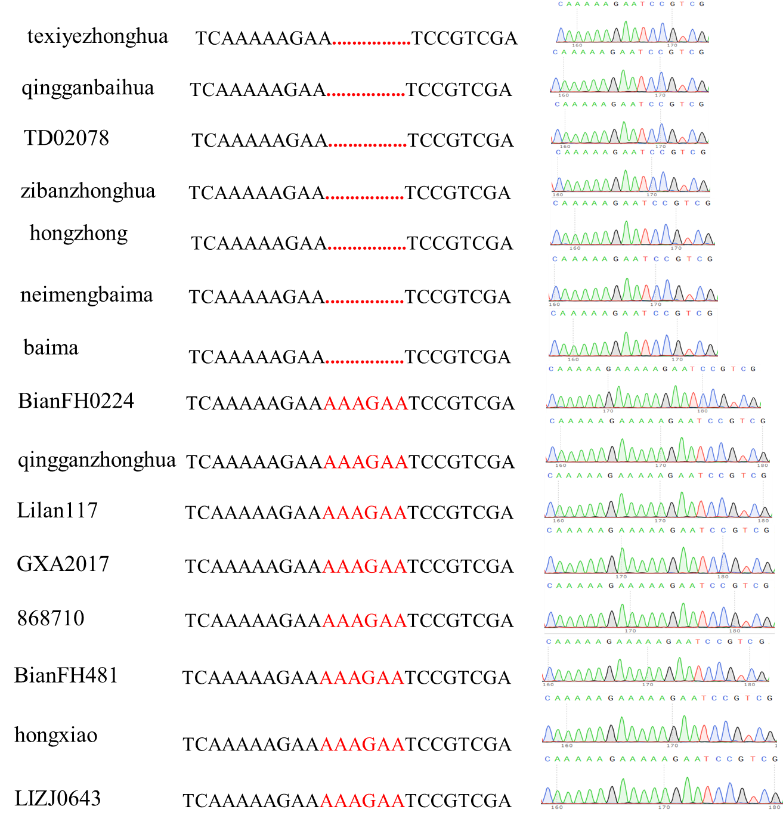


Fig. S14 The *ycf1* with 5-bp insertion in AVX group


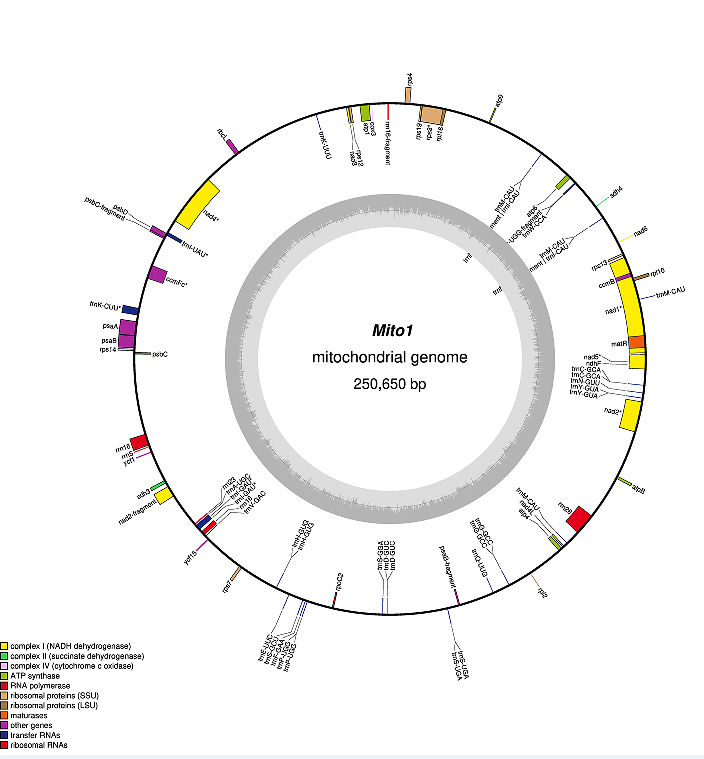

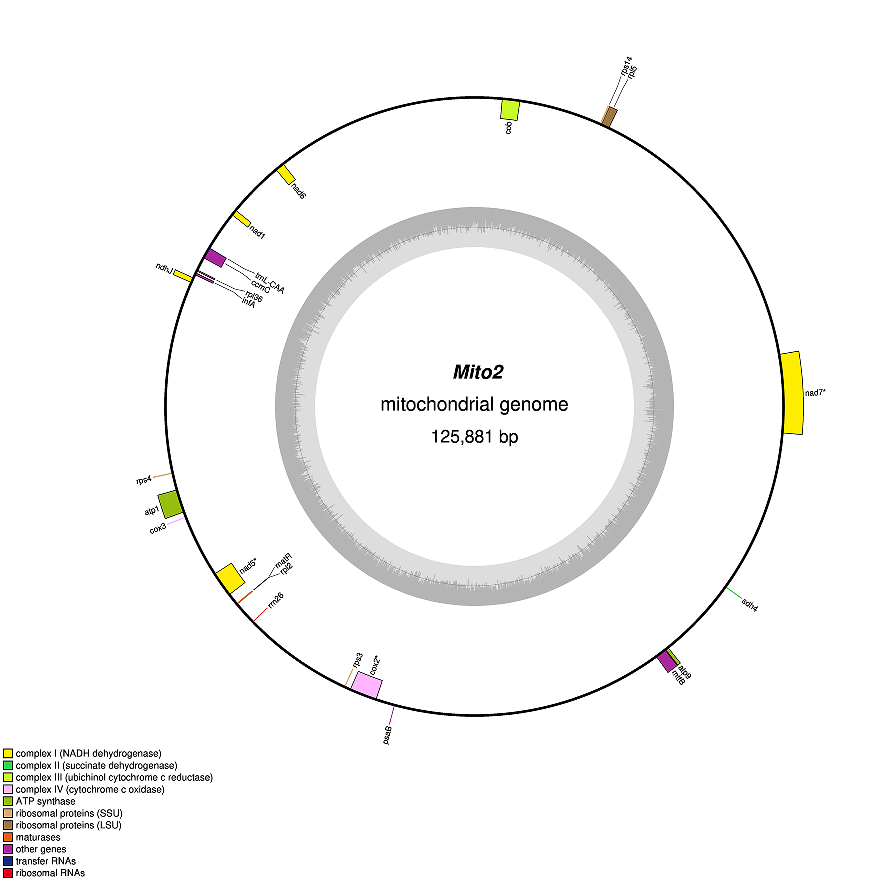


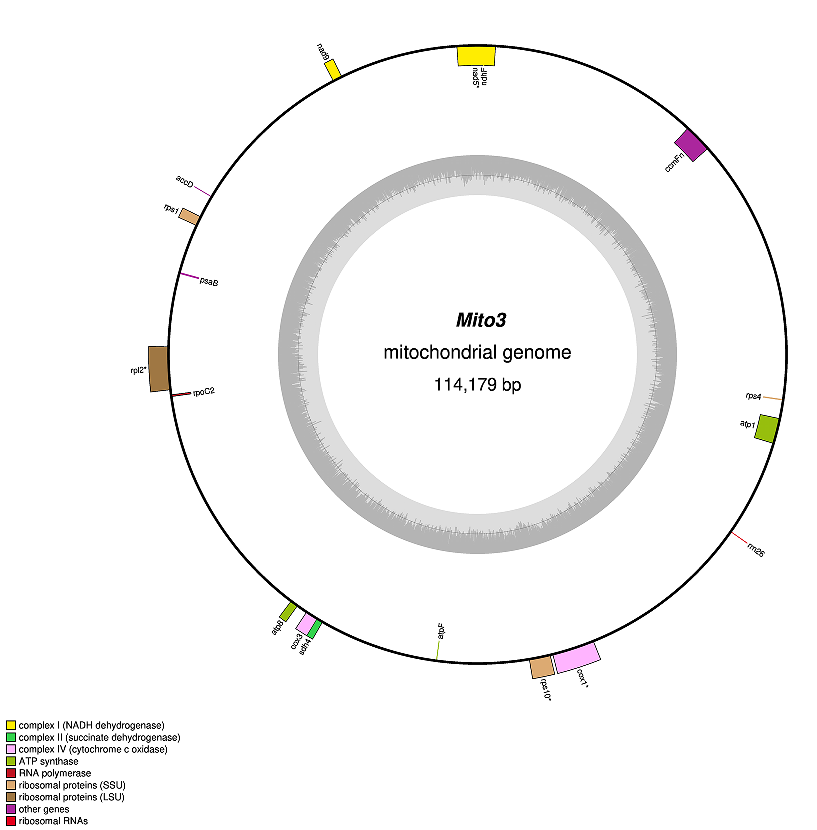


Fig. S15 The Circular gene map of the APZ mitochondrial genome.

A B


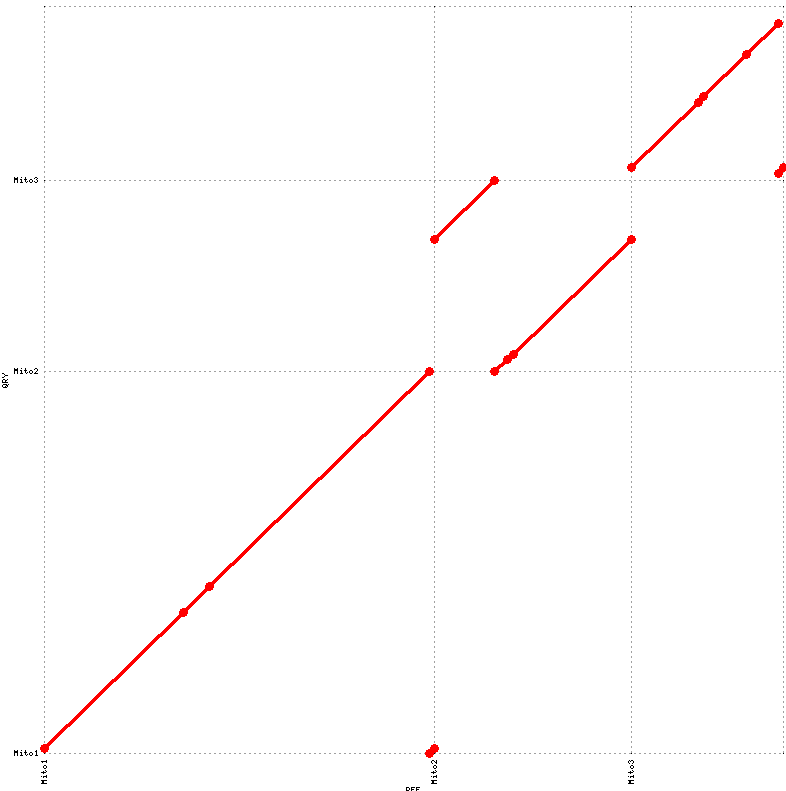

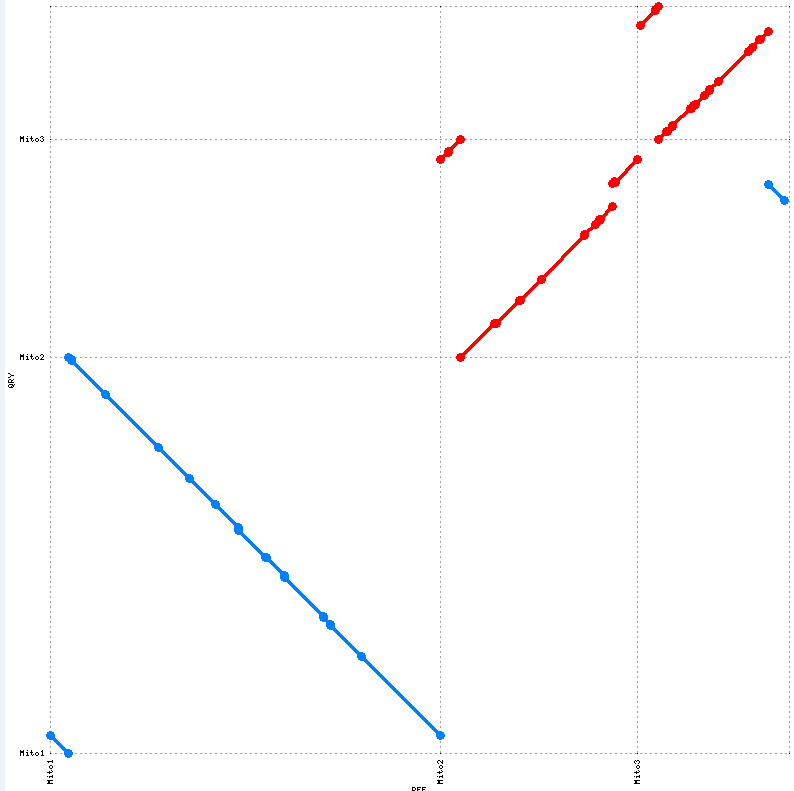


C


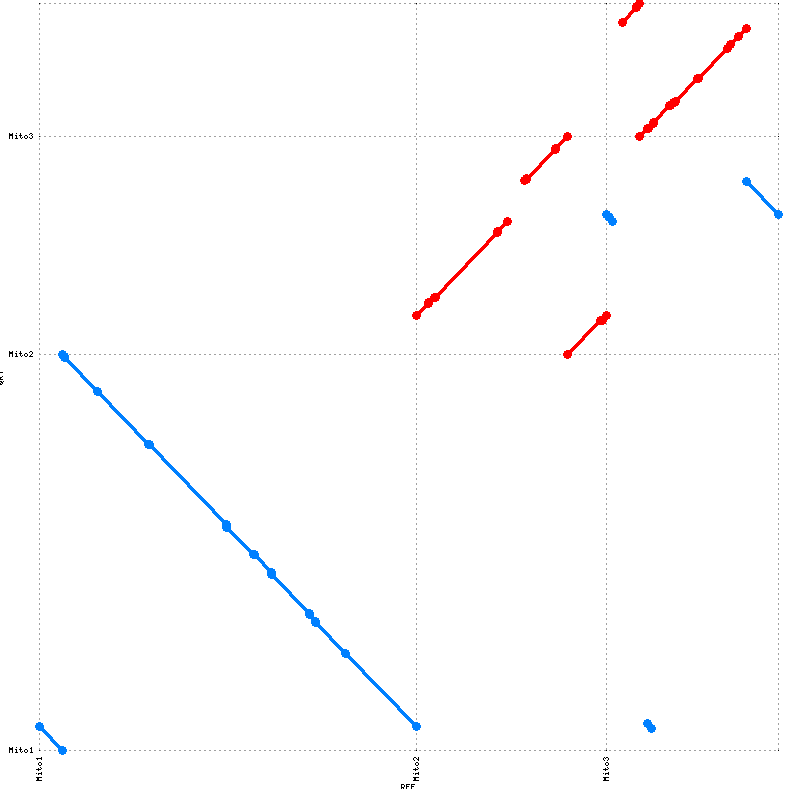


Fig. S16 The mitochondrial genome collinearity in *Apocynum*. (A) AVX-vs-AHG; (B) AHG-vs- APZ; (C) AVX-vs-APZ.


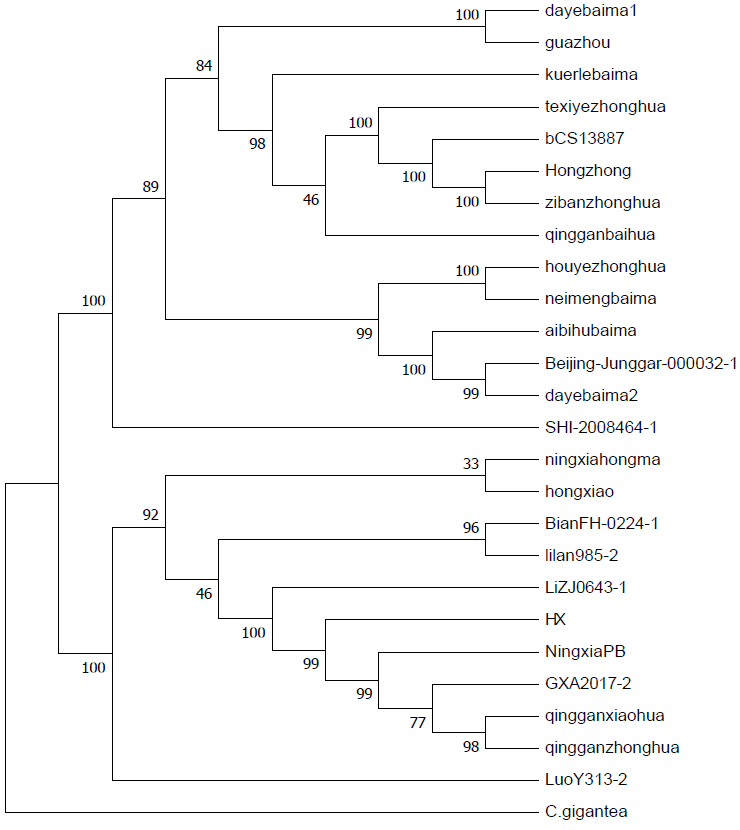


Fig. S17 Phylogenetic tree constructed based on the snp of the chloroplast and mitochondrial genome in *Apocynum*

A
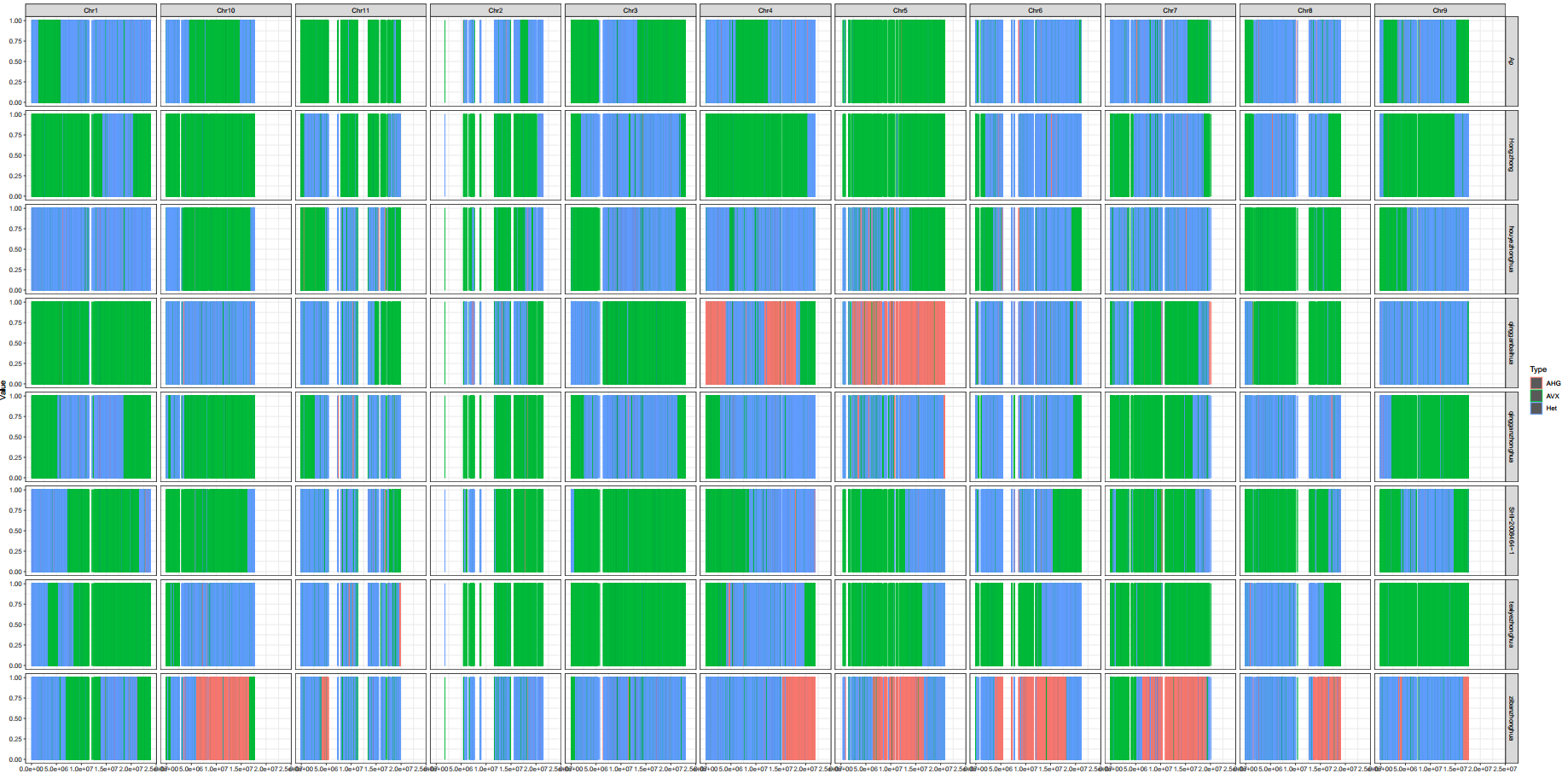


B


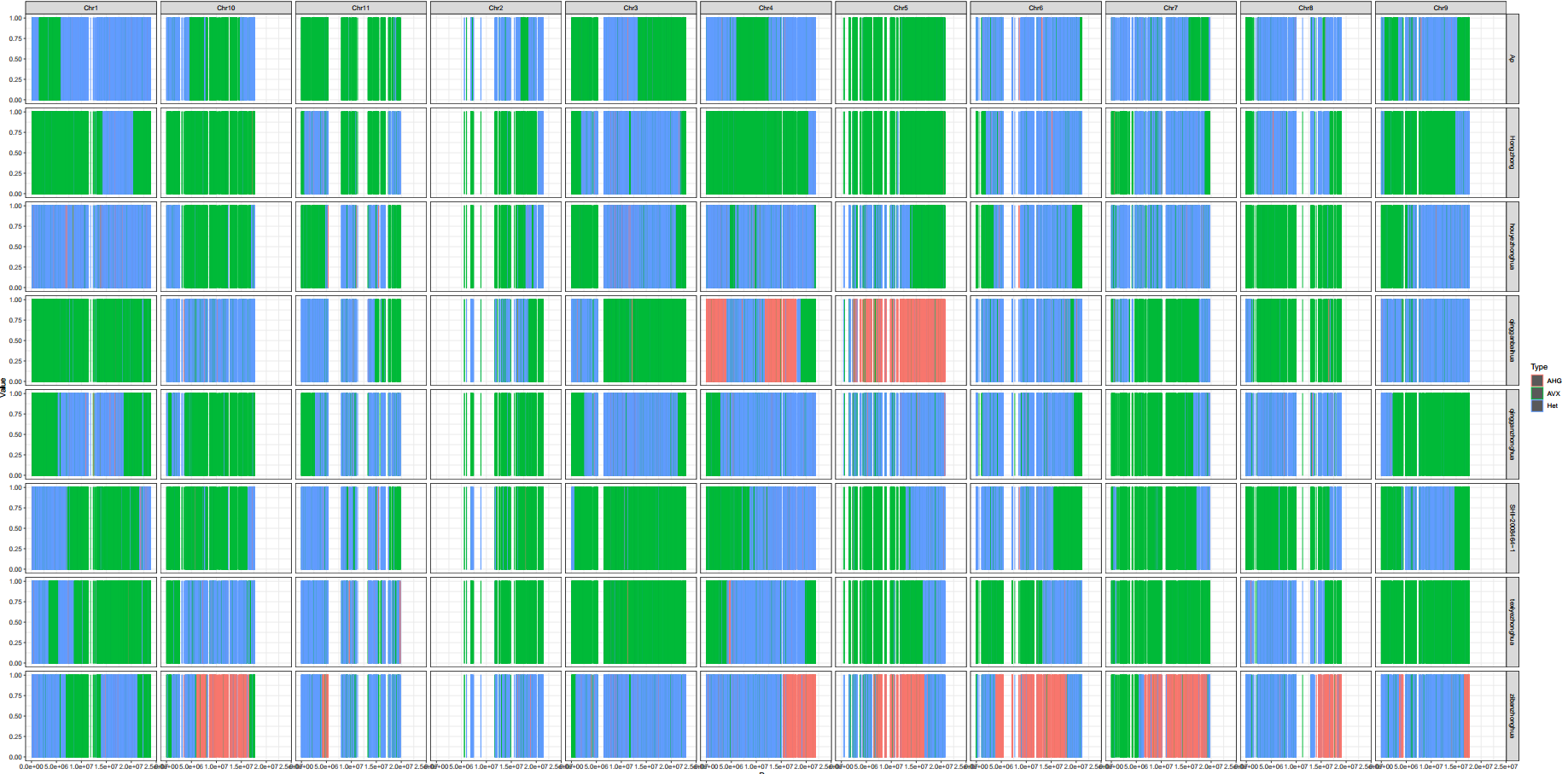


C


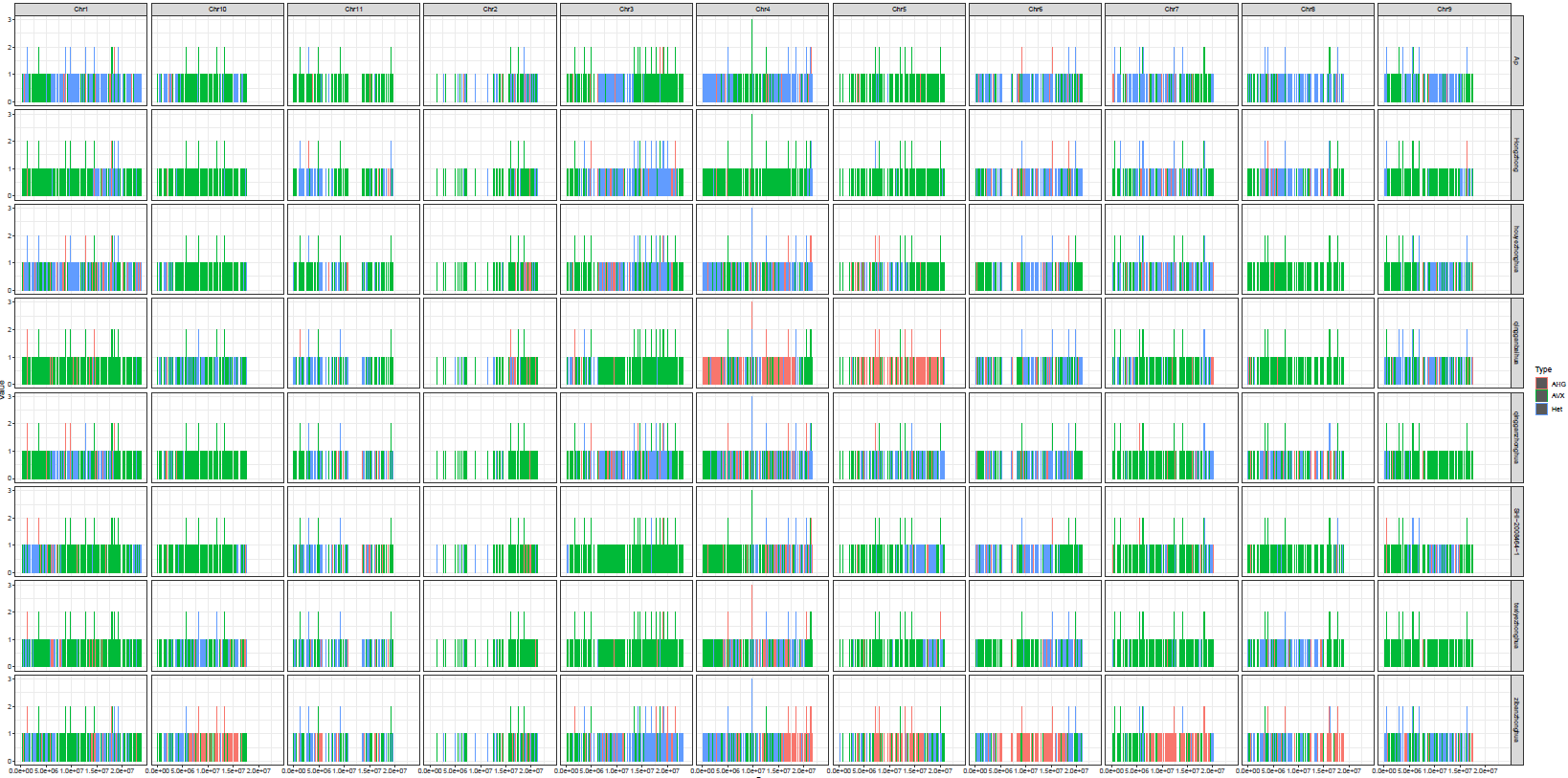


Fig. S18 The comprehensive variation landscape for APZ group (A, SNP; B, Indel; C, SV )

A


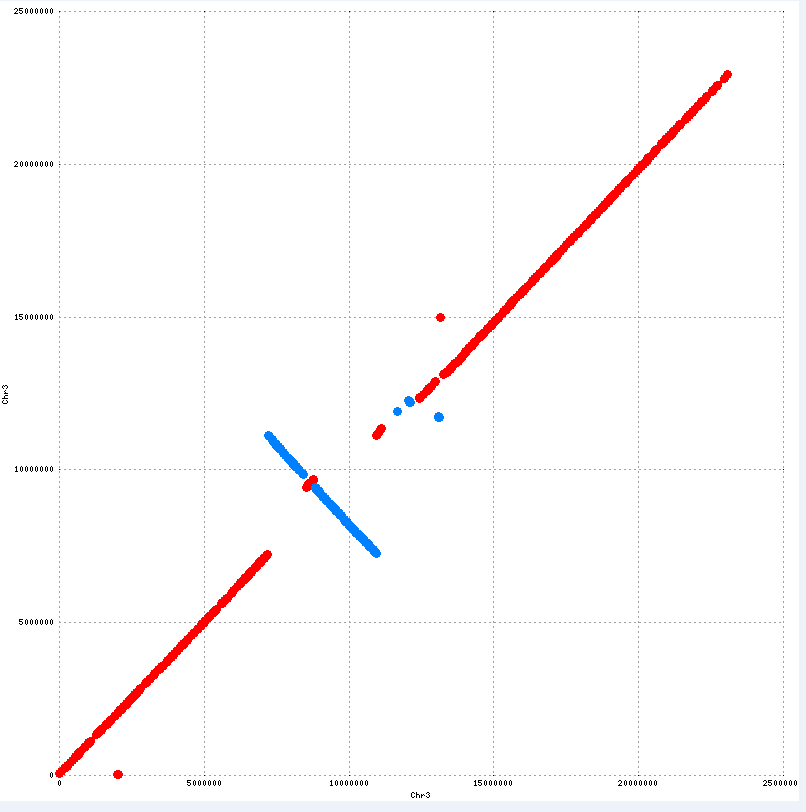

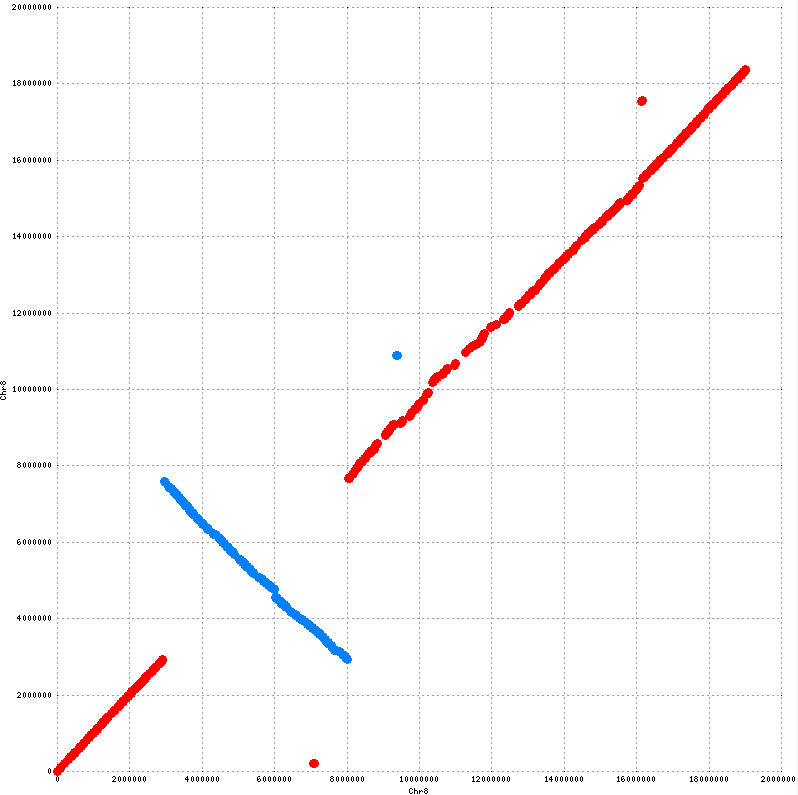


B


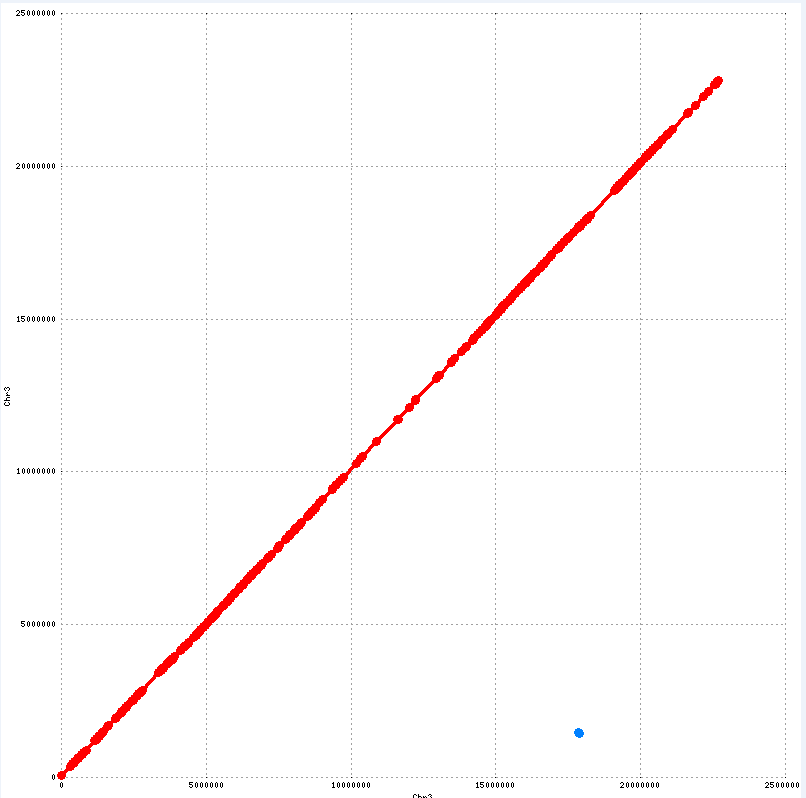

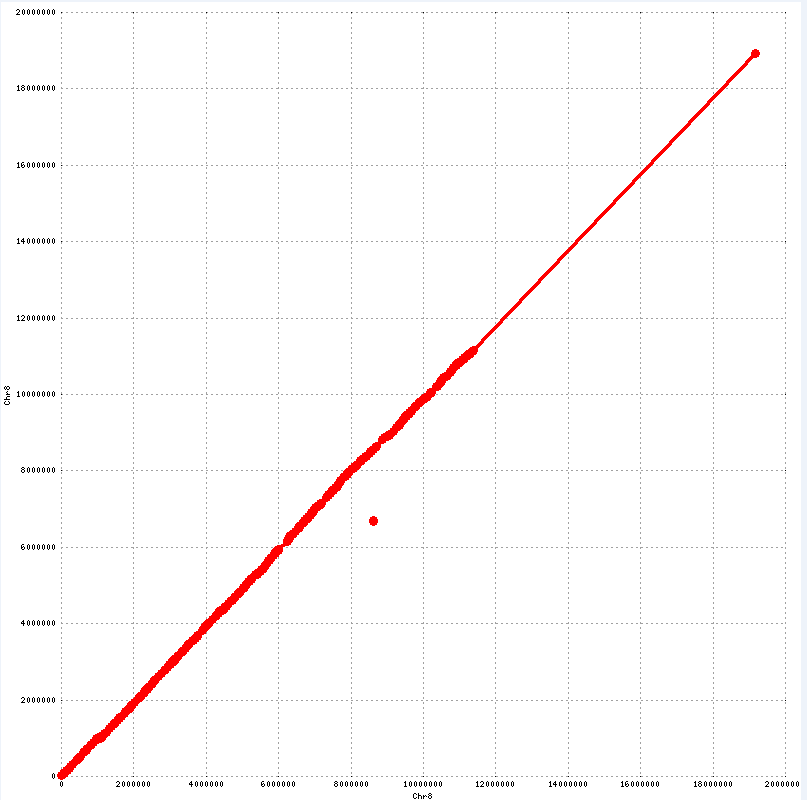


C


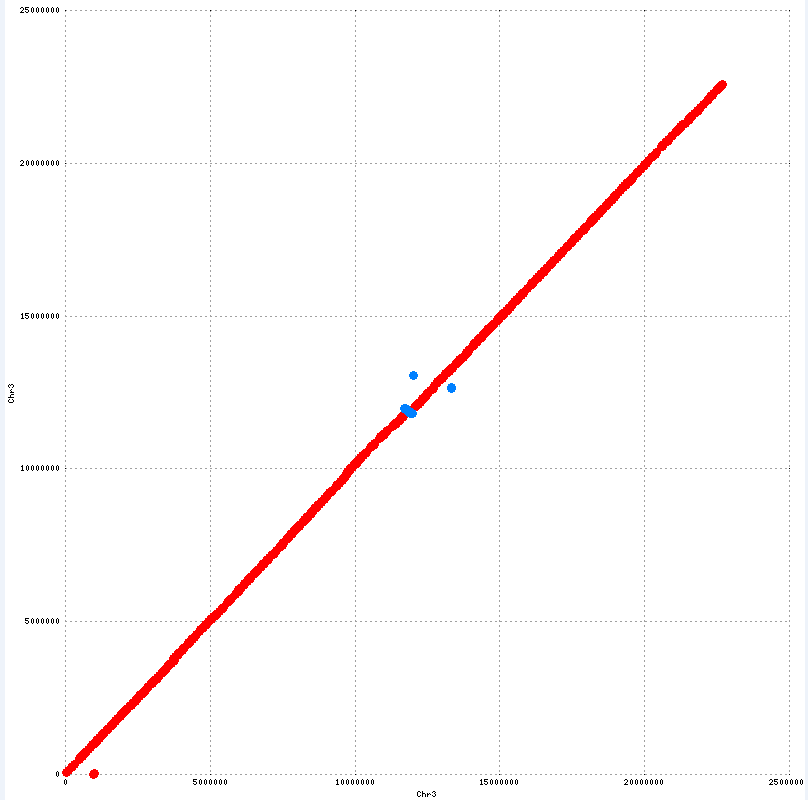

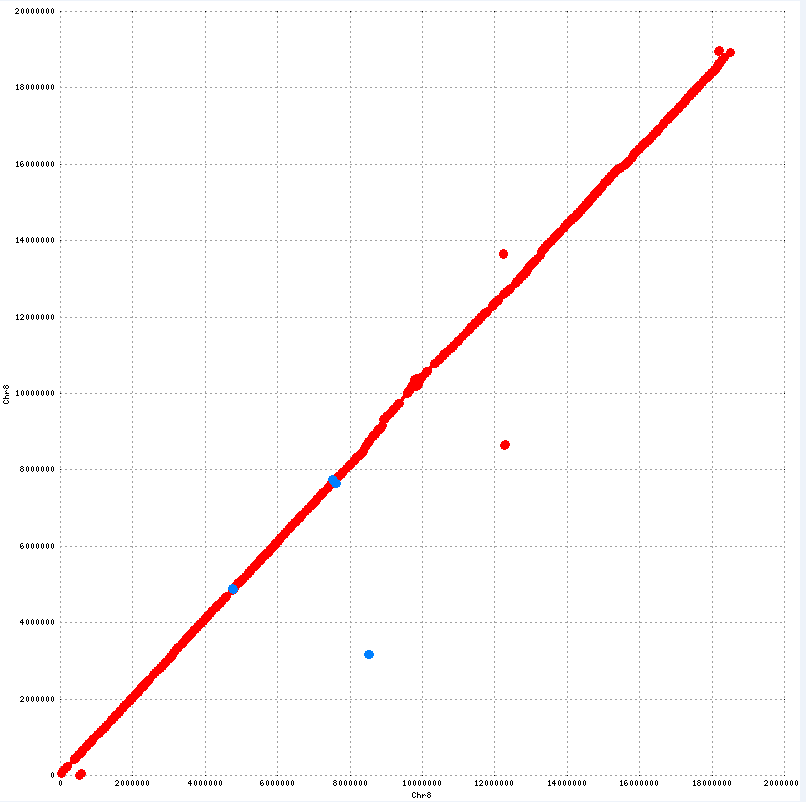


Fig. S19 The syntenic analysis of Chr3 (left) and Chr5 (right) between two haplotypes in *Apocynum*. (A, APZ; B, AVX; C, AHG)

A


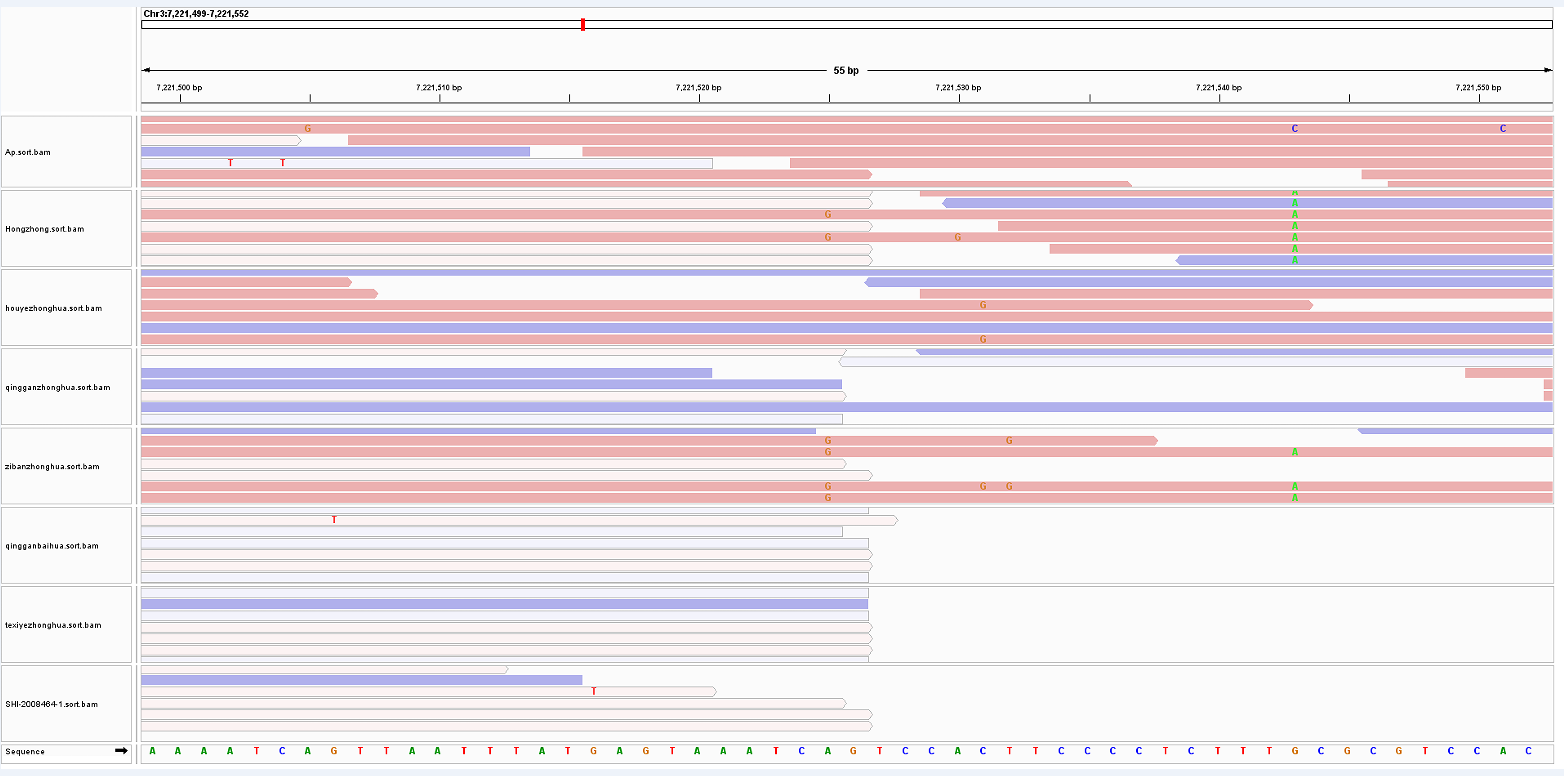


B


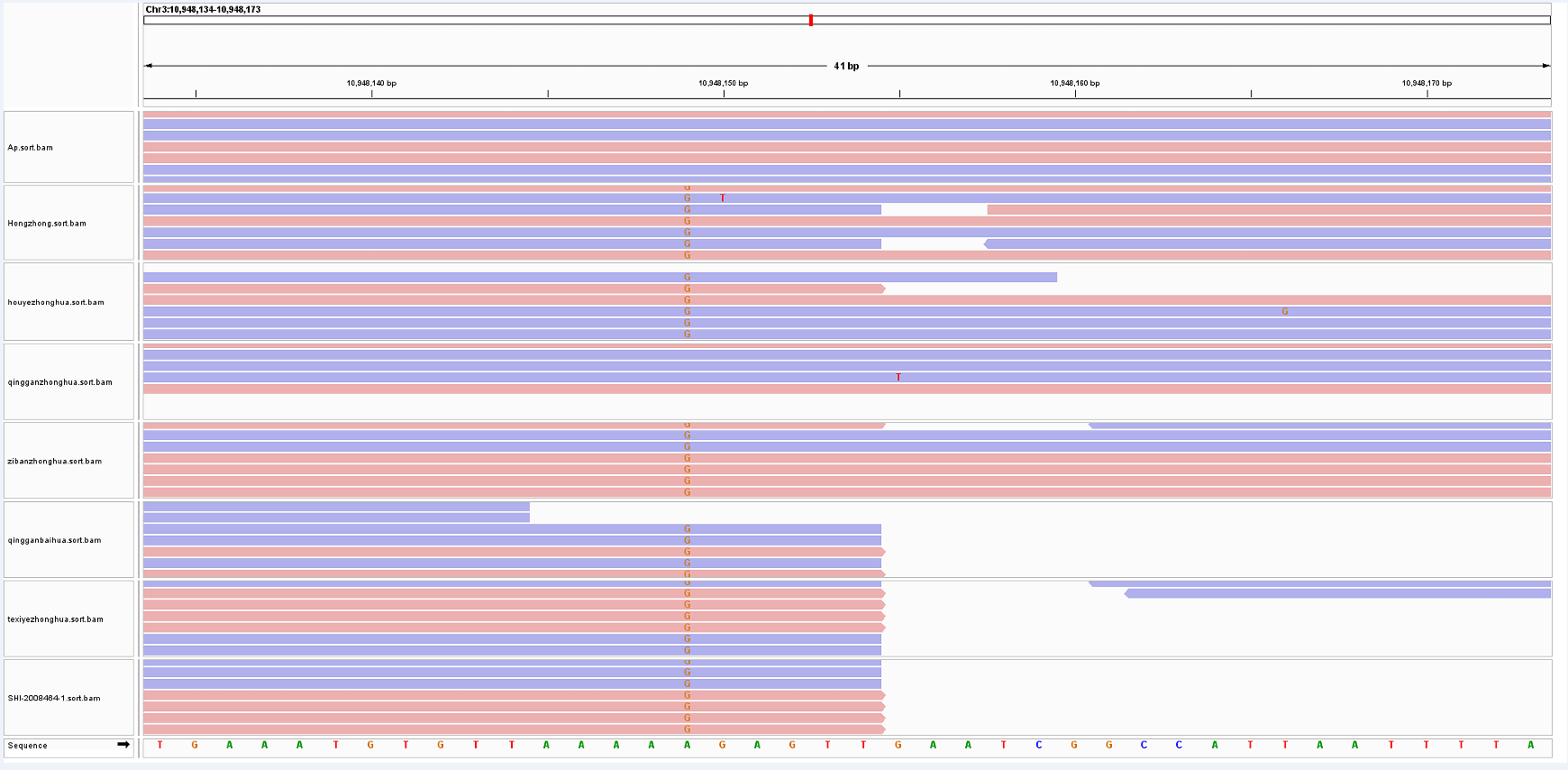


C


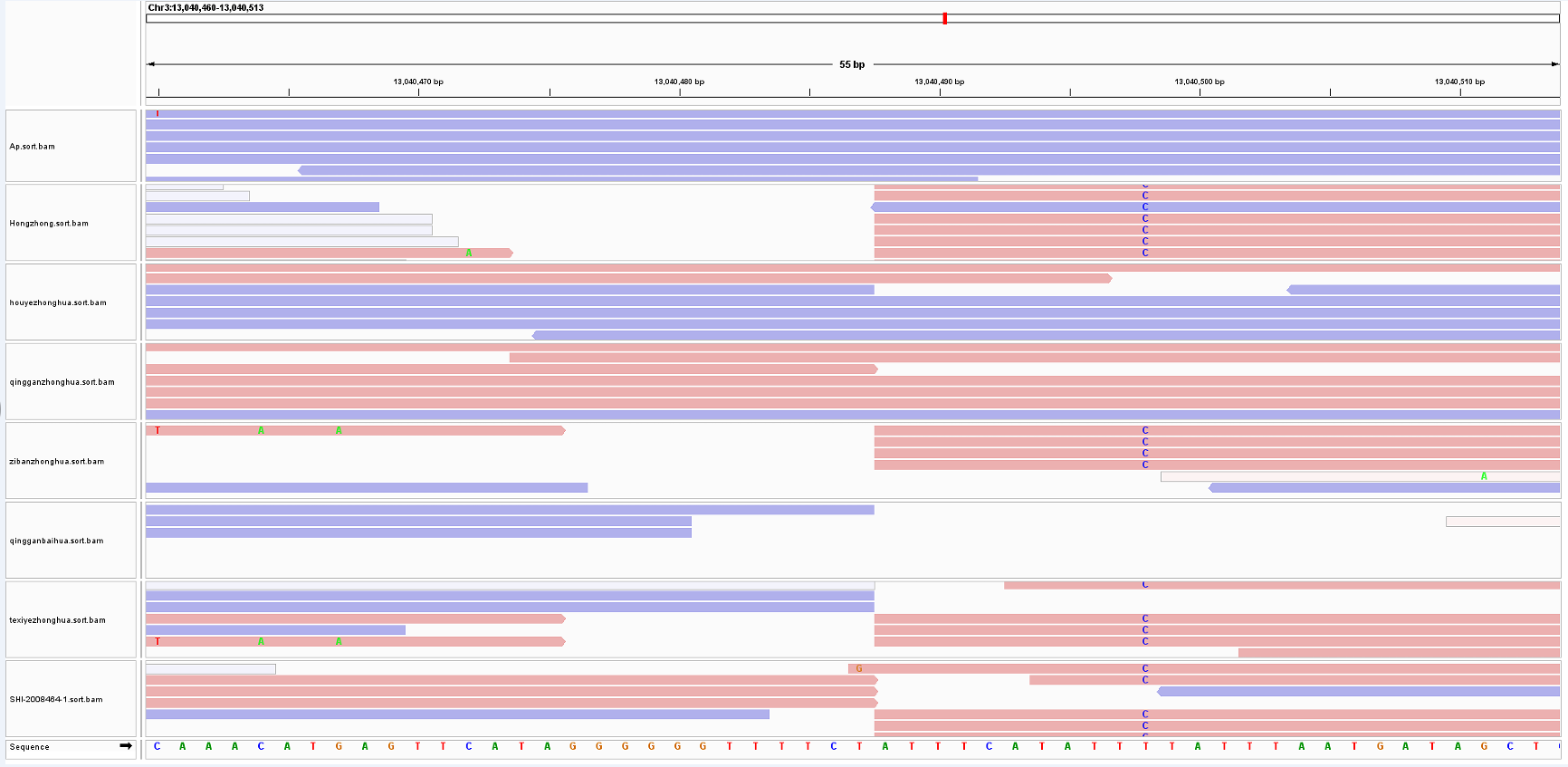


D


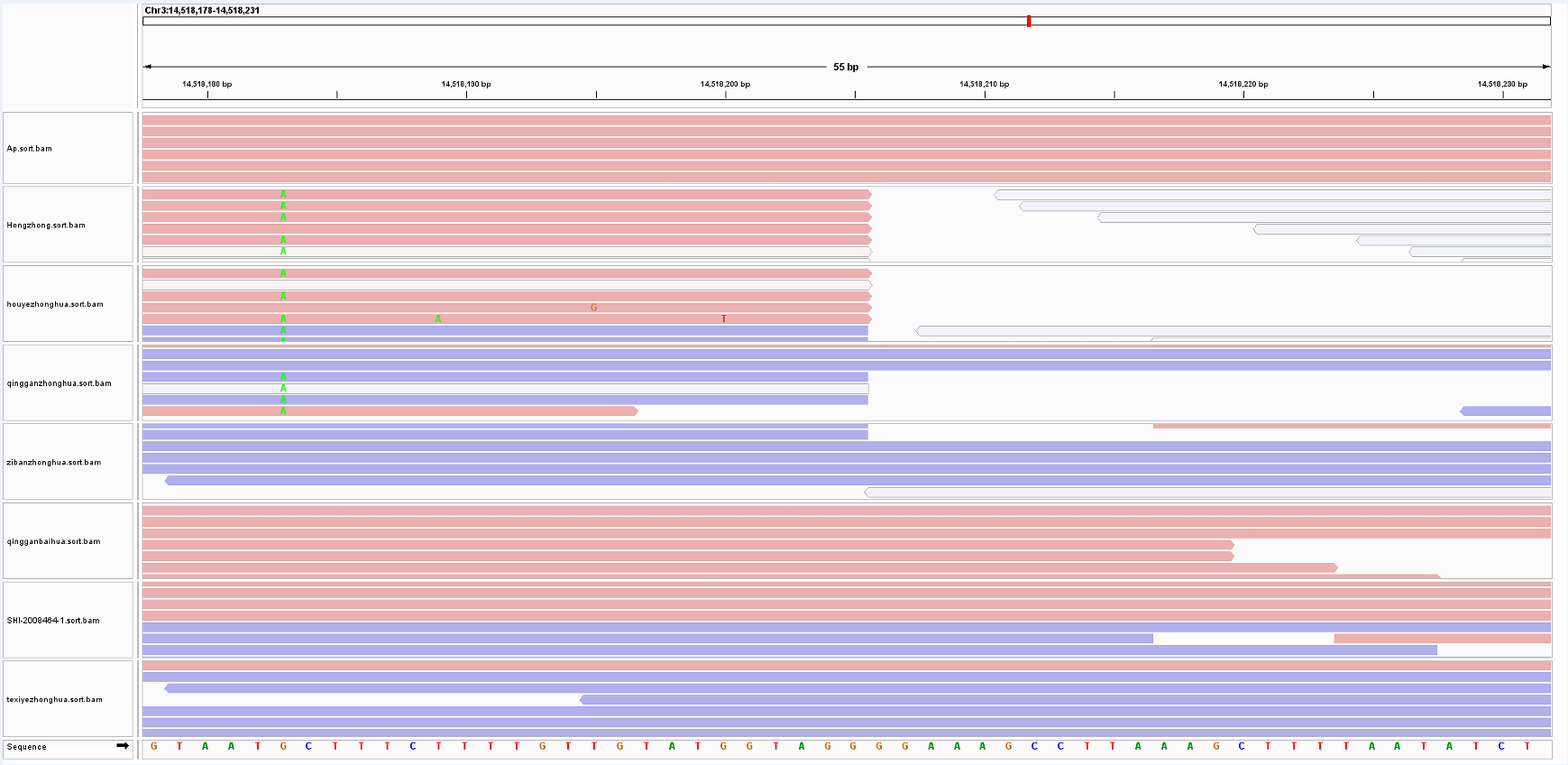


E


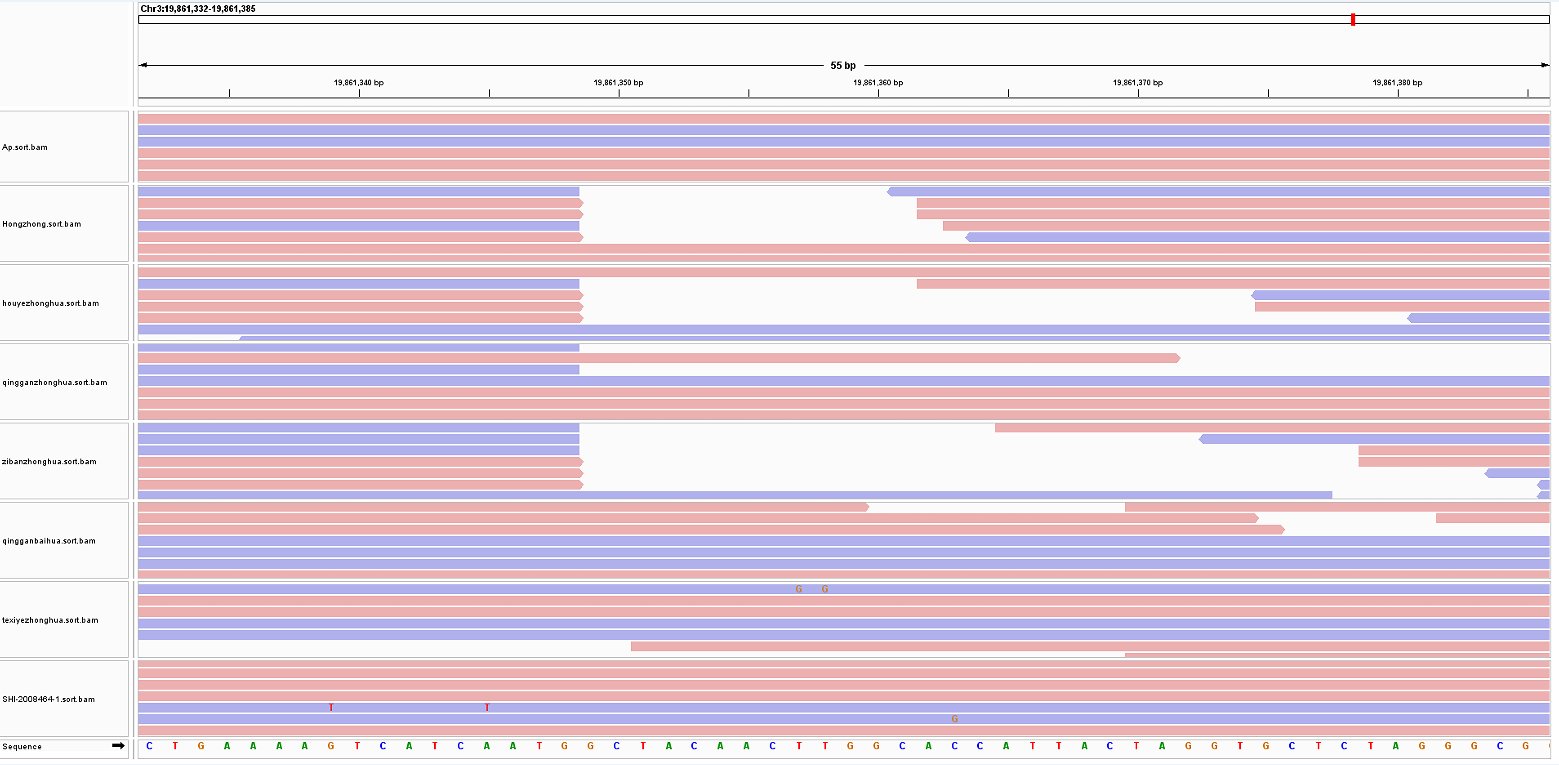


F
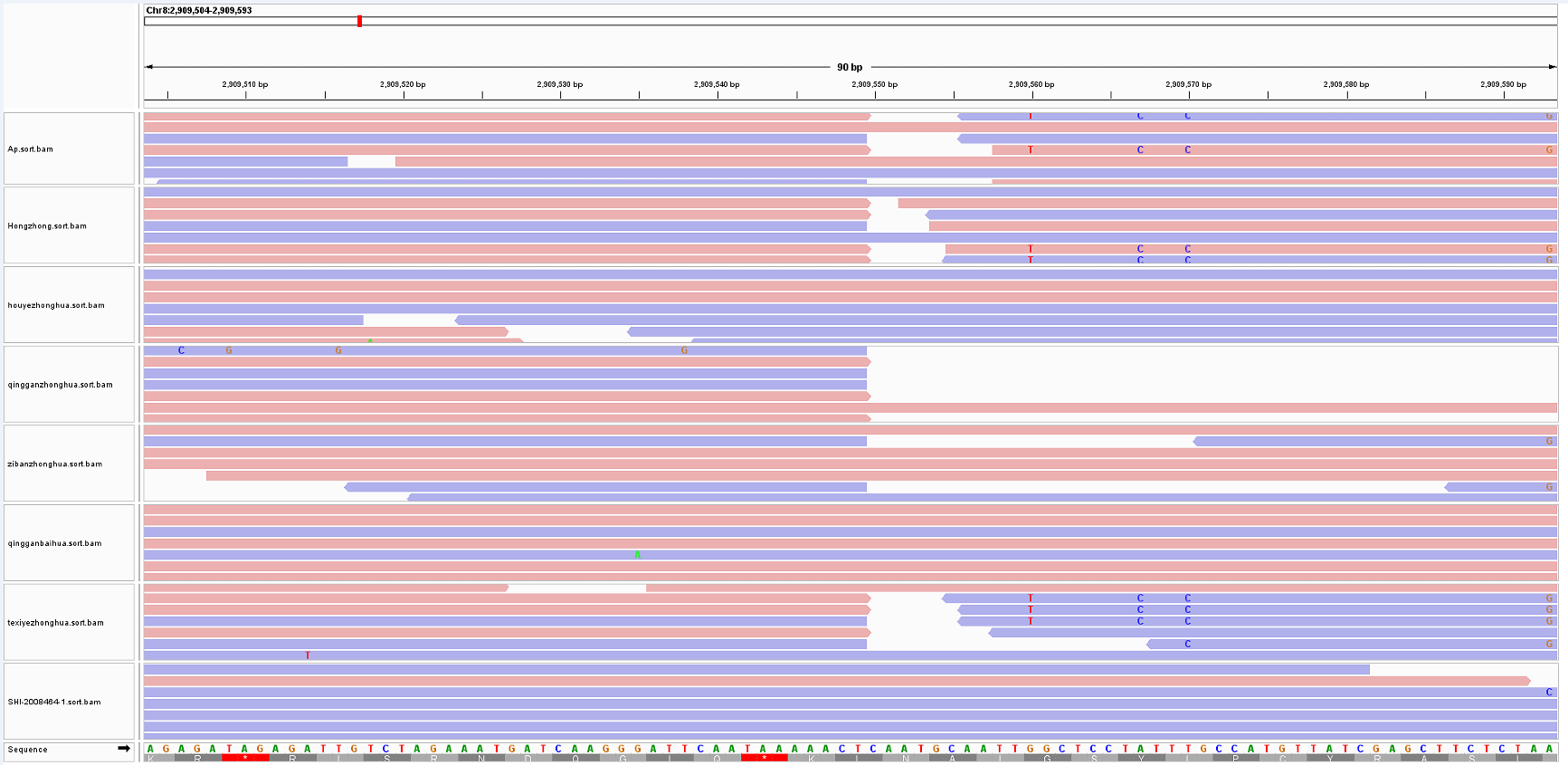


G


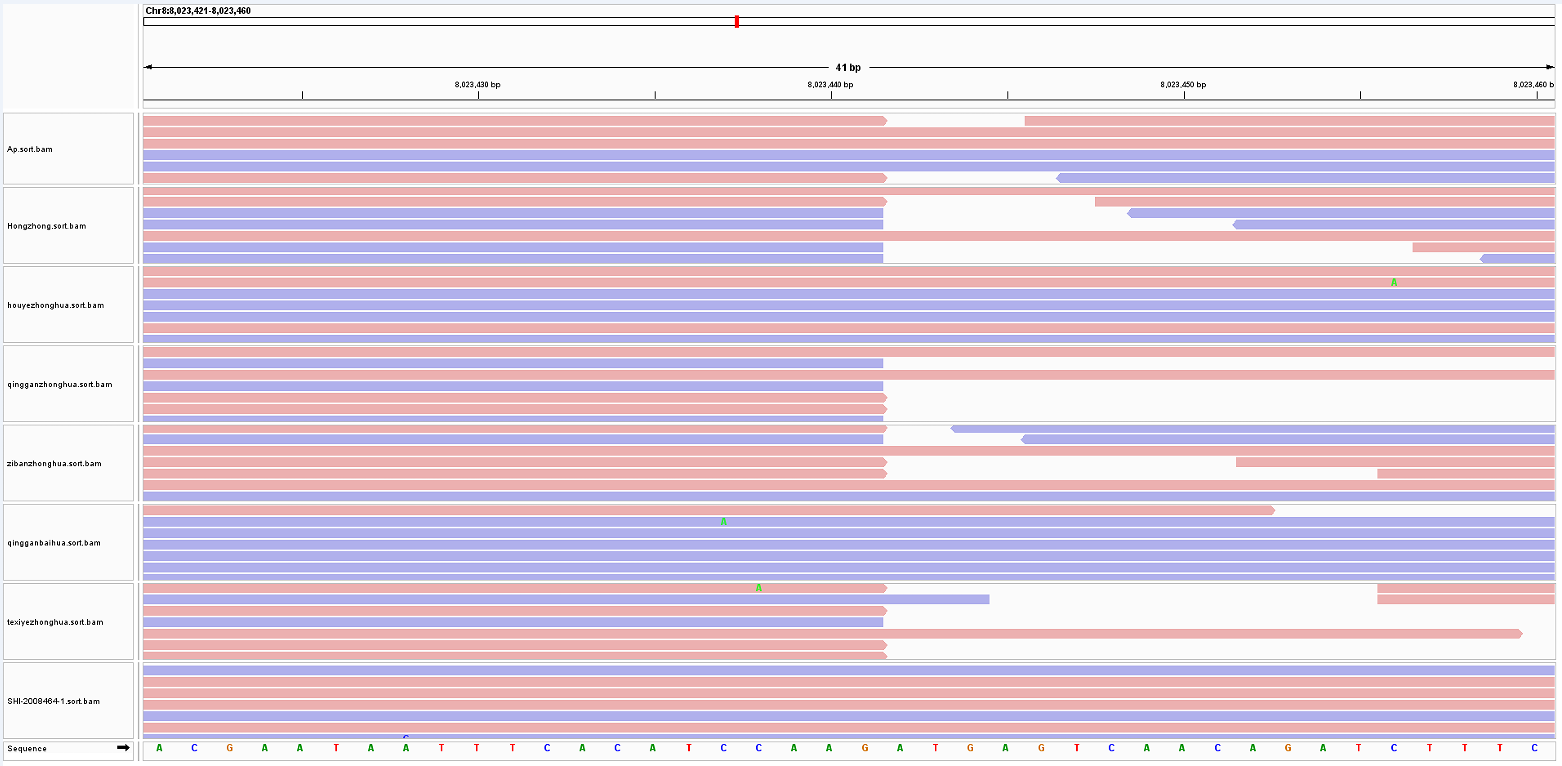


Fig. S20 The inversion points of Chr3 and Chr8 by mapping the raw sequencing reads and visualized by IGV in APZ groups. A, Chr3:7221526; B, Chr3:10948154; C, Chr3:13040487; D, Chr3:13040487; E, Chr3:19861359; F, Chr8:2909549; G, Chr8:8023441.

A


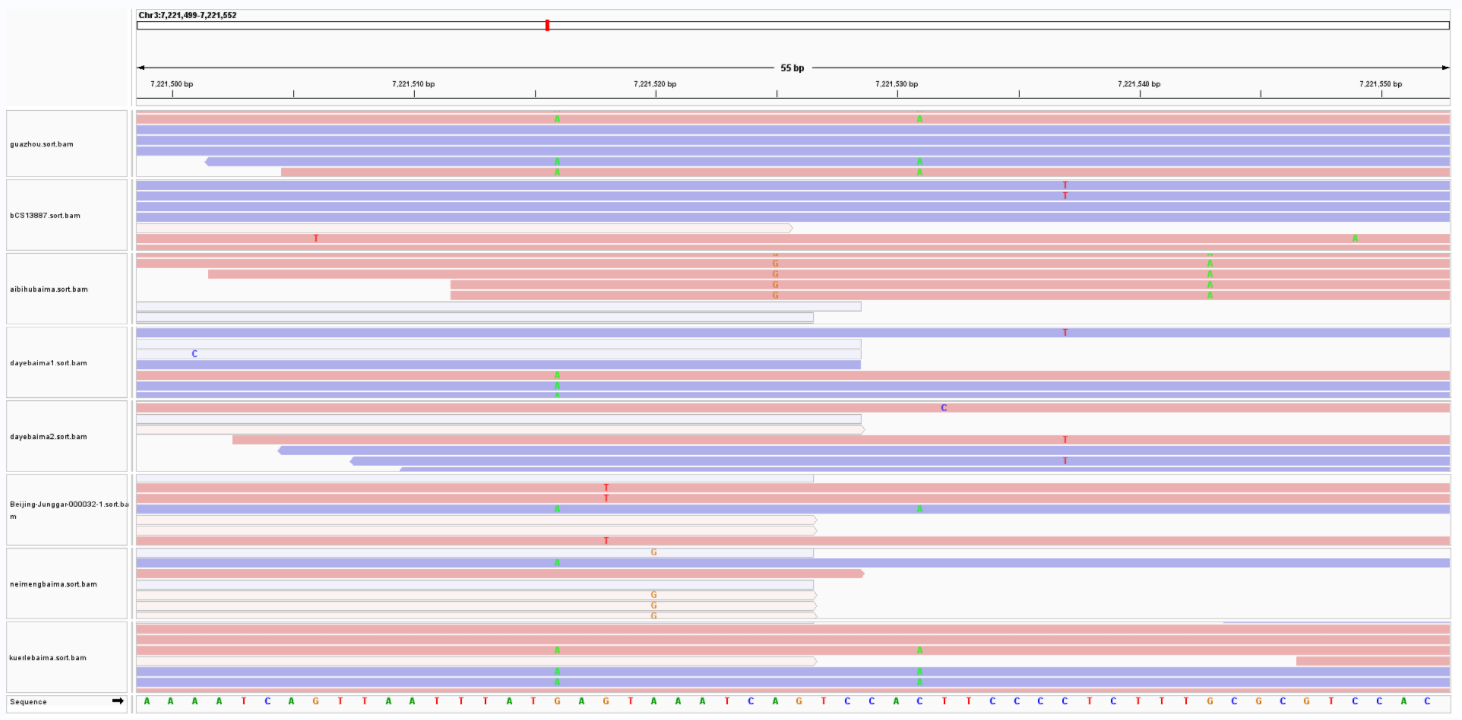


B


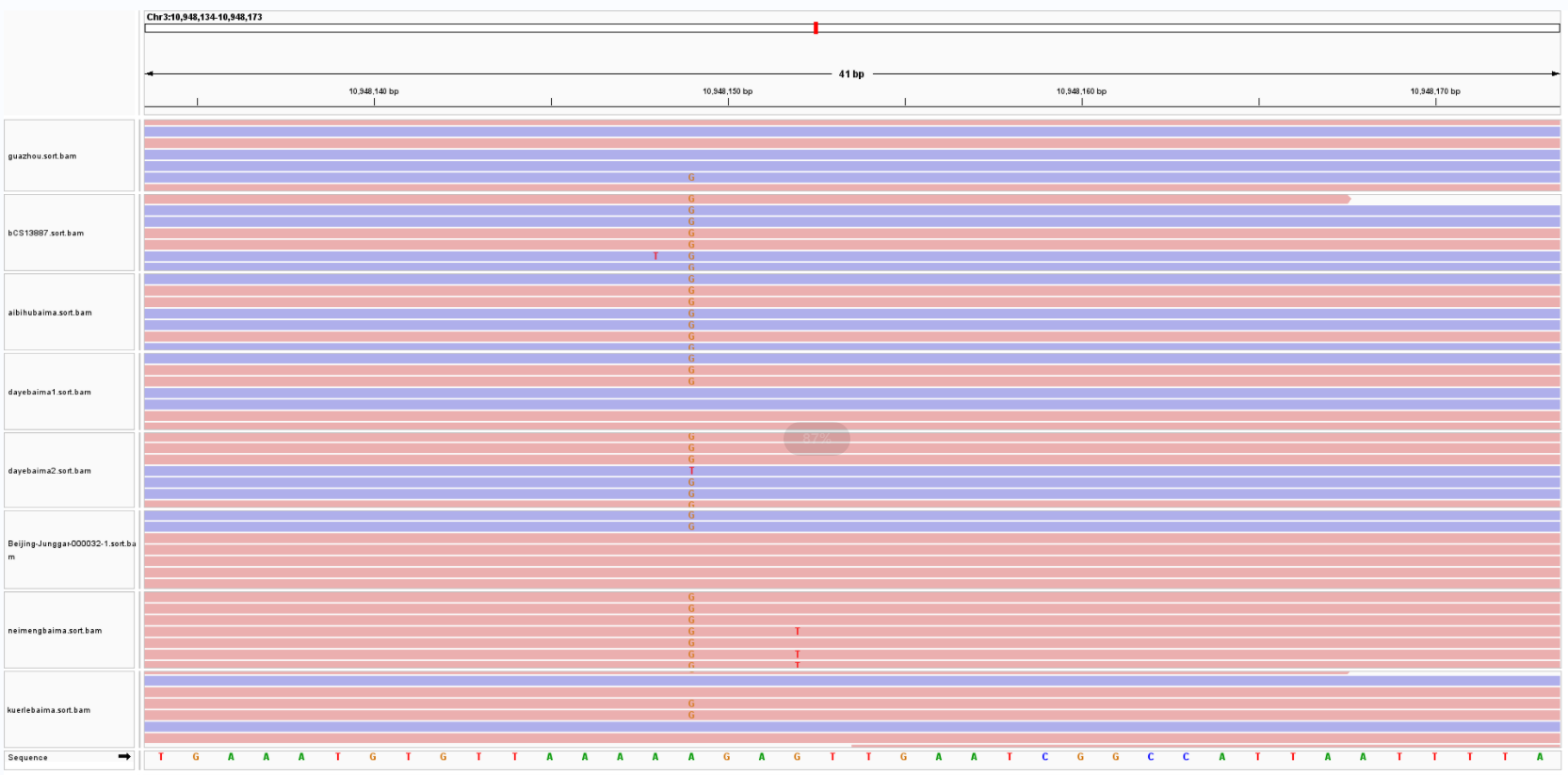


C


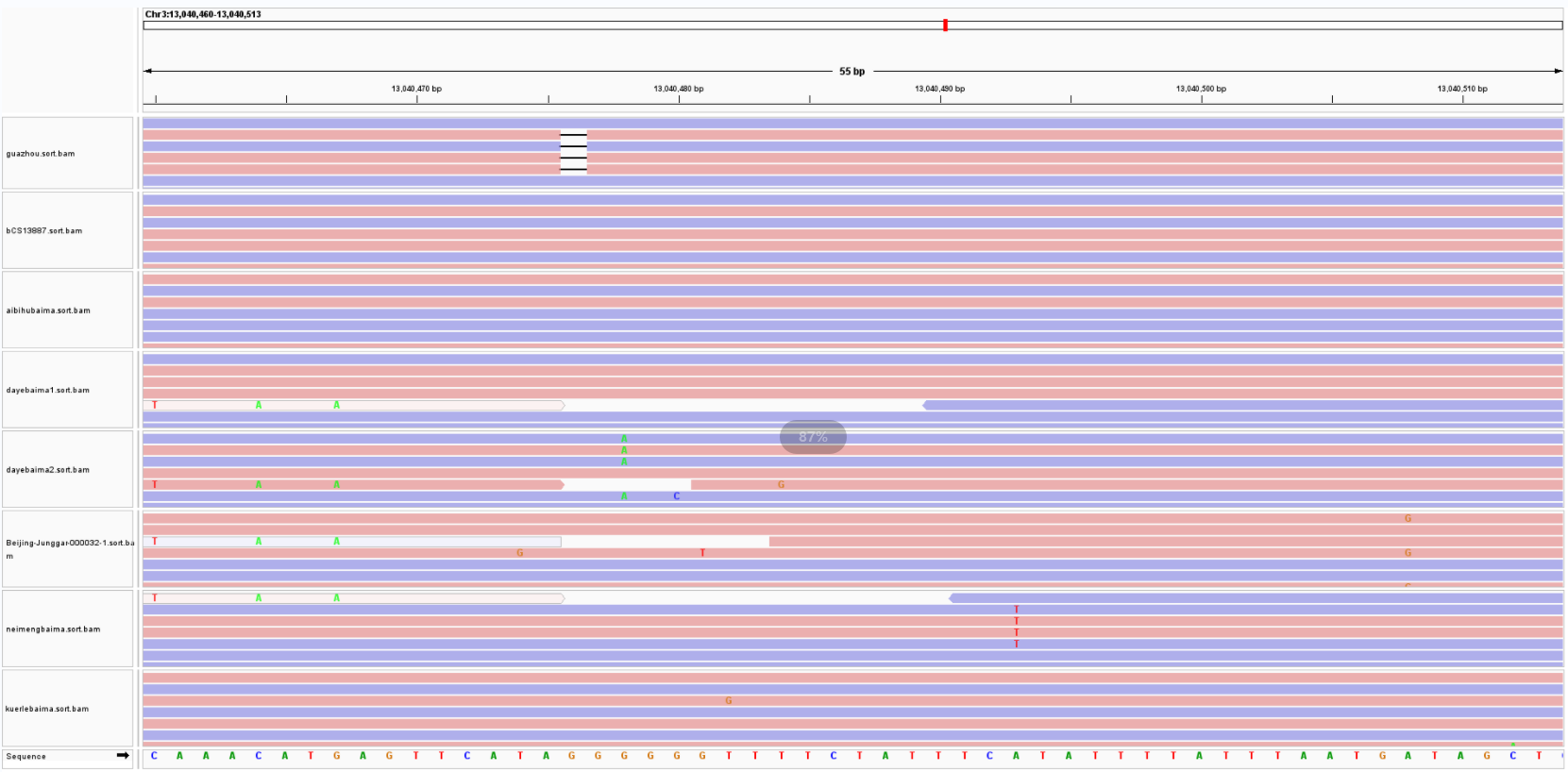


D


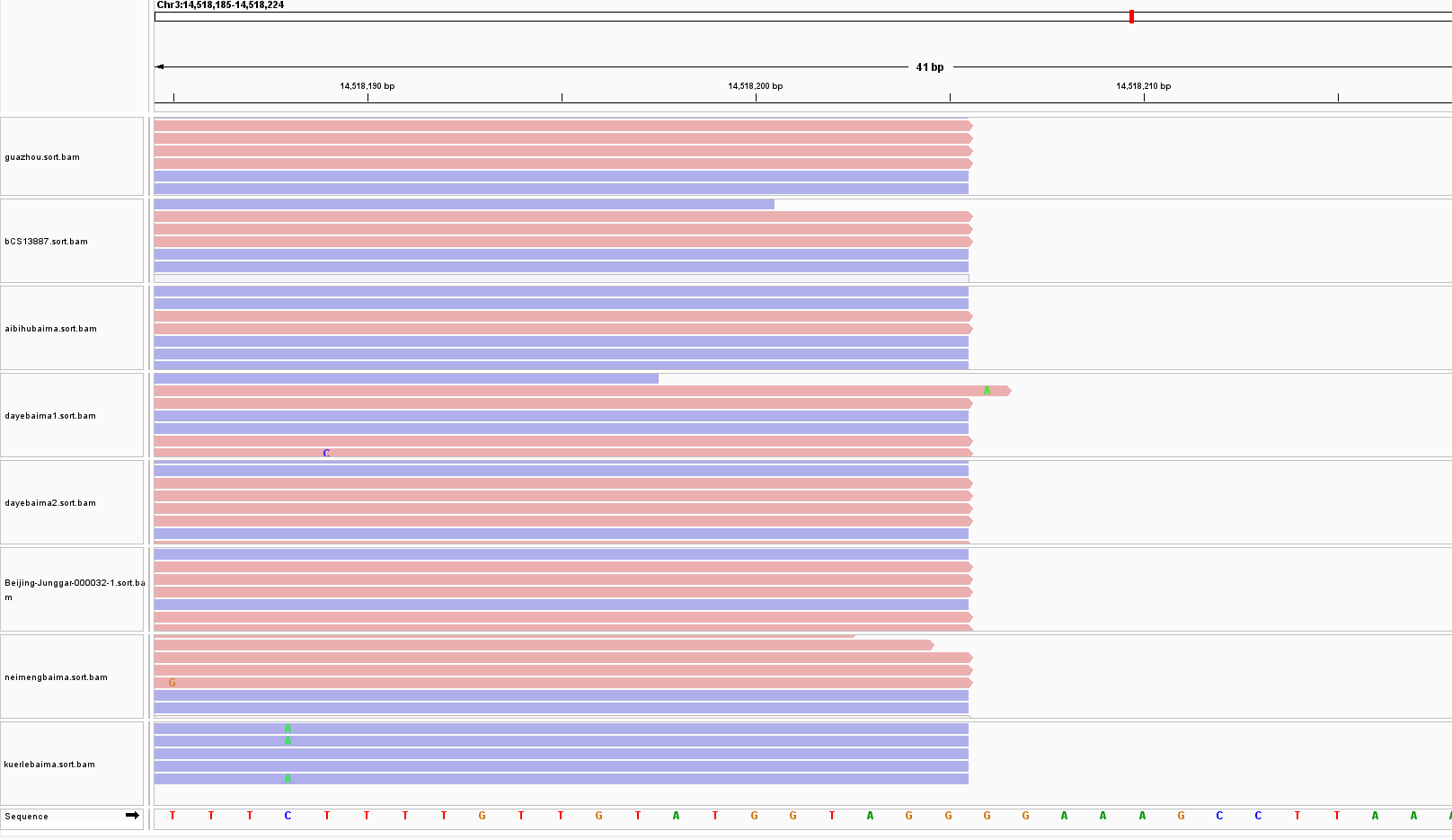


E


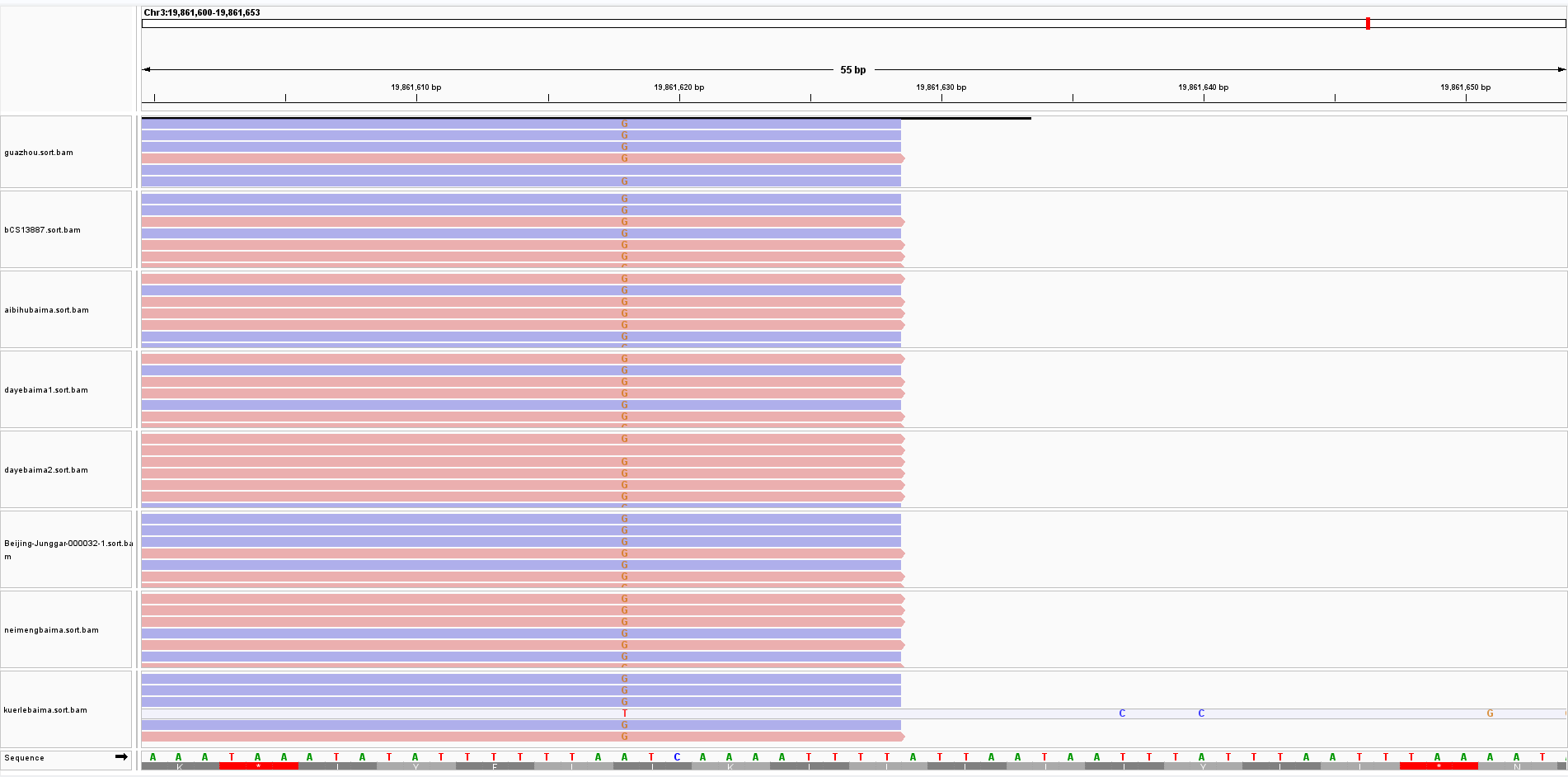


F


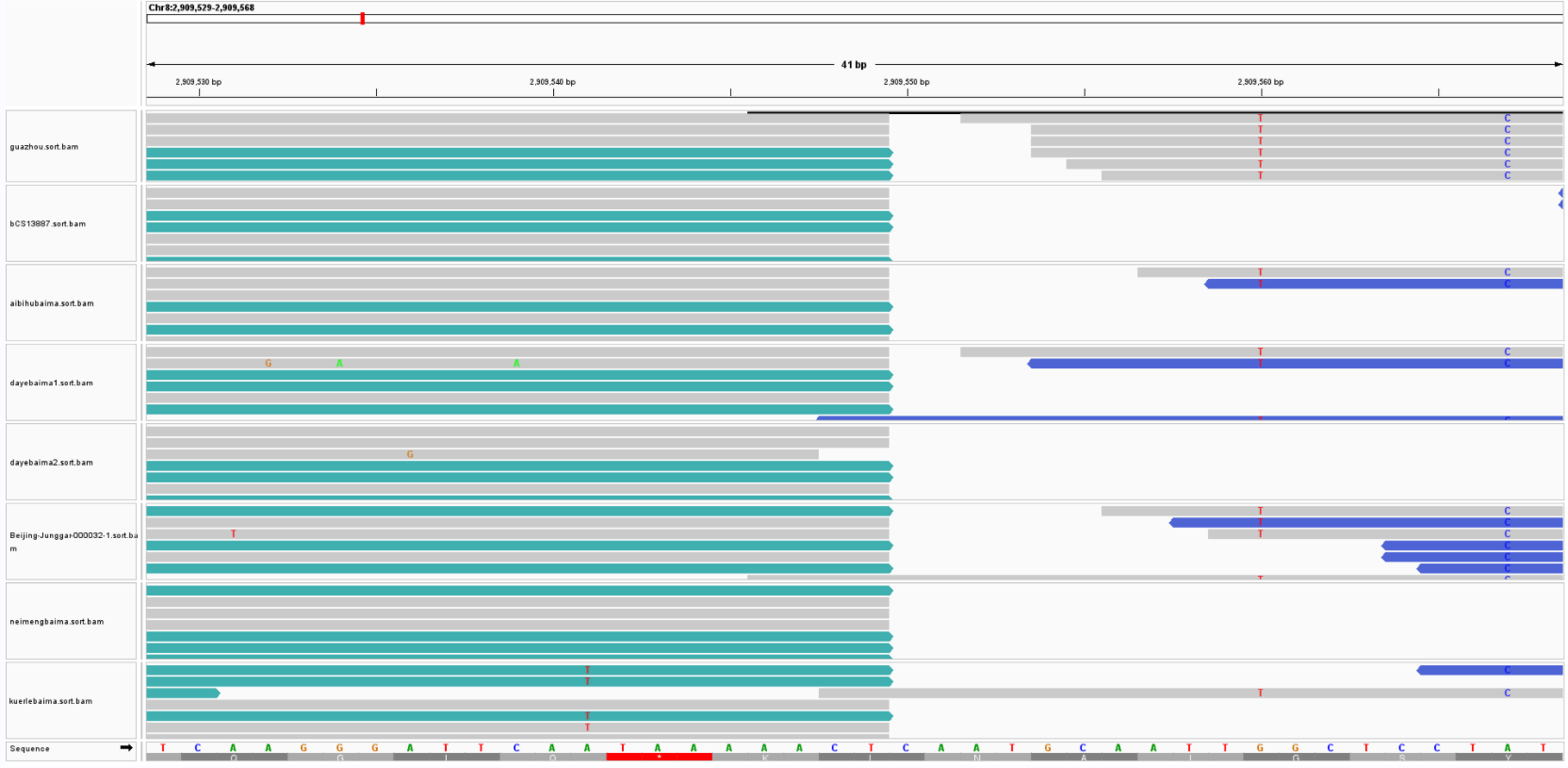


G


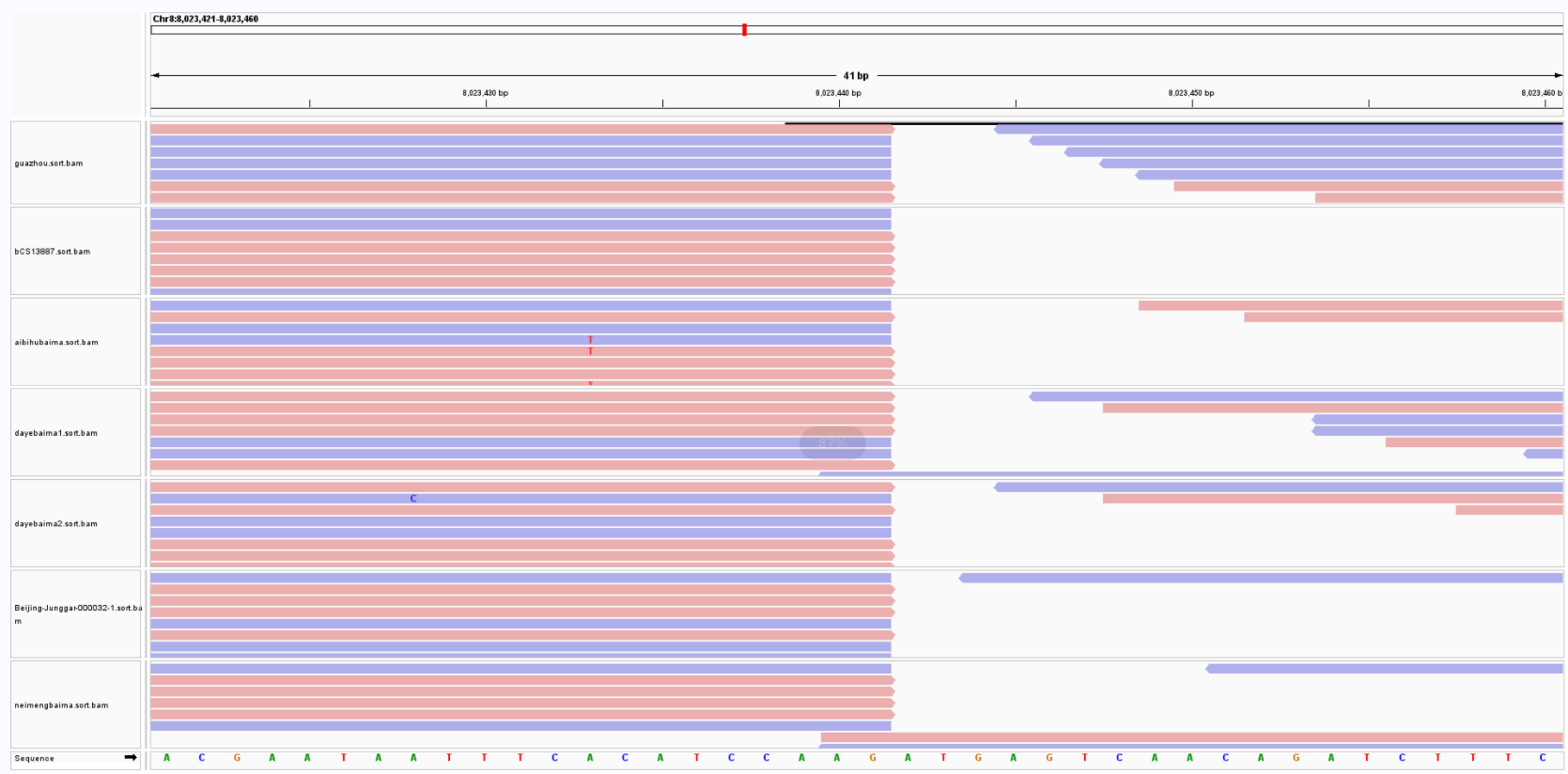


Fig. S21 The inversion points of Chr3 and Chr8 by mapping the raw sequencing reads and visualized by IGV in AHG groups. A, Chr3:7221526; B, Chr3:10948154; C, Chr3:13040487; D, Chr3:13040487; E, Chr3:19861359; F, Chr8:2909549; G, Chr8:8023441.

A


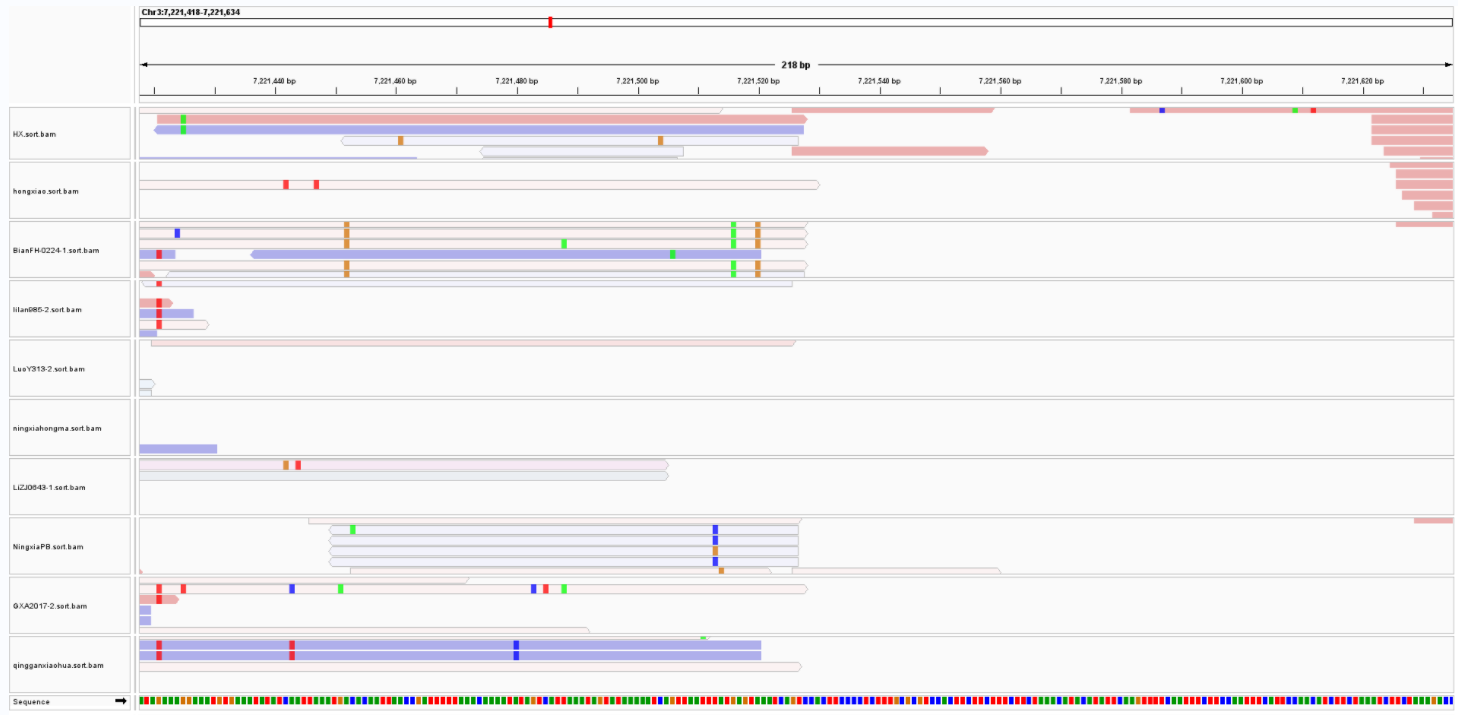


B


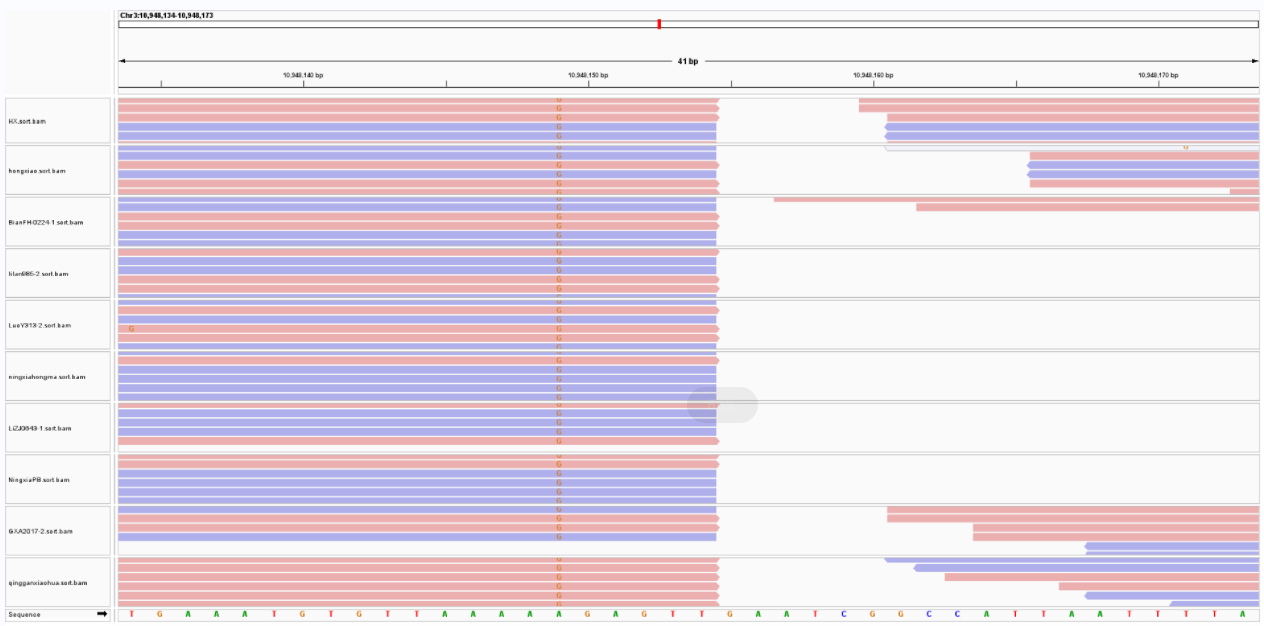


C


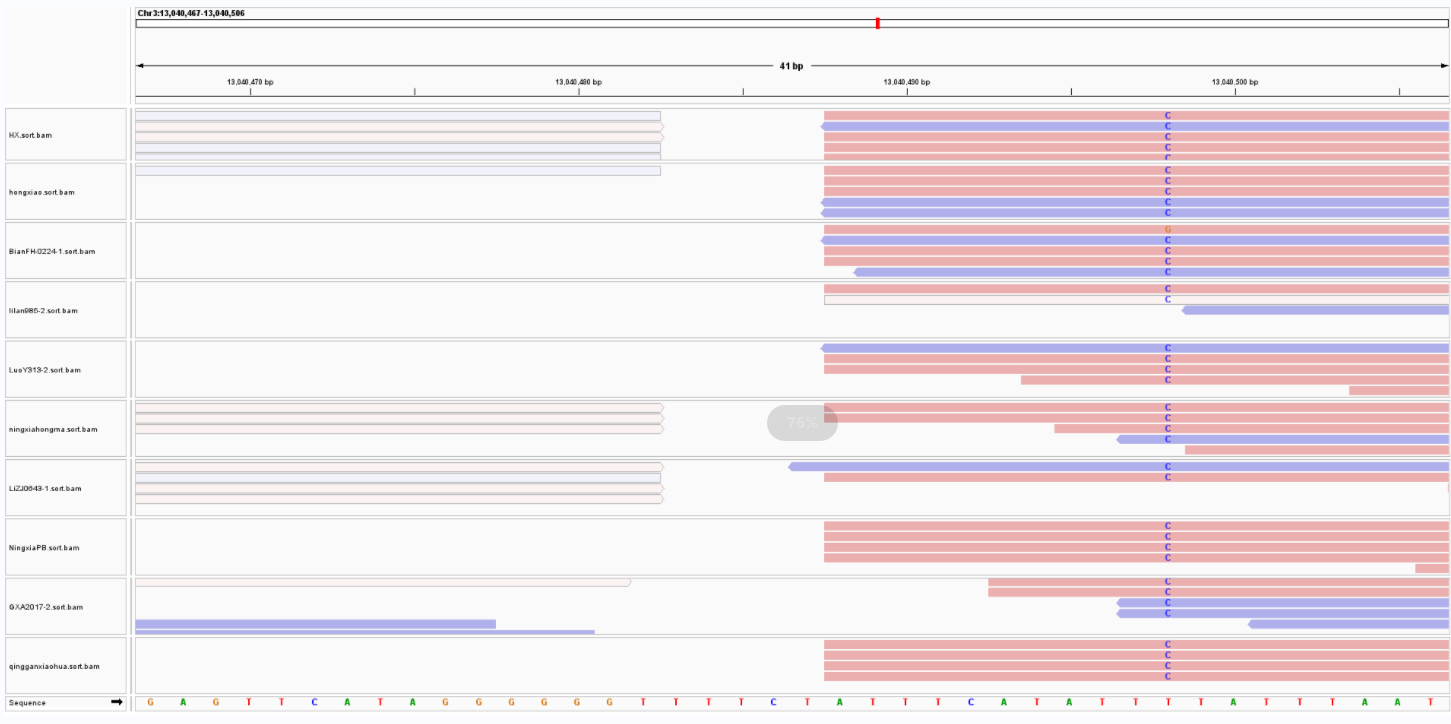


D


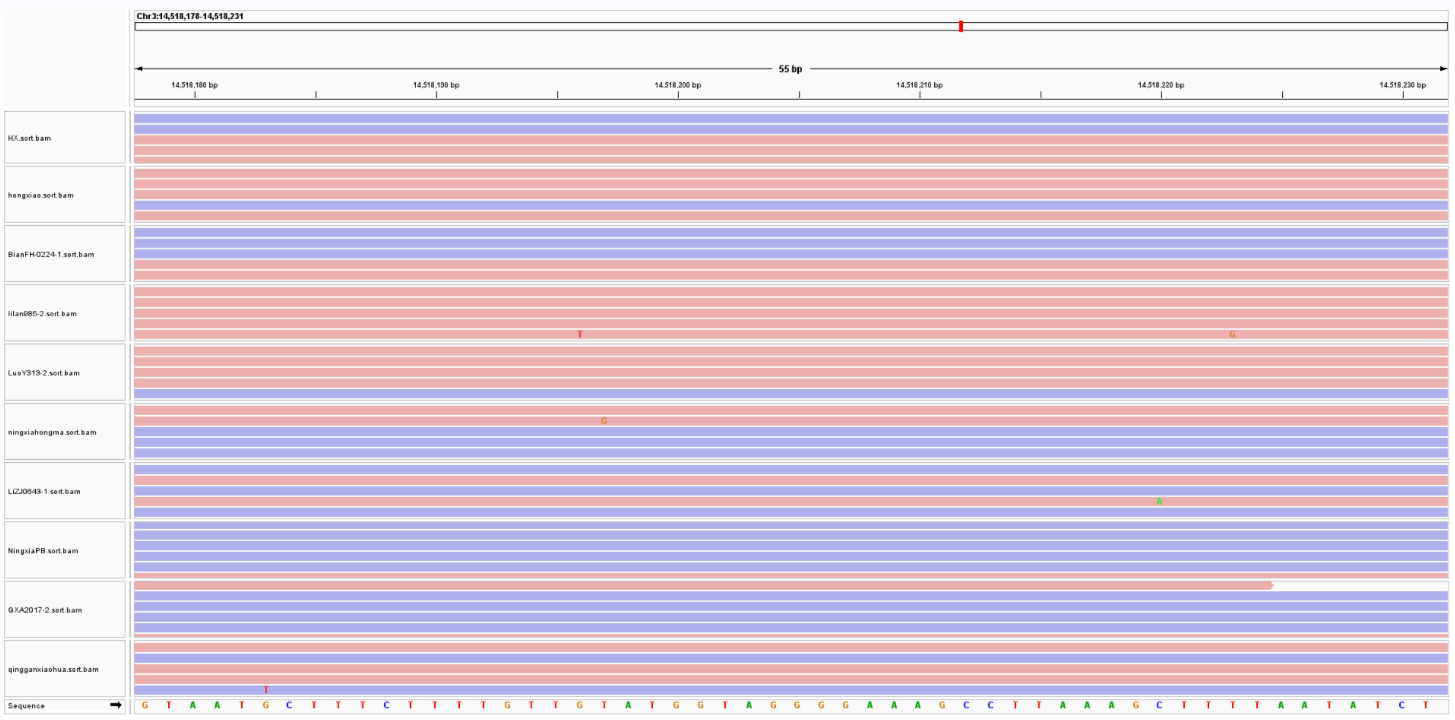


E


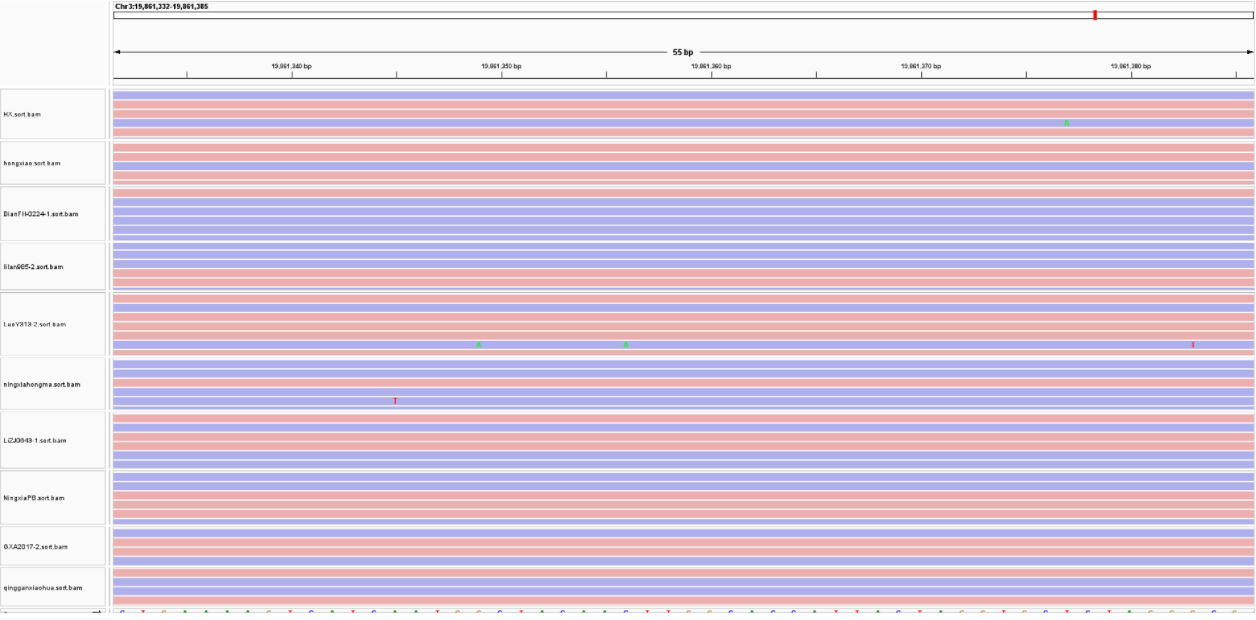


F


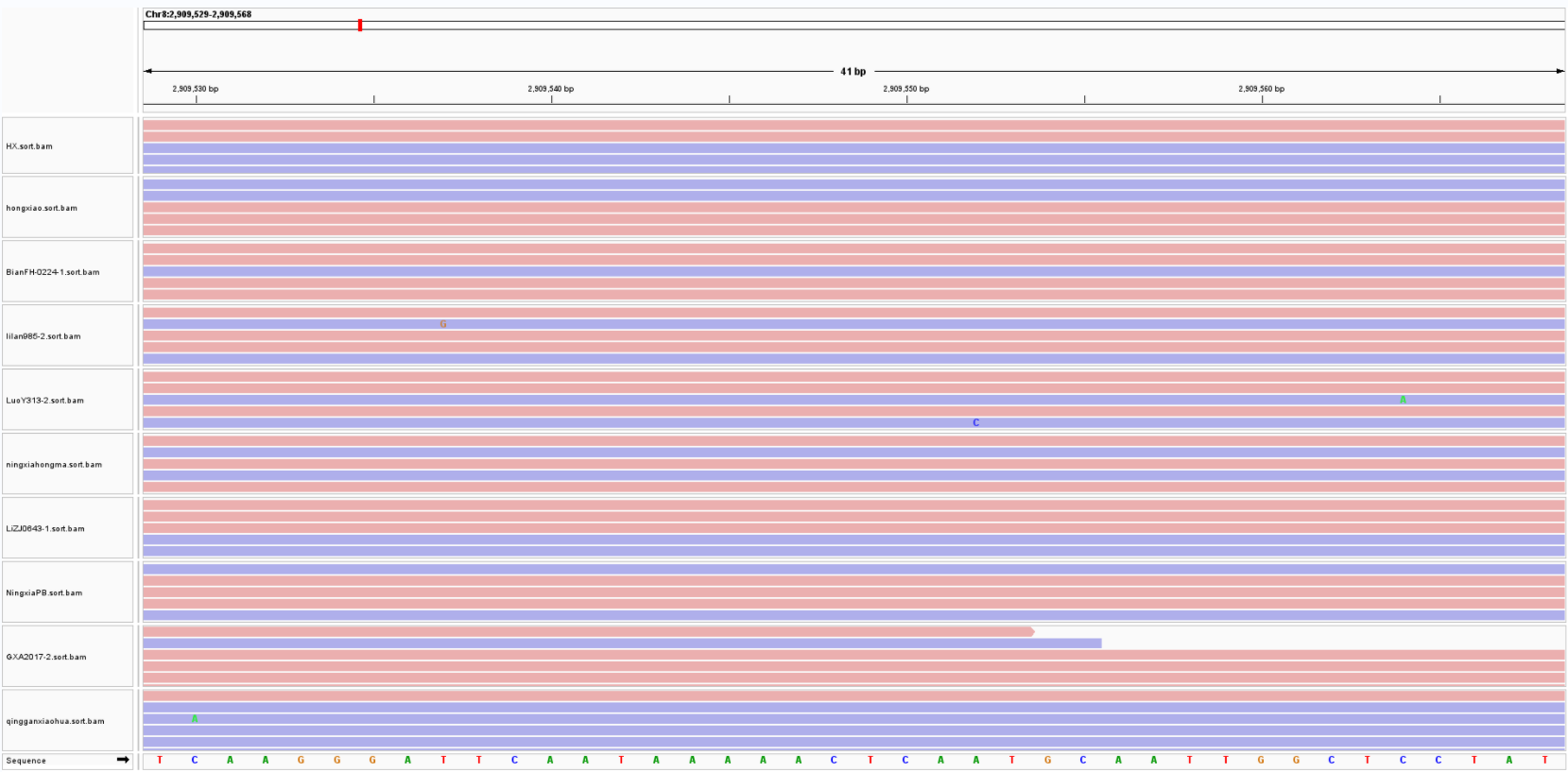


G


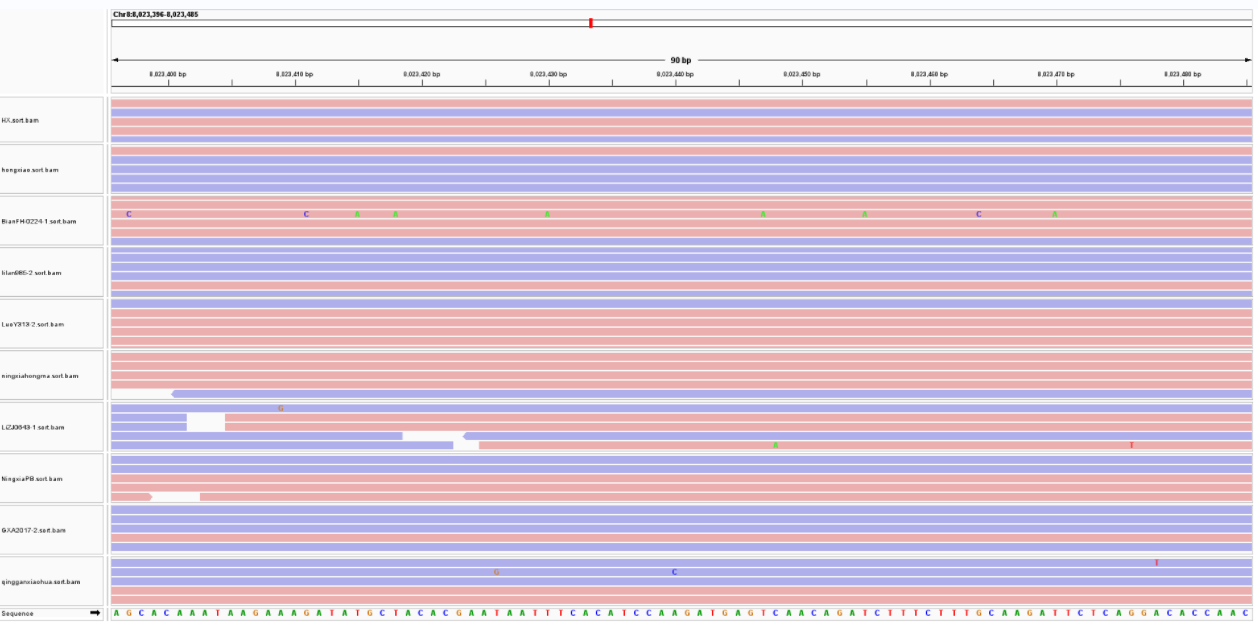


Fig. S22 The inversion points of Chr3 and Chr8 by mapping the raw sequencing reads and visualized by IGV in AVX groups. A, Chr3:7221526; B, Chr3:10948154; C, Chr3:13040487; D, Chr3:13040487; E, Chr3:19861359; F, Chr8:2909549; G, Chr8:8023441.


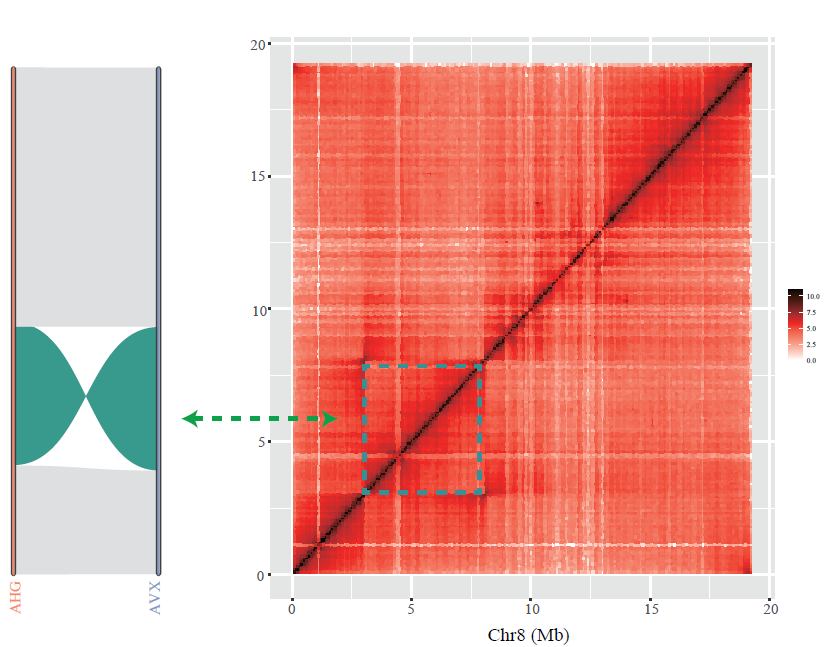


Fig. S23 The AHG Hi-C data mapped to APX genome. The inversions between AHG and AVX on Chr8


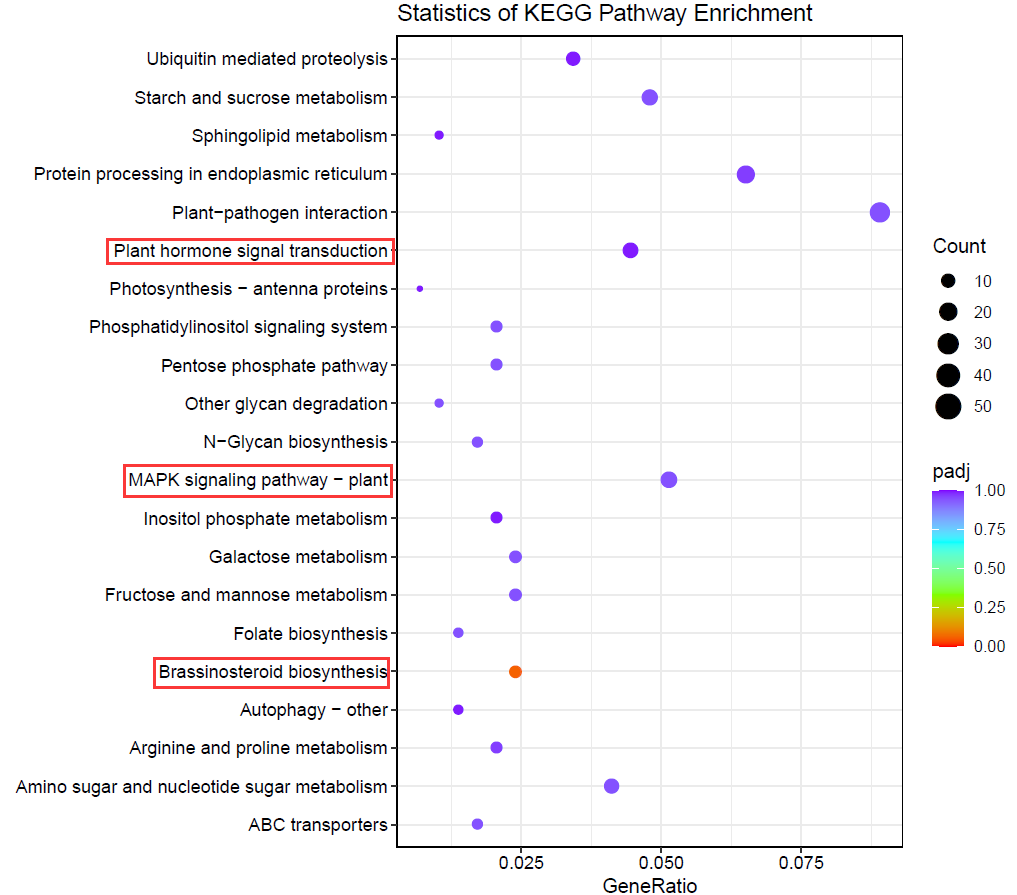


Fig. S24 KEGG enrichment of inversion regions in APZ


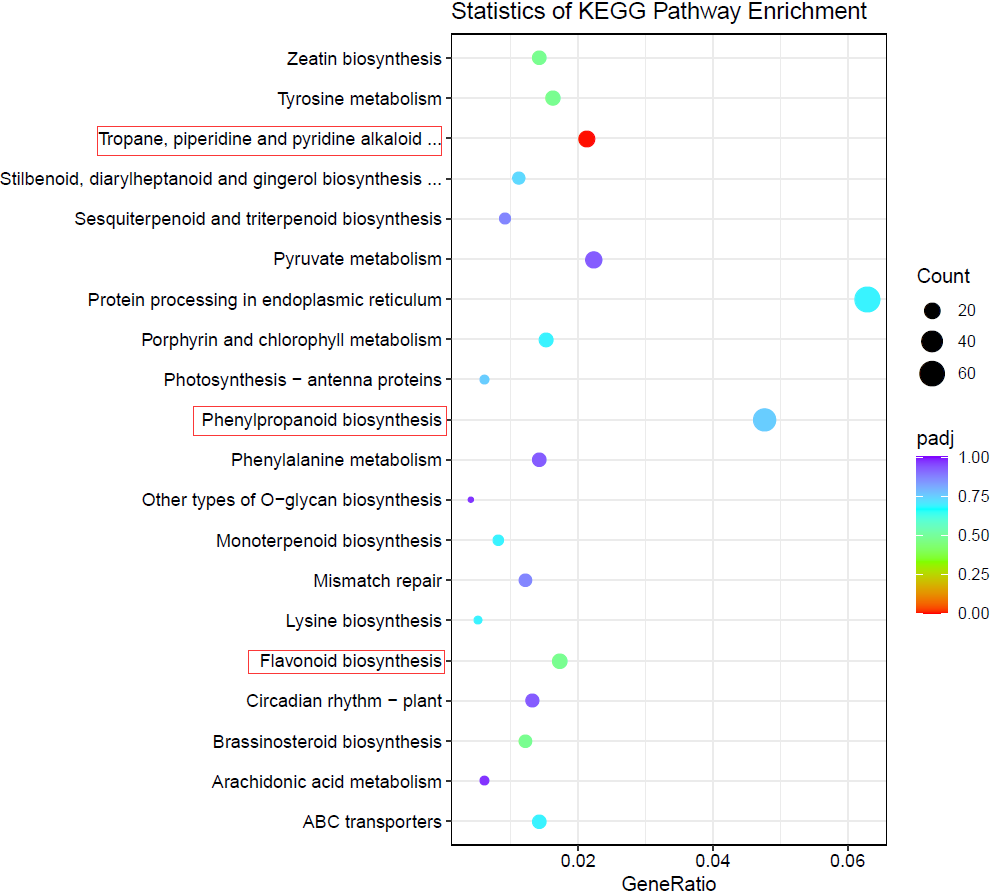


Fig. S25 KEGG enrichment of Smaller SVs in *Apocynum*


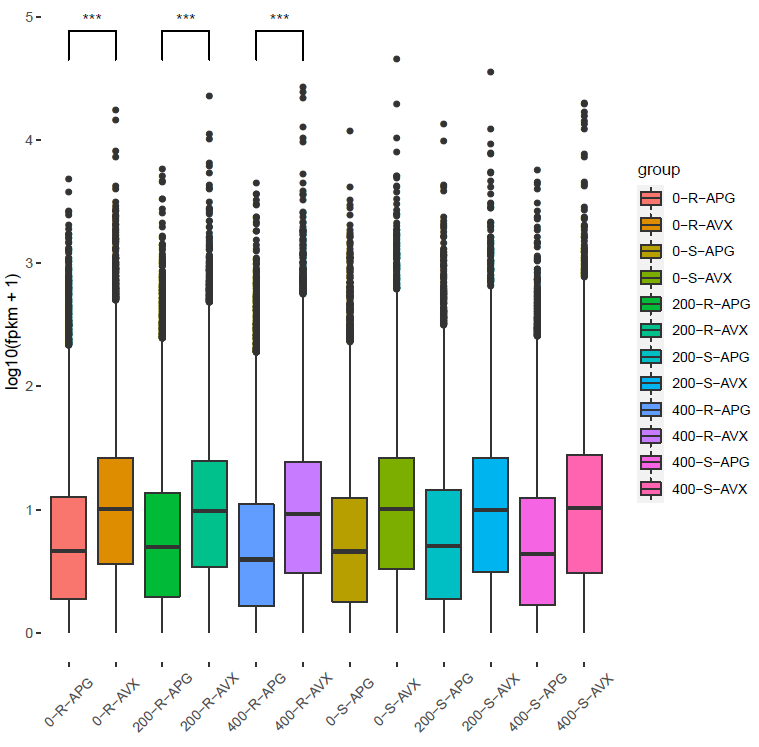


Fig. S26 The allele expression levels bias parental under different salt stress conditions in APZ


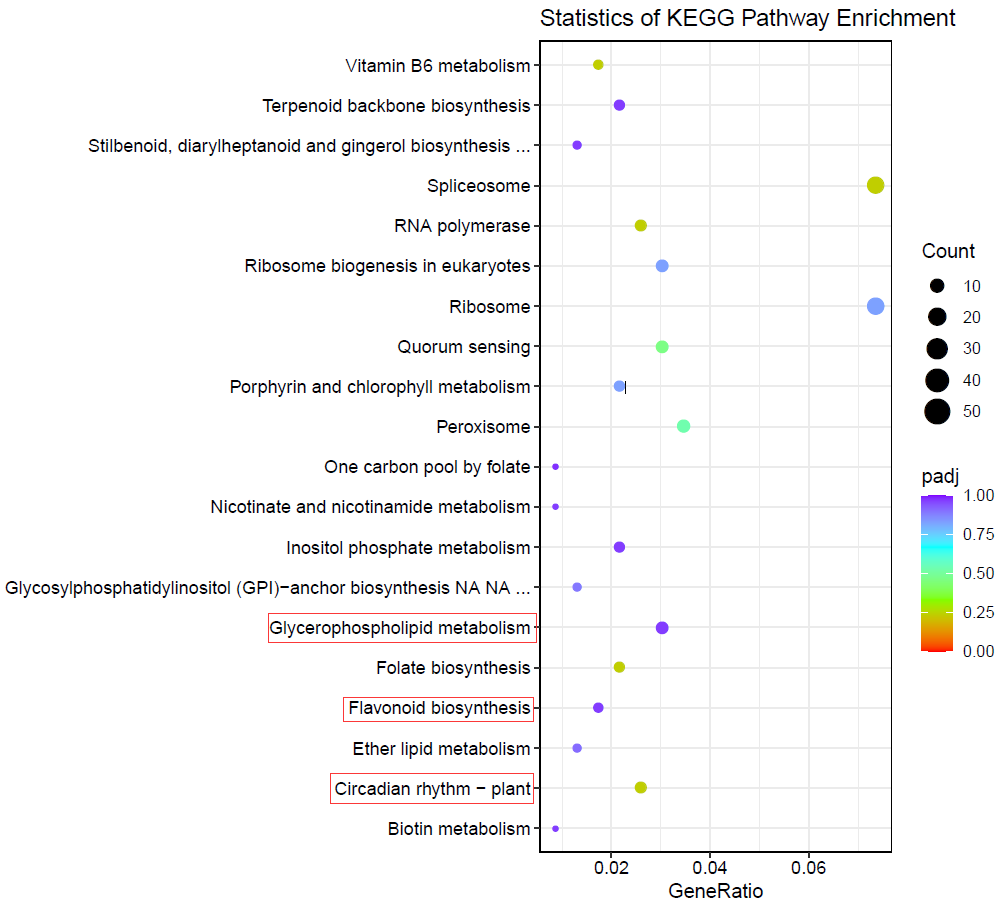


Fig. S27 KEGG enrichment of consistent ASEGs in APZ


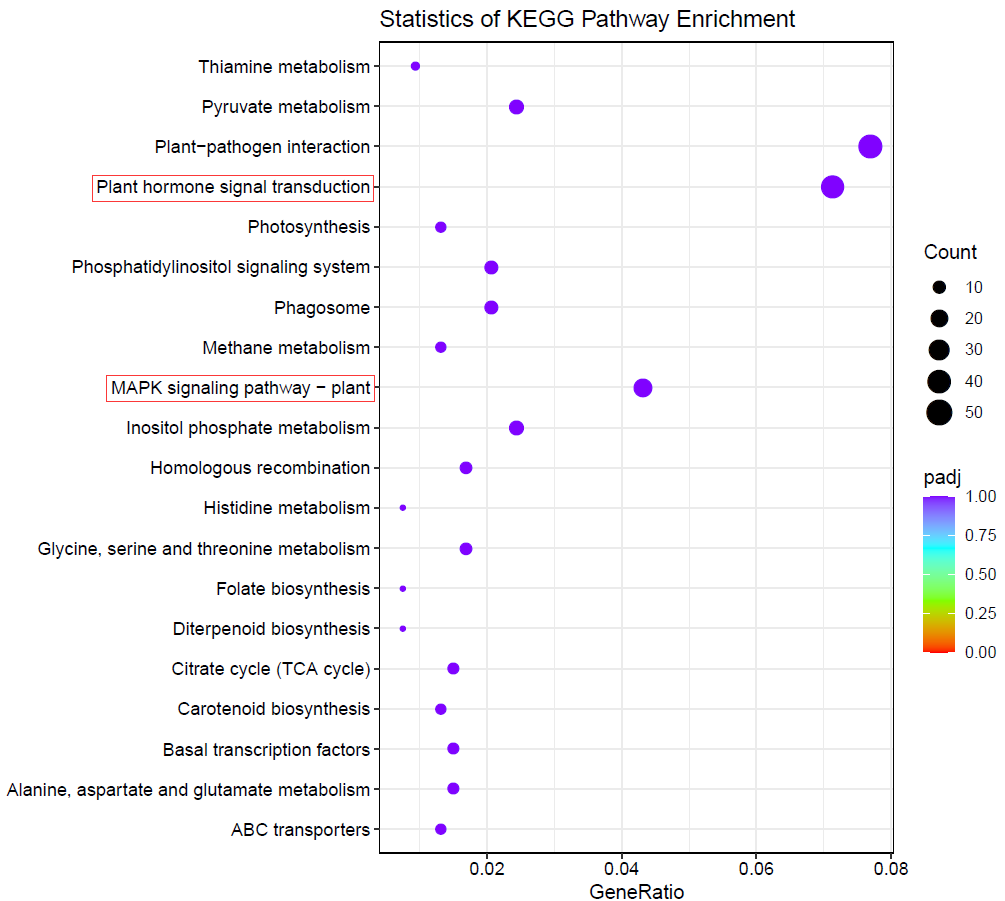


Fig. S28 KEGG enrichment of direction shifting ASEGs in APZ


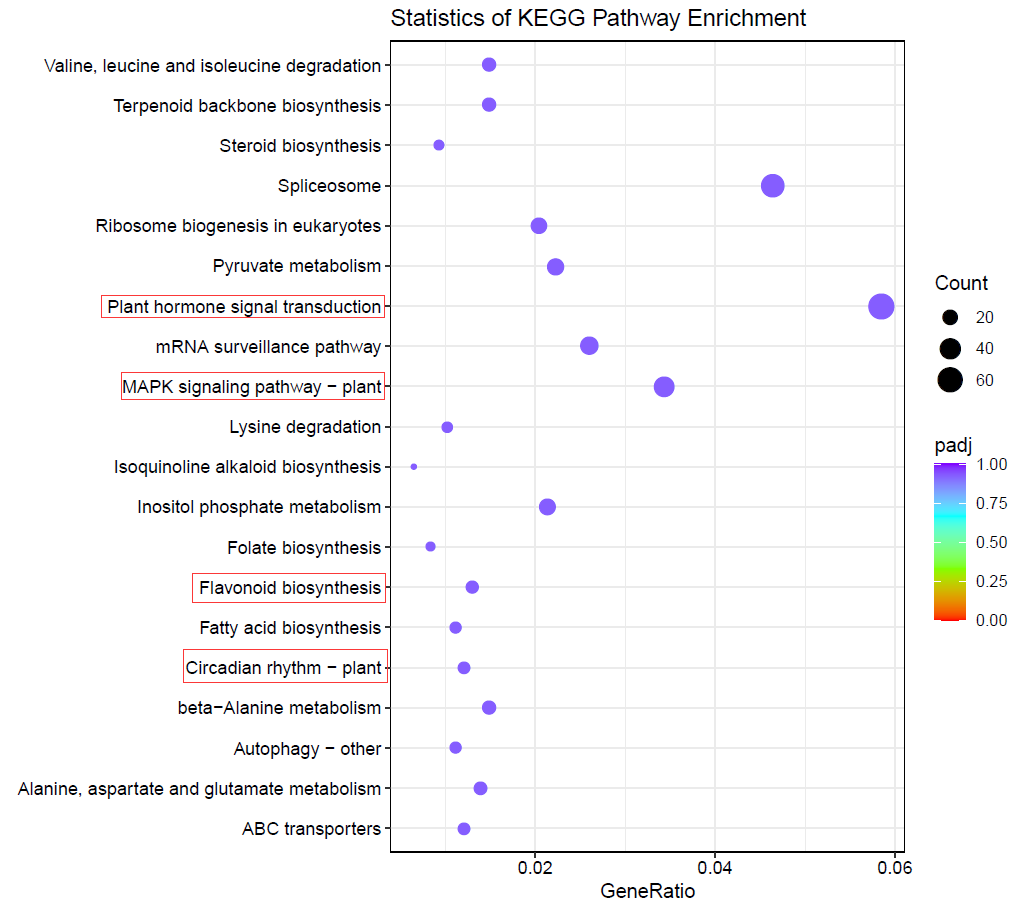


Fig. S29 KEGG enrichment of all ASEGs in APZ


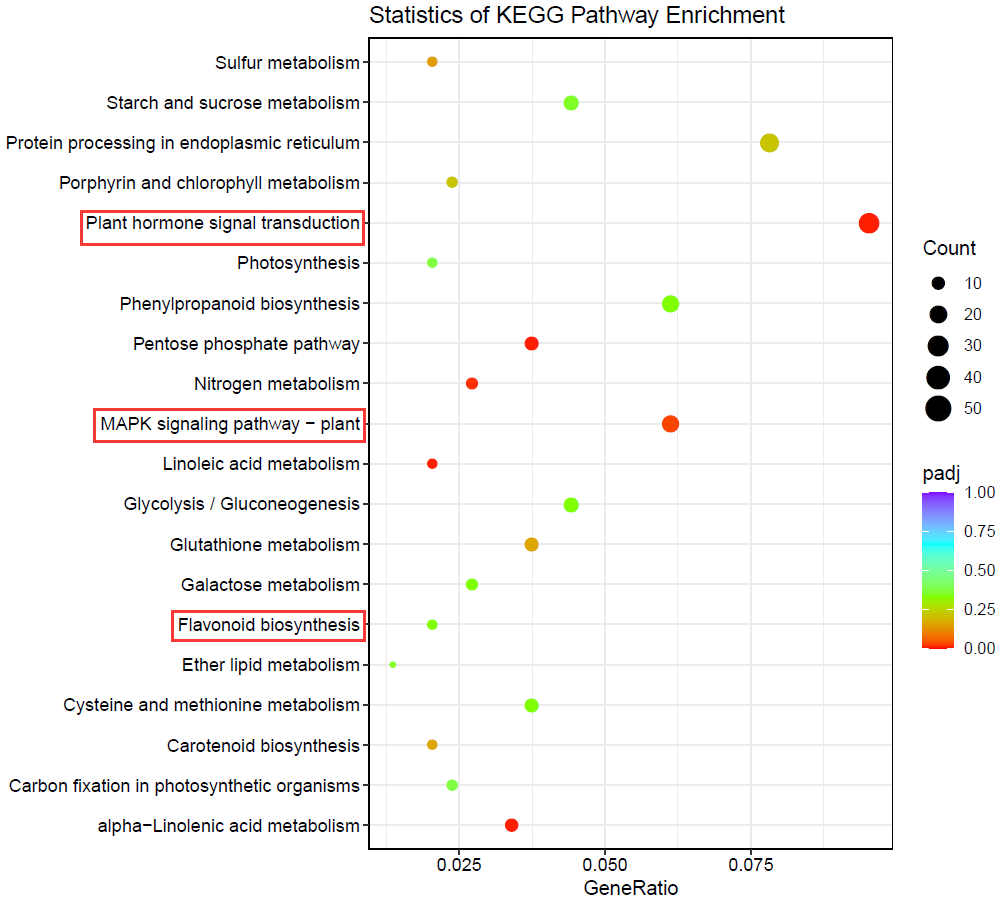


Fig. S30 KEGG enrichment of differential gene expression under 200 mM in the root and shoot of APZ


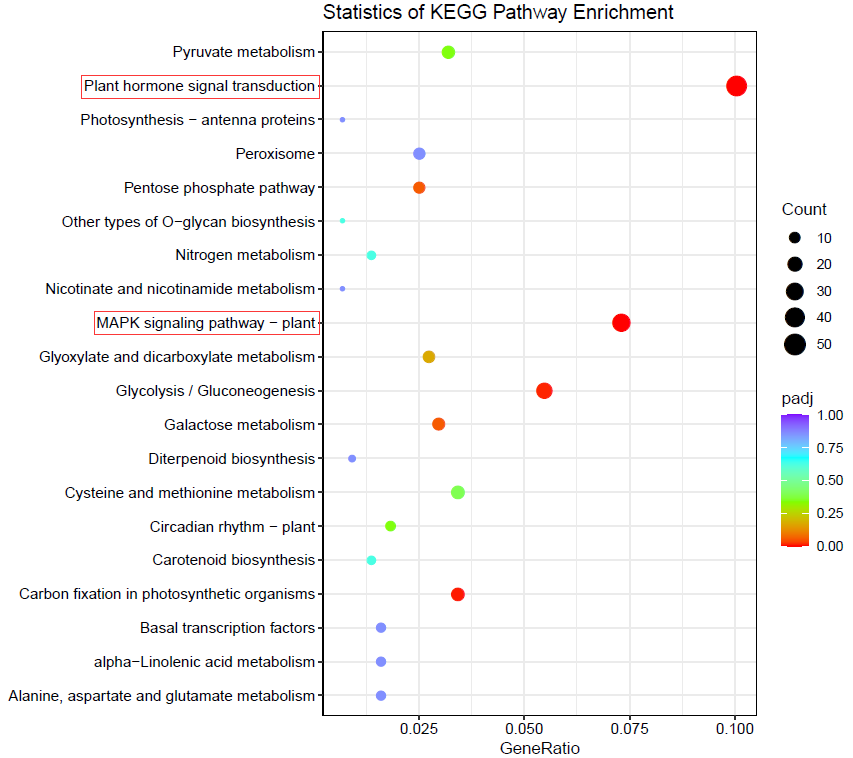


Fig. S31 KEGG enrichment of differential gene expression under 200 mM in the root of AHG


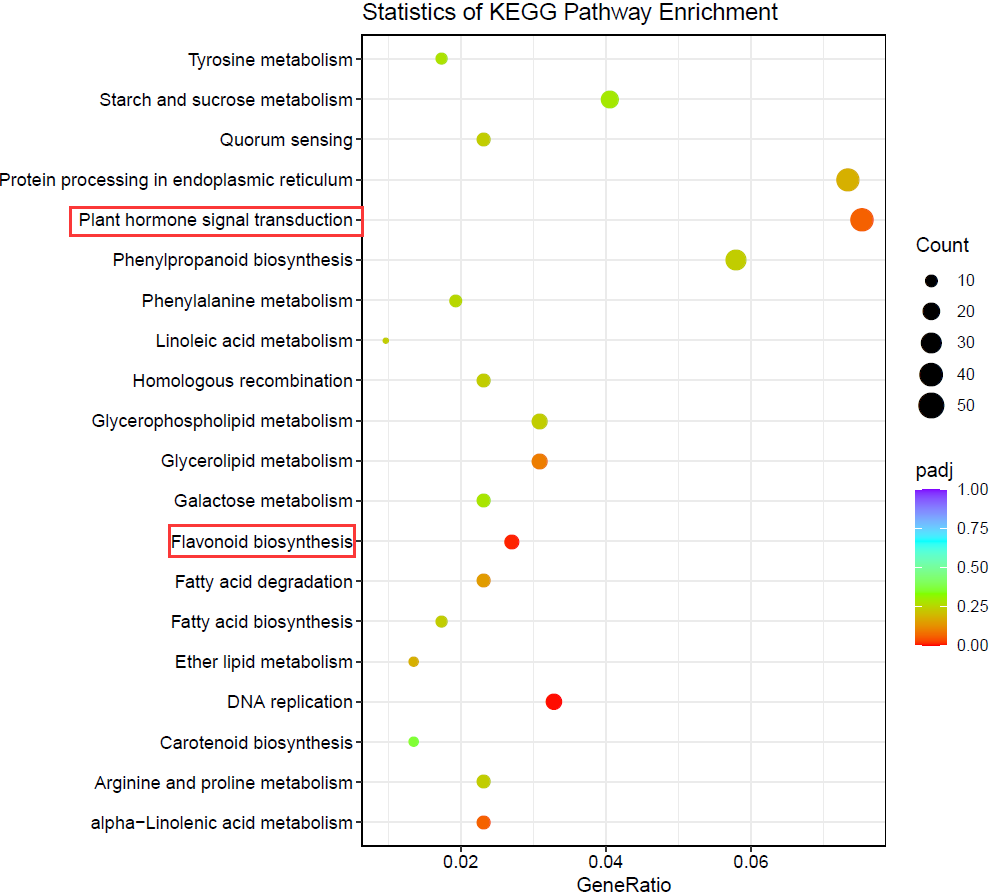


Fig. S32 KEGG enrichment of differential gene expression under 400 mM in the root and shoot of APZ


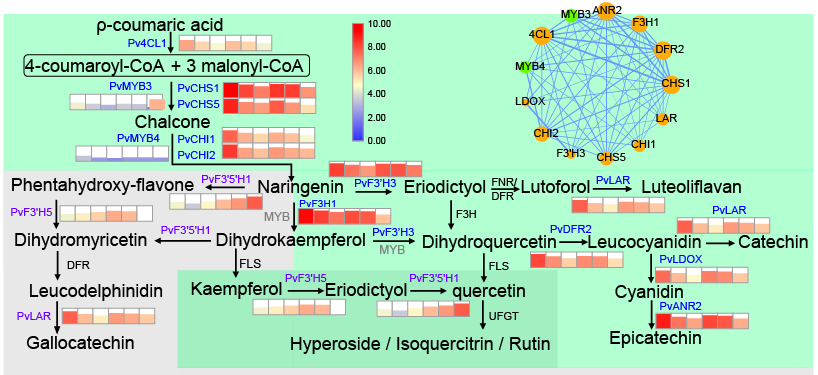


Fig. S33 The putative flavonoid biosynthetic pathway based on the expression profiles of each candidate gene under salt stress


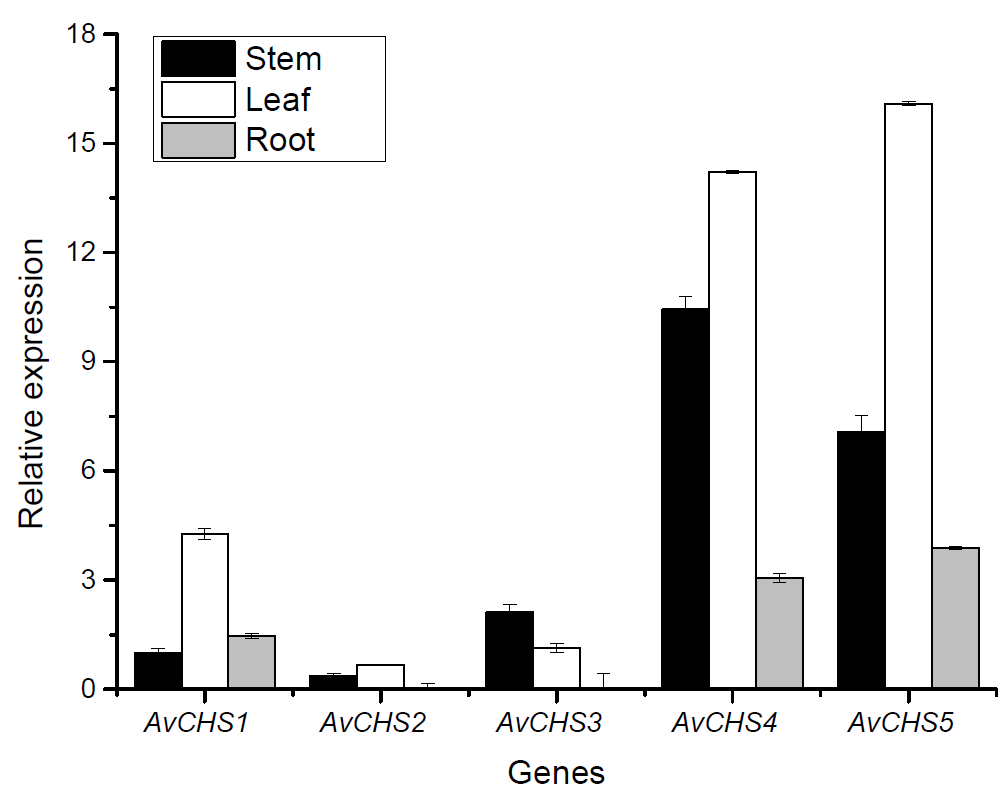


Fig. S34The relative expression level of *AvCHS*s in stem, leaf and root of AVX under salt stress


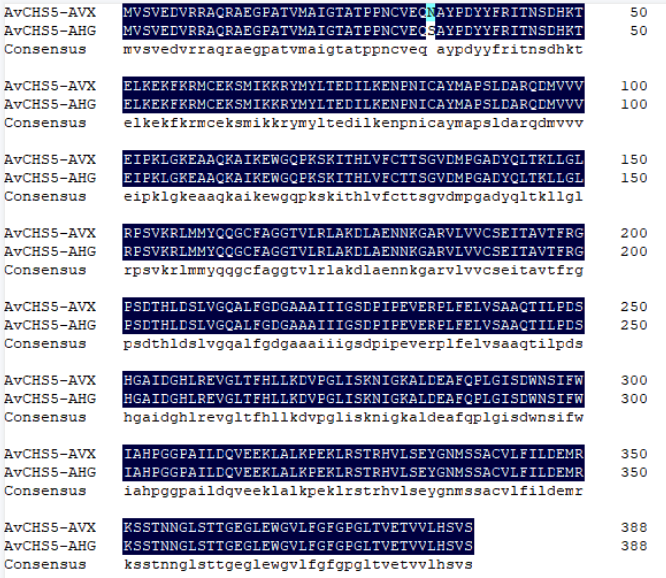


Fig. S35 The Protein alignment of *AvCHS5* alleles in APZ under salt stress

A B


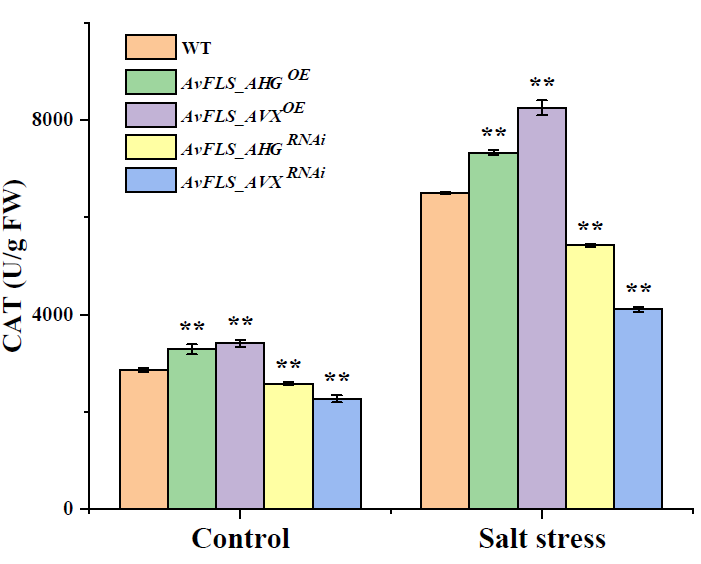

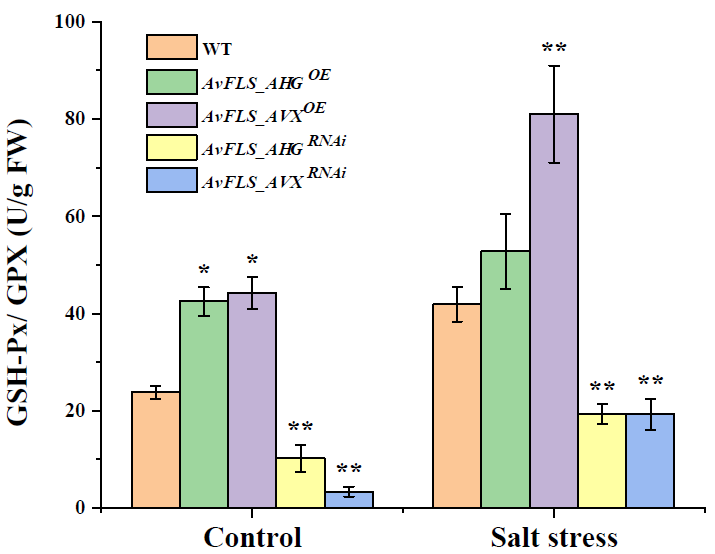


C D


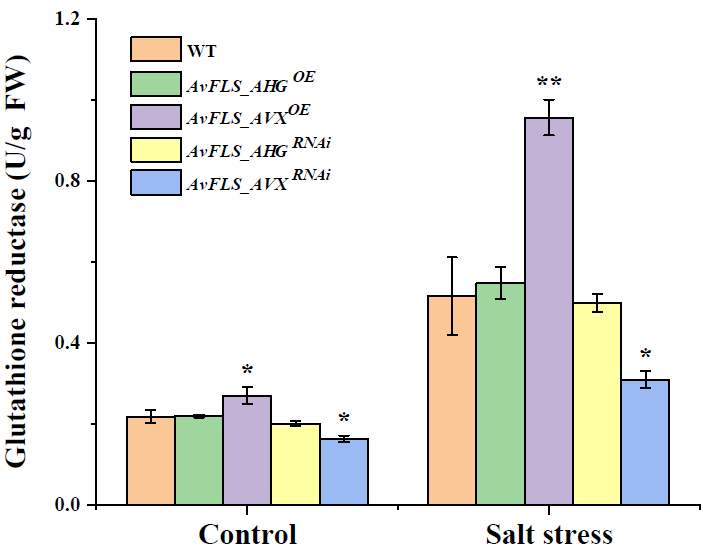

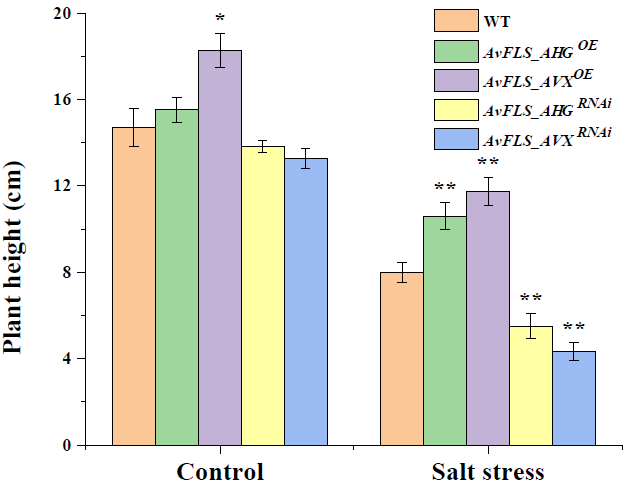


E F


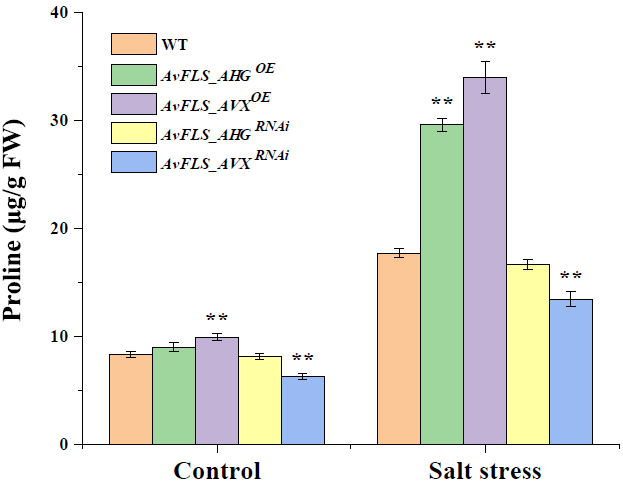

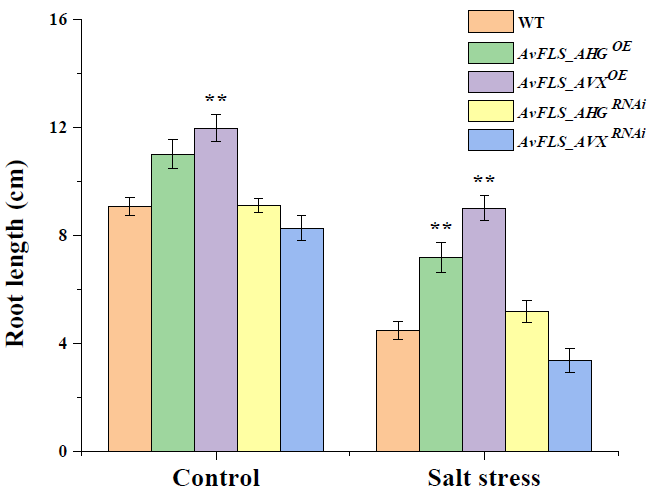


G H


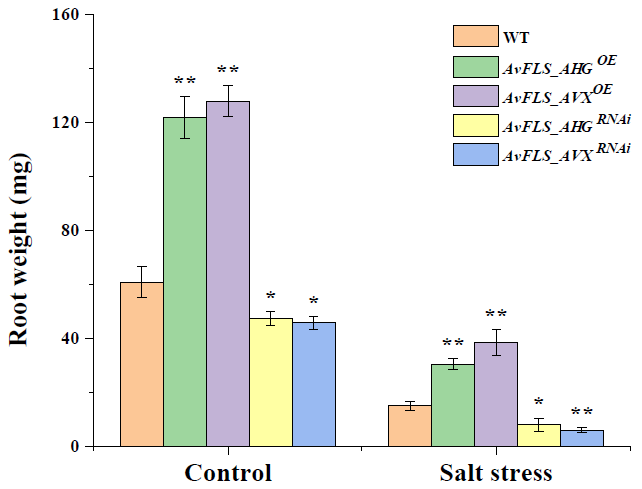

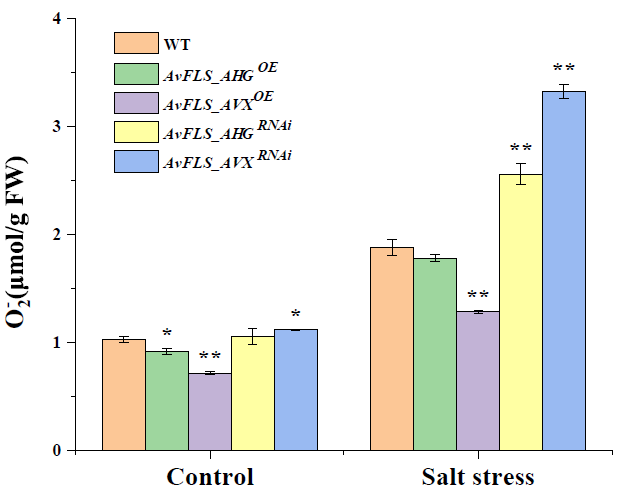


I J


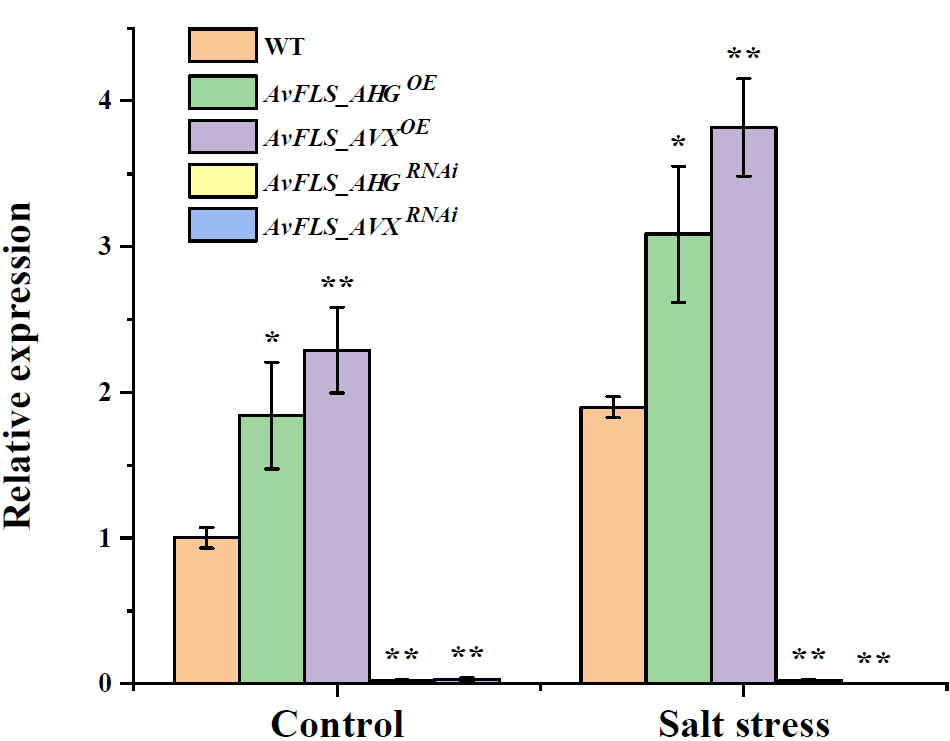

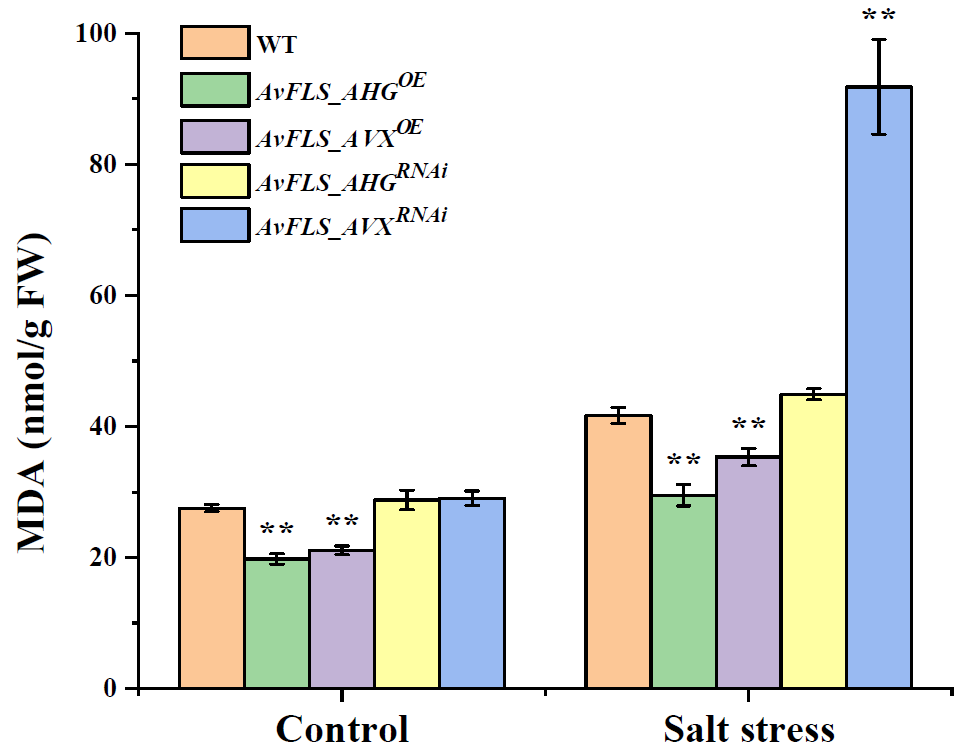


Fig. S36 The physiological indicators of transgenic root hairs of *AvFLS* under salt stress


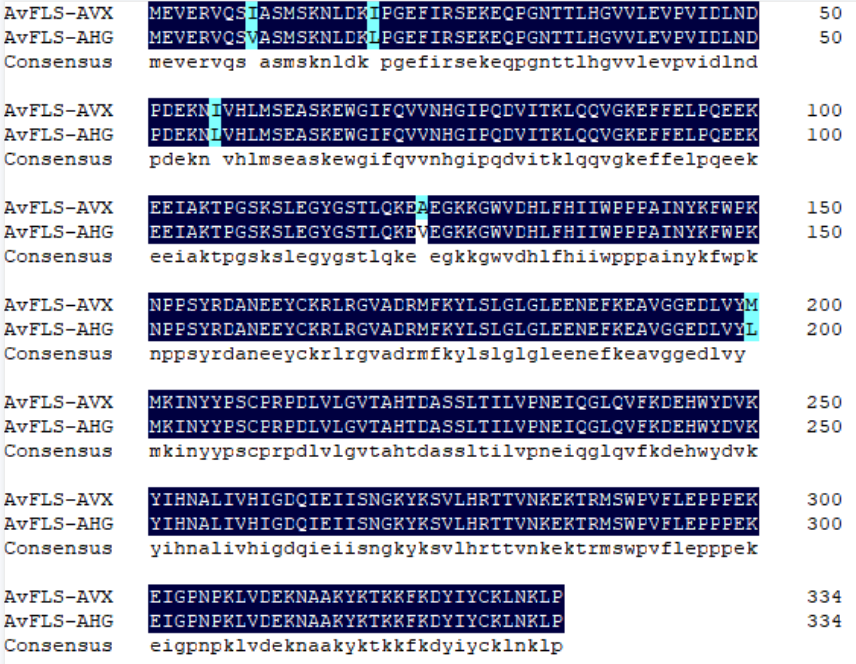


Fig. S37 The Protein alignment of *AvFLS* of AVX and AHG
